# Synthesis and Biological Profiling of Seven Heparin and Heparan Sulphate Analogue Trisaccharides

**DOI:** 10.3390/biom14091052

**Published:** 2024-08-25

**Authors:** Fruzsina Demeter, Zsófia Peleskei, Katalin Kútvölgyi, Ágnes Rusznyák, Ferenc Fenyvesi, Richárd Kajtár, Éva Sipos, István Lekli, Petra Molnár, Attila Gábor Szöllősi, Erika Lisztes, Balázs István Tóth, Anikó Borbás, Mihály Herczeg

**Affiliations:** 1 Department of Pharmaceutical Chemistry, Faculty of Pharmacy, University of Debrecen, Egyetem tér 1, H-4032 Debrecen, Hungary; demeter.fruzsina@science.unideb.hu (F.D.); peleskei.zsofia@pharm.unideb.hu (Z.P.); kutvolgyi.katalin@pharm.unideb.hu (K.K.); borbas.aniko@pharm.unideb.hu (A.B.); 2 Department of Molecular and Nanopharmaceutics, Faculty of Pharmacy, University of Debrecen, Nagyerdei Körút 98, H-4032 Debrecen, Hungary; rusznyak.agnes@pharm.unideb.hu (Á.R.); fenyvesi.ferenc@pharm.unideb.hu (F.F.); 3 Institute of Healthcare Industry, University of Debrecen, Egyetem tér 1, H-4032 Debrecen, Hungary; 4 Department of Pharmacodynamics, Faculty of Pharmacy, University of Debrecen, Nagyerdei Körút 98, H-4032 Debrecen, Hungary; kajtar.richard@euipar.unideb.hu (R.K.); sipos.eva@pharm.unideb.hu (É.S.); lekli.istvan@pharm.unideb.hu (I.L.); 5 Department of Immunology, University of Debrecen, Egyetem tér 1, H-4032 Debrecen, Hungary; molnar.petra@med.unideb.hu (P.M.); szollosi.attila@med.unideb.hu (A.G.S.); 6 Department of Physiology, University of Debrecen, P.O. Box 22, H-4012 Debrecen, Hungary; lisztes.erika@med.unideb.hu (E.L.); toth.istvan@med.unideb.hu (B.I.T.); 7 Department of Physiology, Medical School, University of Pécs, Szigeti út 12, H-7624 Pécs, Hungary; 8 HUN-REN-DE Molecular Recognition and Interaction Research Group, University of Debrecen, Egyetem tér 1, H-4032 Debrecen, Hungary

**Keywords:** heparin, heparan sulphate, trisaccharides, anti-inflammatory, antioxidant

## Abstract

Researchers are paying increasing attention to the strongly negatively charged heteropolysaccharides in cells, in the extracellular matrix or in the cell wall. Examples of such molecules are glycosaminoglycans (e.g., heparin, heparan sulphate). It is well known from the literature that heparin and its derivatives have anti-inflammatory, angiogenic, metastatic and growth factor inhibitory activity. Herein, we present the efficient synthesis of six non-glycosaminoglycan (Glc-GlcA-Glc-sequenced) and one heparin-related (GlcN-GlcA-Glc-sequenced) trisaccharides with various functional group patterns. The anti-inflammatory, antioxidant and cell growth-inhibitory/cytotoxic effects of the synthesized compounds were tested. Among the investigated molecules, we have found some derivatives with a promising anti-inflammatory and antioxidant effect.

## 1. Introduction

**Figure 1:**
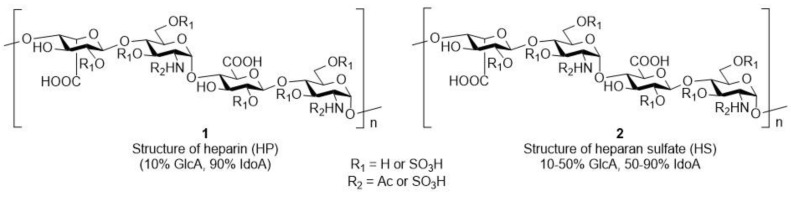
Representative structure of heparin (**1**) and heparan sulphate (**2**).

Recently, researchers have been paying special attention to the strongly negatively charged polysaccharides and higher oligosaccharides found on the cell, cell surface or extracellular matrix due to their diverse biological roles. Of particular importance among these biopolymers are glycosaminoglycans (GAGs), which are linear, high-molecular-weight polydisperse heteropolysaccharides composed of repeating disaccharide units [1,2,3]. One component of the repeating disaccharide units is always *N*-acetylglucosamine or *N*-acetylgalactosamine and the other building block is a uronic acid (e.g., d-glucuronic acid or l-iduronic acid). Glycosaminoglycans often contain sulphate ester groups in a variety of patterns [4,5]. Among the GAGs, heparin (**1**, HP)/heparan sulphate (**2**, HS) (Figure 1), which are very significant molecules, have been used as anticoagulants in medical practice since the late 1930s [6,7]. In addition to inhibiting blood coagulation, these compounds also have many other biological effects [8,9,10,11]. It is well known from the literature that heparin and its derivatives also have anti-inflammatory [12], cardiovascular- and tissue-protective [12], kidney- and nerve-protective [13], angiogenic [14,15,16], and metastasis and growth factor inhibitory [17,18] as well as antiprotozoal [19] and antiviral activity [20,21], and based on some literature analogies, it may even have an antioxidant effect [22,23,24]. It is also known that an oligosaccharide moiety of at least five sugar units is required for the anticoagulant activity. This unique pentasaccharide moiety extracted from the heparin polymer has a specific Xa factor inhibitory effect. However, fragments with a smaller or different structure (di-, tri- and tetrasaccharides) no longer have anticoagulant activity [25,26,27,28,29].

**Figure 2:**
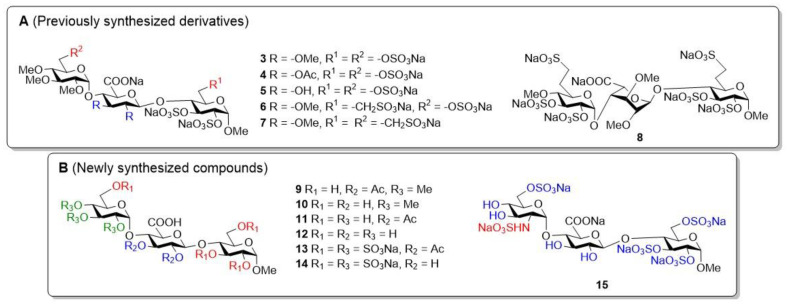
The structures of the previously investigated trisaccharide derivatives ((**A**), **3**–**8**) and the newly synthesized trisaccharide compounds ((**B**), **9**–**15**).

During our previous work, we demonstrated that heparin-analogue small molecules with a simplified structure (Figure 2A, **3**–**8**) can have important biological activity [30]. Two (**4** and **5**) of the six prepared derivatives we produced had good cell growth inhibitory activity; moreover, they selectively inhibited only the growth of cancer cells, and had no effect on the growth of healthy cell lines. Both of the most active compounds contained a d-glucuronic acid unit. However, the anti-inflammatory effect of these derivatives has not yet been investigated.

As a continuation and completion of these studies, we produced seven additional trisaccharides containing a glucuronic acid unit. Of these, six derivatives are non-glycosaminoglycan analogues (Figure 2B, **9**–**15**) with different protective group patterns, and one is a glycosaminoglycan analogue trisaccharide (**15**). In our work, we continued to focus on the preparation of derivatives containing d-glucuronic acid, since the production of this building block is much simpler and more efficient than that of the l-iduronic acid unit, and thus, we obtained HS-like derivatives that might have heparanase inhibitory activity [31,32]. The synthesized molecules contain methyl, acetyl, hydroxyl, and/or sulphate ester groups in a varied composition, so we can examine the effect of the used groups on biological activity and, knowing the active structures, we have the opportunity to design further biologically effective molecules in a targeted manner.

In this publication, we describe the synthesis of six non-glycosaminoglycan analogues (Glc-GlcA-Glc-sequenced, **9**–**14**) and one heparin-related (GlcN-GlcA-Glc-sequenced, **15**) trisaccharide derivatives containing acetyl, sulphate, and methyl substituents and present the results of the performed biological tests (cell growth inhibitory effect, cytotoxicity, antioxidant and anti-inflammatory effects). Our aim was to investigate the effect of the functional group pattern found in the final products on their biological activity, completing/supplementing our previous research. Based on these tests, we can obtain a much more comprehensive picture of what kind of groups will be worth forming on the planned higher oligosaccharides with a larger number of members.

## 2. Materials and Methods

### 2.1. Synthesis

#### 2.1.1. General Methods

Optical rotations were measured at room temperature on a Perkin-Elmer 241 automatic polarimeter (PerkinElmer, Waltham, MA, USA). TLC analysis was performed on Kieselgel 60 F_254_ (Merck, Kenilworth, NJ, USA) silica gel plates with visualization by immersing in a sulphuric-acid solution (5% in EtOH, VWR International Ltd., Radnor, PA, USA) followed by heating. Column chromatography was performed on silica gel 60 (Merck 0.063–0.200 mm). Organic solutions were dried over MgSO_4_ and concentrated under a vacuum. ^1^H- and J-modulated ^13^C NMR spectroscopy (^1^H: 400 and 500 MHz; ^13^C: 100.28 and 125.76 MHz) were performed on Bruker DRX-400 and Bruker Avance II 500 spectrometers (Bruker, Billerica, MA, USA) at 25 °C. Chemical shifts are referenced to SiMe_4_ or sodium 3-(trimethylsilyl)-1-propanesulfonate (DSS, d = 0.00 ppm for ^1^H nuclei) and to residual solvent signals (CDCl_3_: *δ* = 77.16 ppm, CD_3_OD: *δ* = 49.15 ppm for ^13^C nuclei). The 1D and 2D NMR spectra of the synthesized compounds you can find the Supplementary Materials (Figures S1–S19). HRMS measurements were carried out on a maXis II UHR ESI-QTOF MS instrument (Bruker, Billerica, MA, USA) in positive ionization mode. The following parameters were applied for the electrospray ion source: capillary voltage: 3.6 kV; end plate offset: 500 V; nebulizer pressure: 0.5 bar; dry gas temperature: 200 °C; and dry gas flow rate: 4.0 L/min. Constant background correction was applied for each spectrum; the background was recorded before each sample by injecting the blank sample matrix (solvent). Na-formate calibrant was injected after each sample, which enabled internal calibration during data evaluation. Mass spectra were recorded by otofControl version 4.1 (build: 3.5, Bruker, Billerica, MA, USA) and processed by Compass DataAnalysis version 4.4 (build: 200.55.2969).

##### General Method A for Zemplén Deacetylation (**10** and **12**)

To a stirred solution of acetyl-containing derivatives (**9** [30] and **11**) (0.074 mmol) in MeOH (4.0 mL), NaOMe (25 mg) was added, and the reaction mixture was stirred for 24 h at room temperature. After 24 h, the mixture was neutralized with Amberlite IR 120 H^+^ ion exchange resin (**10**) or with 60% AcOH solution (3 drops) (**12**), filtered, washed with MeOH and concentrated.

##### General Method B for Cleavage of the Benzyl Groups by Catalytic Hydrogenation (**11** and **33**)

To a solution of the oxidized products (**28** and **32**) (0.556 mmol) in EtOH (27 mL), we added 20% Pd(OH)_2_-C (233 mg) and Et_3_SiH (1.15 mL, 7.225 mmol, 13.0 equiv.), and the reaction mixture was stirred at room temperature for 24 h. The reaction mixture was filtered through a pad of Celite, washed with MeOH and concentrated under reduced pressure.

##### General Method C for Introduction of the Sulphate Ester Groups (**13** and **15**)

SO_3_·Et_3_N complex (7.098 mmol, 5.0 equiv./–OH) was added to a solution of the appropriate trisaccharide polyols (**11** and **33**) (0.203 mmol) in dry DMF (10.9 mL). The reaction mixture was stirred at 50 °C for 24 h. Subsequently, the reaction was quenched with NaHCO_3_ solution (35.49 mmol, 5.0 equiv. to the complex), and the mixture was concentrated under reduced pressure.

##### General Method D for Deacetylation/Debenzoylation of the Sulphated Derivatives (**14** and **15**)

To a solution of the appropriate sulphated trisaccharides (**13** and sulphated **33**) (0.040 mmol) in MeOH (1.63 mL) a solution of NaOH (3 M, 871 µL) was added at 0 °C. The reaction mixture was allowed to warm up to room temperature and stirred for 24 h. Subsequently, the reaction mixture was neutralized with AcOH solution (60%), and the mixture was concentrated under reduced pressure.

##### General Method E for Glycosylation Reactions Using Thioglycoside Donor (**18**, **25** and **30**)

To a solution of the appropriate 4-OH acceptor (**17** [33] and **19**) (3.231 mmol) and monosaccharide donor (**16** [34], **24** and **29**) (4.846 mmol, 1.5 equiv.) in dry CH_2_Cl_2_ (75.0 mL), 4 Å MS (50 pieces) were added and the reaction mixture was stirred at room temperature under argon. After 30 min, the stirred mixture was cooled to −40 °C under argon. At this temperature, NIS (7.269 mmol, 1.5 equiv. to the donor) and TfOH (2.181 mmol, 0.3 equiv. to NIS) dissolved in dry THF (3.0 mL) were added. The temperature was allowed to warm up to −20 °C (**18** and **25**) or to −10 °C (**30**) and the reaction mixture was stirred for 4 h. Then, the reaction mixture was quenched with Et_3_N (500 µL), diluted with CH_2_Cl_2_ (500 mL), filtered and washed with a saturated aqueous solution of Na_2_S_2_O_3_ (2 × 100 mL) and a saturated aqueous solution of NaHCO_3_ (100 mL) and H_2_O (2 × 100 mL) until neutral pH was reached. The organic layer was dried over MgSO_4_ and concentrated.

##### General Method F for Regioselective Ring Opening Reaction of the 4,6-*O*-acetals (**19** and **26**)

The appropriate 4,6-*O*-acetals (**18** and **25**) (3.194 mmol) were dissolved in anhydrous CH_2_Cl_2_ (28 mL); then, 4 Å MS (~50 pieces) were added and the solution was stirred at room temperature for 30 min. After 30 min, the mixture was cooled to 0 °C; then, Et_3_SiH (6.12 mL, 38.33 mmol, 12.0 equiv.) and BF_3_·Et_2_O (788 μL, 6.388 mmol, 2.0 equiv.) were added. The reaction mixture was stirred at 0 °C for 5 h. Then, the mixture was diluted with CH_2_Cl_2_ (500 mL), washed with water (75 mL), saturated aqueous solution of NaHCO_3_ (2 × 75 mL), and water (2 × 75 mL) until a neutral pH was reached. The organic layer was dried over MgSO_4_ and concentrated.

##### General Method G for Cleavage of the (2-Naphthyl)methyl Group (**27** and **31**)

The appropriate trisaccharide derivatives (**26** and **30**) (1.265 mmol) were dissolved in a mixture of CH_2_Cl_2_ (21 mL) and water (2.1 mL); then, DDQ was added (1.898 mmol, 1.5 equiv.) and the reaction mixture was stirred for 30 min at room temperature. After 30 min, the reaction mixture was diluted with CH_2_Cl_2_ (400 mL) and washed with a saturated aqueous solution of NaHCO_3_ (2 × 50 mL) and water (2 × 50 mL) until a neutral pH was reached. The organic layer was dried over MgSO_4_ and concentrated under reduced pressure.

##### General Method H for Oxidation of the C-6-OH to Carboxylic Acid (**28** and **32**)

The appropriate 6-OH derivatives (**27** and **31**) (0.887 mmol) were dissolved in CH_3_CN (34 mL) and H_2_O (12 mL); then, BAIB (857 mg, 2.660 mmol, 3.0 equiv./−OH) and TEMPO (25 mg, 0.160 mmol, 0.18 equiv./OH) were added at 0 °C. The solution was stirred vigorously for 24 h at room temperature. After 24 h, the reaction mixture was quenched by the addition of 10% aqueous solution of Na_2_S_2_O_3_ (44 mL) and extracted with CH_2_Cl_2_ (3 × 100 mL), and the combined organic layers were dried over MgSO_4_, and concentrated.

#### 2.1.2. Sodium [methyl (2,3,4-tri-O-methyl-α-d-glucopyranosyl)-(1→4)-(β-d-glucopyranosyl-uronate)-(1→4)-α-d-glucopyranoside] (**10**)

Compound **9** [30] (50 mg, 0.074 mmol) was deacetylated according to general method A. The crude product was purified by silica gel chromatography (1:1 CH_2_Cl_2_/MeOH) to give **10** (39 mg, 89%) as a colourless syrup. [α]_D_^25^ +114.6 (*c* 0.24, MeOH); *R*_f_ 0.37 (1:1 CH_2_Cl_2_/MeOH); ^1^H NMR (D_2_O, 500 MHz) *δ* 5.63 (d, *J* = 3.2 Hz, 1H, H-1″), 4.74 (d, *J* = 3.4 Hz, 1H, H-1), 4.52 (d, *J* = 7.9 Hz, 1H, H-1′), 4.04 (d, *J* = 9.7 Hz, 1H, H-5′), 3.87–3.77 (m, 3H, H-4′, H-6″a, H-6″b), 3.74–3.69 (m, 5H, H-3′, H-5, H-5″, H-6a,b), 3.56–3.52 (m, 5H, H-2, H-4″, OC*H*_3_), 3.49, 3.47 (2 × s, 6H, 2 × OC*H*_3_), 3.45–3.41 (m, 2H, H-3, H-3″), 3.36 (s, 3H, C-1-OC*H*_3_), 3.35–3.31 (m, 1H, H-2′), 3.29–3.23 (m, 2H, H-2″, H-4) ppm; ^13^C NMR (D_2_O, 125 MHz) *δ* 172.2 (1C, C_q_, COOH), 102.3 (1C, C-1′), 99.0 (1C, C-1), 95.8 (1C, C-1″), 81.5 (1C, C-3″), 80.2 (1C, C-2″), 78.6 (1C, C-4″), 78.2 (1C, C-4), 76.0 (1C, C-3′), 75.2 (1C, C-4′), 73.8 (1C, C-5′), 73.2 (1C, C-2′), 71.5 (1C, C-5″), 71.1 (1C, C-3), 71.0 (1C, C-2), 70.3 (1C, C-5), 60.1 (1C, O*C*H_3_), 59.8 (1C, C-6″), 59.7 (1C, O*C*H_3_), 59.5 (1C, C-6), 58.1 (1C, O*C*H_3_), 55.1 (1C, C-1-O*C*H_3_) ppm; UHR ESI-QTOF (positive ion): *m*/*z* calcd for [C_22_H_38_O_17_ + Na]^+^: 597.2001; found: 597.2002.

#### 2.1.3. Sodium [methyl (α-d-glucopyranosyl)-(1→4)-(2,3-di-O-acetyl-β-d-glucopyranosyl-uronate)-(1→4)-α-d-glucopyranoside] (**11**)

Compound **28** (655 mg, 0.556 mmol) was debenzylated according to general method B. The crude product was purified by silica gel chromatography (4:6 CH_2_Cl_2_/MeOH) to give **11** (290 mg, 82%) as a colourless syrup. [α]_D_^25^ +92.1 (*c* 0.14, MeOH); *R*_f_ 0.43 (4:6 CH_2_Cl_2_/MeOH); ^1^H NMR (D_2_O, 400 MHz) *δ* 5.22 (t, *J* = 9.0 Hz, 1H, H-3′), 5.03 (d, *J* = 3.9 Hz, 1H, H-1″), 4.81–4.74 (m, 2H, H-1′, H-2′), 4.67 (d, *J* = 3.7 Hz, 1H, H-1), 3.97 (t, *J* = 9.4 Hz, 1H, H-4′), 3.84 (d, *J* = 9.7 Hz, 1H, H-5′), 3.72 (d, *J* = 11.0 Hz, 1H, H-6a), 3.66–3.59 (m, 5H, H-3, H-5″, H-6b, H-6″a,b), 3.54–3.52 (m, 1H, H-5), 3.49–3.44 (m, 3H, H-2, H-3″, H-4), 3.33–3.28 (m, 2H, H-2″, H-4″), 3.27 (s, 3H, C-1-OC*H*_3_), 1.99 (s, 6H, 2 × Ac-C*H*_3_) ppm; ^13^C NMR (D_2_O, 100 MHz) *δ* 174.5 (1C, C_q_ COOH), 173.7, 169.4 (2C, 2 × C_q_ Ac), 100.3 (1C, C-1′), 100.1 (1C, C-1″), 99.5 (1C, C-1), 79.5 (1C, C-4), 76.7 (1C, C-5′), 75.9 (1C, C-3′), 75.7 (1C, C-4′), 73.4 (1C, C-3″), 73.1 (1C, C-2′), 72.4 (1C, C-5″), 71.9 (1C, C-3), 71.7 (1C, C-2″), 71.5 (1C, C-2), 70.7 (1C, C-5), 69.6 (1C, C-4″), 60.5, 60.4 (2C, C-6, C-6″), 55.6 (1C, C-1-O*C*H_3_), 21.1, 20.7 (2C, 2 × Ac-*C*H_3_) ppm; UHR ESI-QTOF (positive ion): *m*/*z* calcd for [C_23_H_35_NaO_19_ + Na]^+^: 661.1562; found: 661.1559.

#### 2.1.4. Sodium [methyl (α-d-glucopyranosyl)-(1→4)-(β-d-glucopyranosyl-uronate)-(1→4)-α-d-glucopyranoside] (**12**)

Compound **11** (70 mg, 0.110 mmol) was deacetylated according to general method A. The crude product was purified by a Sephadex G-25 column in H_2_O to give **12** (54 mg, 89%) as a colourless syrup. [α]_D_^25^ +51.8 (*c* 0.11, H_2_O); *R*_f_ 0.57 (7:6:1 CH_2_Cl_2_/MeOH/H_2_O); ^1^H NMR (D_2_O, 400 MHz) *δ* 5.36 (d, *J* = 3.8 Hz, 1H, H-1″), 4.68 (d, *J* = 4.1 Hz, 1H, H-1), 4.39 (d, *J* = 7.9 Hz, 1H, H-1′), 3.81–3.78 (m, 1H, H-6a), 3.73–3.67 (m, 2H, H-5′, H-6b), 3.66–3.62 (m, 6H, H-3, H-3′, H-4, H-5, H-6″a,b), 3.60–3.54 (m, 2H, H-3″, H-5″), 3.50–3.45 (m, 2H, H-2, H-4), 3.39 (dd, *J* = 3.9 Hz, *J* = 9.9 Hz, 1H, H-2″), 3.29 (s, 3H, C-1-OC*H*_3_), 3.28–3.24 (m, 2H, H-2′, H-4″) ppm; ^13^C NMR (D_2_O, 100 MHz) *δ* 174.9 (1C, C_q_ COOH), 102.2 (1C, C-1′), 98.9 (1C, C-1), 98.2 (1C, C-1″), 78.5 (1C, C-4), 76.3 (2C, C-3′, C-5′), 76.1 (1C, C-4′), 73.1 (1C, C-2′), 72.7 (1C, C-5″), 71.8 (1C, C-3″), 71.6 (1C, C-2″), 71.5 (1C, C-3), 70.9 (1C, C-2), 70.2 (1C, C-5), 69.2 (1C, C-4″), 60.1, 59.8 (2C, C-6, C-6″), 55.1 (1C, C-1-O*C*H_3_) ppm; UHR ESI-QTOF (positive ion): *m*/*z* calcd for [C_19_H_31_NaO_17_ + Na]^+^: 577.1351; found: 577.1351.

#### 2.1.5. Octa Sodium [methyl (2,3,4,6-tetra-O-sulfonato-α-d-glucopyranosyl)-(1→4)-(2,3-di-O-acetyl-β-d-glucopyranosyl-uronate)-(1→4)-2,3,6-tri-O-sulfonato-α-d-glucopyranoside] (**13**)

Compound **11** (129 mg, 0.203 mmol) was sulphated according to general method C. The crude product was purified by Sephadex LH-20 gel chromatography in H_2_O and transformed to sodium salt using Dowex Na^+^ ion exchange resin to give **13** (222 mg, 81%) as a white solid. [α]_D_^25^ +40.0 (*c* 0.16, H_2_O); *R*_f_ 0.33 (7:6:1 CH_2_Cl_2_/MeOH/H_2_O); ^1^H NMR (D_2_O, 400 MHz) *δ* 5.39 (d, *J* = 3.4 Hz, 1H, H-1″), 5.31 (t, *J* = 8.5 Hz, 1H, H-3′), 5.09 (d, *J* = 3.6 Hz, 1H, H-1), 4.91–4.86 (m, 2H, H-1′, H-2′), 4.58–4.50 (m, 2H, H-3, H-3″), 4.39–4.29 (m, 6H, H-2, H-4″, H-6a,b, H-6″a,b), 4.25–4.17 (m, 2H, H-2″, H-4′), 4.04–4.01 (m, 1H, H-5″), 3.98–3.89 (m, 3H, H-4, H-5, H-5′), 3.39 (s, 3H, C-1-OC*H*_3_), 2.07, 2.06 (2 × s, 6H, 2 × Ac-C*H*_3_) ppm; ^13^C NMR (D_2_O, 100 MHz) *δ* 174.0 (1C, C_q_ COOH), 173.4, 173.0 (2C, 2 × C_q_ Ac), 98.7 (1C, C-1′), 97.1 (1C, C-1), 96.2 (1C, C-1″), 77.0 (1C, C-5′), 75.7 (1C, C-3), 75.3 (1C, C-2), 75.1 (1C, C-3″), 75.0 (1C, C-3″), 74.4 (1C, C-4′), 74.2 (1C, C-2″), 73.9 (1C, C-4), 73.6 (1C, C-4″), 72.7 (1C, C-2″), 68.9 (1C, C-5″), 68.7 (1C, C-5), 65.9 (1C, C-6″), 65.8 (1C, C-6), 55.4 (1C, C-1-O*C*H_3_), 21.0, 20.3 (2C, 2 × Ac-*C*H_3_) ppm; UHR ESI-QTOF (positive ion): *m*/*z* calcd for [C_23_H_28_Na_8_O_40_S_7_ + Na_2_]^2+^: 698.8584; found: 698.8583.

#### 2.1.6. Octa Sodium [methyl (2,3,4,6-tetra-O-sulfonato-α-d-glucopyranosyl)-(1→4)-(β-d-glucopyranosyl-uronate)-(1→4)-2,3,6-tri-O-sulfonato-α-d-glucopyranoside] (**14**)

Compound **13** (54 mg, 0.040 mmol) was deacetylated according to general method D. The crude product was purified by Sephadex LH-20 gel chromatography in H_2_O and transformed to sodium salt using Dowex Na^+^ ion exchange resin to give **14** (45 mg, 89%) as a white solid. [α]_D_^25^ +120.0 (*c* 0.02, H_2_O); *R*_f_ 0.45 (75:25 MeCN/H_2_O); ^1^H NMR (D_2_O, 400 MHz) *δ* 5.67 (d, *J* = 3.6 Hz, 1H, H-1″), 5.04 (d, *J* = 3.6 Hz, 1H, H-1), 4.57–4.50 (m, 3H, H-1′, H-3, H-3″), 4.34–4.30 (m, 1H, H-4″), 4.28–4.24 (m, 5H, H-2, H-2″, H-6a, H-6″a,b), 4.19 (dd, *J* = 2.3 Hz, *J* = 10.9 Hz, 1H, H-6b), 3.99–3.91 (m, 3H, H-4, H-5, H-5″), 3.80–3.71 (m, 3H, H-3′, H-4′, H-5′), 3.35 (s, 3H, C-1-OC*H*_3_), 3.34–3.29 (m, 1H, H-2′) ppm; ^13^C NMR (D_2_O, 100 MHz) *δ* 174.9 (1C, C_q_ COOH), 100.7 (1C, C-1′), 97.0 (1C, C-1), 96.1 (1C, C-1″), 77.0 (1C, C-4′), 76.9 (1C, C-3′), 76.2 (1C, C-3), 76.0 (1C, C-5′), 75.3 (1C, C-3″), 75.1 (1C, C-2″), 74.8 (1C, C-2), 73.6 (1C, C-4″), 73.0 (1C, C-2′), 72.8 (1C, C-4), 68.8 (1C, C-5), 68.5 (1C, C-5″), 66.3 (1C, C-6″), 65.9 (1C, C-6), 55.3 (1C, C-1-O*C*H_3_) ppm; UHR ESI-QTOF (positive ion): *m*/*z* calcd for [C_19_H_24_Na_8_O_38_S_7_ + Na_2_]^2+^: 656.8478; found: 656.8474.

#### 2.1.7. Hexa Sodium [methyl (2-deoxy-2-sulfamido-6-O-sulfonato-α-d-glucopyranosyl)-(1→4)-(β-d-glucopyranosyl-uronate)-(1→4)-2,3,6-tri-O-sulfonato-α-d-glucopyranoside] (**15**)

Compound **33** (55 mg, 0.053 mmol) was sulphated according to general method C. The product was separated from the salt contaminants by Sephadex LH-20 gel chromatography in H_2_O, and the sulphated compound was debenzoylated according to general method D. The crude product was purified by Sephadex LH-20 gel chromatography in H_2_O and transformed to sodium salt using Dowex Na^+^ ion exchange resin to give **15** (25 mg, 45% for two steps) as a white solid. [α]_D_^25^ +51.8 (*c* 0.11, H_2_O); *R*_f_ 0.34 (8:2 CH_3_CN/H_2_O); ^1^H NMR (D_2_O, 500 MHz) *δ* 5.57 (d, *J* = 3.8 Hz, 1H, H-1″), 5.09 (d, *J* = 3.7 Hz, 1H, H-1), 4.60 (d, *J* = 8.0 Hz, 1H, H-1′), 4.57 (t, *J* = 9.4 Hz, 1H, H-3), 4.32–4.29 (m, 4H, H-2, H-6″a, H-6a,b), 4.10 (dd, *J* = 2.0 Hz, *J* = 11.1 Hz, 1H, H-6″b), 4.00–3.97 (m, 2H, H-5, H-4), 3.83–3.81 (m, 1H, H-5″), 3.79–3.75 (m, 2H, H-3′, H-5′), 3.73–3.71 (m, 1H, H-4′), 3.57–3.50 (m, 2H, H-3″, H-4″), 3.40 (s, 3H, C-3′-OC*H*_3_) 3.38–3.35 (m, 1H, H-2′), 3.20 (dd, *J* = 3.8 Hz, *J* = 10.0 Hz, 1H, H-2″) ppm; ^13^C NMR (D_2_O, 125 MHz) *δ* 175.1 (1C, C=O, COOH), 100.6 (1C, C-1′), 97.4 (1C, C-1″), 97.1 (1C, C-1), 77.0 (1C, C-4′), 76.5 (1C, C-3), 76.3 (1C, C-5′), 76.2 (1C, C-3′), 75.1 (1C, C-2), 72.8 (1C, C-4), 72.5 (1C, C-2′), 71.1 (1C, C-4″), 69.7 (1C, C-3′), 69.0 (1C, C-5″), 68.8 (1C, C-5), 66.3 (2C, C-6, C-6″), 57.9 (1C, C-2″), 55.3 (1C, C-1-O*C*H_3_) ppm; UHR ESI-QTOF (positive ion): *m*/*z* calcd for [C_19_H_27_NNa_6_O_31_S_5_ + Na_2_]^2+^: 554.4171; found: 554.4172.

#### 2.1.8. Methyl [2,3-di-O-acetyl-4,6-O-(2-naphthyl)methylene-β-d-glucopyranosyl]-(1→4)-2,3,6-tri-O-benzyl-α-d-glucopyranoside (**18**)

Compound **17** [33] (1.50 g, 3.231 mmol) was glycosylated with **16** [34] (2.40 g, 4.846 mmol, 1.5 equiv.) according to general method E. The crude product was purified by silica gel chromatography (6:4 *n*-hexane/EtOAc) to give **18** (2.18 g, 80%) as a colourless syrup. [α]_D_^25^ +33.7 (*c* 0.32, CHCl_3_); *R*_f_ 0.39 (6:4 *n*-hexane/EtOAc); ^1^H NMR (CDCl_3_, 500 MHz) *δ* 7.74–7.19 (m, 22H, arom.), 5.53 (s, 1H, H_ac_), 5.12 (t, *J* = 9.4 Hz, 1H, H-3′), 4.92–4.89 (m, 2H, Bn-C*H*_2a_, H-2′), 4.82 (d, *J* = 10.9 Hz, 1H, Bn-C*H*_2b_), 4.79–4.75 (m, 2H, 2 × Bn-C*H*_2a_), 4.62 (d, *J* = 12.3 Hz, 1H, Bn-C*H*_2b_), 4.60 (d, *J* = 3.6 Hz, 1H, H-1), 4.54 (d, *J* = 7.9 Hz, 1H, H-1′), 4.42 (d, *J* = 12.0 Hz, 1H, Bn-C*H*_2b_), 4.17 (dd, *J* = 4.9 Hz, *J* = 10.5 Hz, 1H, H-6′a), 3.91 (t, *J* = 9.4 Hz, 1H, H-4), 3.83 (t, *J* = 9.2 Hz, 1H, H-3), 3.79 (dd, *J* = 2.8 Hz, *J* = 10.7 Hz, 1H, H-6a), 3.63–3.58 (m, 3H, H-4′, H-5, H-6b), 3.51 (dd, *J* = 3.6 Hz, *J* = 9.5 Hz, 1H, H-2), 3.45 (t, *J* = 10.3 Hz, 1H, H-6′b), 3.37 (s, 3H, C-1-OC*H*_3_), 3.20 (td, *J* = 4.9 Hz, *J* = 9.7 Hz, 1H, H-5′), 2.01, 1.96 (2 × s, 6H, 2 × Ac-C*H*_3_) ppm; ^13^C NMR (CDCl_3_, 125 MHz) *δ* 170.1, 169.4 (2C, 2 × C_q_ Ac), 139.4, 138.3, 137.6, 134.3, 133.7, 132.8 (6C, 6 × C_q_ arom.), 128.8–123.7 (22C, arom.), 101.6 (1C, C_ac_), 100.5 (1C, C-1′), 98.4 (1C, C-1), 79.9 (1C, C-3), 79.0 (1C, C-2), 78.4 (1C, C-4′), 77.1 (1C, C-4), 75.3, 73.7, 73.6 (3C, 3 × Bn-*C*H_2_), 73.1 (1C, C-2′), 72.2 (1C, C-3′), 69.8 (1C, C-5), 68.6 (1C, C-6′), 67.4 (1C, C-6), 66.0 (1C, C-5′), 55.4 (1C, C-1-O*C*H_3_), 20.8, 20.7 (2C, 2 × Ac-*C*H_3_) ppm; UHR ESI-QTOF (positive ion): *m*/*z* calcd for [C_49_H_52_O_13_ + Na]^+^: 871.3300; found: 871.3300.

#### 2.1.9. Methyl [2,3-di-O-acetyl-6-O-(2-naphthyl)methyl-β-d-glucopyranosyl]-(1→4)-2,3,6-tri-O-benzyl-α-d-glucopyranoside (**19**), and Methyl [2,3-di-O-acetyl-β-d-glucopyranosyl]-(1→4)-2,3,6-tri-O-benzyl-α-d-glucopyranoside (**20**) and Methyl [2,3-di-O-acetyl-4-O-(2-naphthyl)methyl-β-d-glucopyranosyl]-(1→4)-2,3,6-tri-O-benzyl-α-d-glucopyranoside (**21**)

Reaction 1: Compound **18** (2.71 g, 3.194 mmol) was converted to **19** by general method F. The crude product was purified by silica gel chromatography (55:45 hexane/EtOAc, then 6:4 hexane/acetone) to give compound **19** (1.77 g, 65%) as a white foam and compound **20** (600 mg, 26%) as a white foam.

Reaction 2: To a solution of compound **18** (1.56 g, 1.839 mmol) in dry THF (5.6 mL), 4 Å MS and Me_3_N·BH_3_ (805 mg, 11.033 mmol, 6.0 equiv.) were added, and the reaction mixture was stirred for 30 min at room temperature. After 30 min, the reaction mixture was cooled to 0 °C, AlCl_3_ (1.47 g, 11.033 mmol, 6.0 equiv.) was added, and the mixture was stirred at room temperature for 3 h. After 3 h, the reaction mixture was diluted with CH_2_Cl_2_ (50 mL) and washed with H_2_O (2 × 10 mL). The organic layer was dried over MgSO_4_, filtered and concentrated. The crude product was purified by silica gel chromatography (65:35 *n*-hexane/EtOAc) to give compound **19** (1.19 g, 76%) as a colourless syrup and compound **21** (116 mg, 7.5%) as a colourless syrup.

Data of **19**: [α]_D_^25^ −4.7 (*c* 0.17, CHCl_3_); *R*_f_ 0.40 (6:4 *n*-hexane/acetone); ^1^H NMR (CDCl_3_, 400 MHz) *δ* 7.83–7.15 (m, 22H, arom.), 4.93–4.70 (m, 6H, 3 × Bn-C*H*_2a_, NAP-C*H*_2a_, H-2′, H-3′), 4.59–4.41 (m, 6H, 3 × Bn-C*H*_2a_, NAP-C*H*_2a_, H-1, H-1′), 3.88–3.79 (m, 2H, H-3, H-4), 3.75 (dd, *J* = 2.5 Hz, *J* = 10.4 Hz, 1H, H-6a), 3.67 (t, *J* = 9.0 Hz, 1H, H-4′), 3.63–3.54 (m, 3H, H-5, H-6b, H-6′a), 3.49–3.44 (m, 2H, H-2, H-6′b), 3.35 (s, 3H, C-1-OC*H*_3_), 3.26 (dt, *J* = 5.3 Hz, *J* = 10.0 Hz, 1H, H-5′), 3.18 (bs, 1H, H-4′-O*H*), 2.05, 1.94 (2 × s, 6H, 2 × Ac-C*H*_3_) ppm; ^13^C NMR (CDCl_3_, 100 MHz) *δ* 171.0, 169.5 (2C, 2 × C_q_ Ac), 139.5, 138.3, 137.7, 135.0, 133.3, 133.1 (6C, 6 × C_q_ arom.), 128.7–125.6 (22C, arom.), 100.1 (1C, C-1′), 98.4 (1C, C-1), 80.9 (1C, C-4), 79.1 (1C, C-2), 77.0 (1C, C-3), 75.8 (1C, C-3′), 75.2, 73.9, 73.8, 73.6 (4C, 3 × Bn-*C*H_2_, NAP-*C*H_2_), 73.2 (1C, C-5′), 72.2 (1C, C-2′), 71.8 (1C, C-4′), 70.9 (1C, C-6′), 69.9 (1C, C-5), 67.7 (1C, C-6), 55.4 (1C, C-1-O*C*H_3_), 20.9, 20.8 (2C, 2 × Ac-*C*H_3_) ppm; UHR ESI-QTOF (positive ion): *m*/*z* calcd for [C_49_H_54_O_13_ + Na]^+^: 873.3457; found: 873.3457.

Data of **20**: [α]_D_^25^ +10.0 (*c* 0.10, CHCl_3_); *R*_f_ 0.14 (55:45 *n*-hexane/EtOAc); ^1^H NMR (CDCl_3_, 500 MHz) *δ* 7.41–7.26 (m, 15H, arom.), 4.87–4.73 (m, 6H, 2 × Bn-C*H*_2a_, Bn-C*H*_2_, H-2′, H-3′), 4.65 (d, *J* = 12.2 Hz, 1H, Bn-C*H*_2b_), 4.59 (d, *J* = 3.5 Hz, 1H, H-1), 4.42–4.38 (m, 2H, H-1′, Bn-C*H*_2b_), 3.82–3.80 (m, 2H, H-3, H-4), 3.73 (dd, *J* = 2.4 Hz, *J* = 10.5 Hz, 1H, H-6a), 3.62–3.60 (m, 3H, H-5, H-6b, H-6′a), 3.53 (td, *J* = 4.7 Hz, *J* = 9.3 Hz, 1H, H-4′), 3.49 (dd, *J* = 3.9 Hz, *J* = 8.9 Hz, 1H, H-2), 3.37 (s, 3H, C-1-OC*H*_3_), 3.33 (dd, *J* = 4.7 Hz, *J* = 11.7 Hz, 1H, H-6′b), 3.04 (dq, *J* = 3.1 Hz, *J* = 8.8 Hz, 1H, H-5′), 2.59 (d, *J* = 4.8 Hz, 1H, H-4′-O*H*), 2.07, 1.95 (2 × s, 6H, 2 × Ac-C*H*_3_), 1.70 (bs, 1H, H-6′-O*H*) ppm; ^13^C NMR (CDCl_3_, 125 MHz) *δ* 171.0, 169.4 (2C, 2 × C_q_ Ac), 139.4, 138.3, 137.6 (3C, 3 × C_q_ arom.), 128.8–127.2 (15C, arom.), 99.9 (1C, C-1′), 98.6 (1C, C-1), 79.9 (1C, C-4), 79.0 (1C, C-2), 77.0 (1C, C-3), 76.7 (1C, C-3′), 75.4 (1C, C-5′), 75.3, 73.9, 73.7 (3C, 3 × Bn-*C*H_2_), 72.0 (1C, C-2′), 69.9 (1C, C-5), 69.7 (1C, C-4′), 67.5 (1C, C-6), 61.9 (1C, C-6′), 55.5 (1C, C-1-O*C*H_3_), 21.0, 20.9 (2C, 2 × Ac-*C*H_3_) ppm; UHR ESI-QTOF (positive ion): *m*/*z* calcd for [C_38_H_46_O_13_ + Na]^+^: 733.2831; found: 733.2829.

Data of **21**: [α]_D_^25^ +6.0 (*c* 0.10, CHCl_3_); *R*_f_ 0.45 (1:1 *n*-hexane/acetone); ^1^H NMR (CDCl_3_, 500 MHz) *δ* 7.84–7.16 (m, 22H, arom.), 4.93–4.43 (m, 11H, 3 × Bn-C*H*_2_, NAP-C*H*_2_, H-2′, H-1, H-1′), 3.85–3.82 (m, 2H, H-3, H-4), 3.79 (dd, *J* = 2.8 Hz, *J* = 10.7 Hz, 1H, H-6a), 3.64–3.59 (m, 2H, H-5, H-6b), 3.57–3.53 (m, 2H, H-4′, H-6′a), 3.49–3.44 (m, 2H, H-2, H-6′b), 3.39 (dd, *J* = 3.4 Hz, *J* = 9.4 Hz, 1H, H-3′), 3.36 (s, 3H, C-1-OC*H*_3_), 3.24 (dt, *J* = 5.6 Hz, *J* = 10.7 Hz, 1H, H-5′), 3.18 (bs, 1H, H-4′-O*H*), 2.57 (bs, 1H, H-3′-O*H*), 2.03 (s, 3H, Ac-C*H*_3_) ppm; ^13^C NMR (CDCl_3_, 125 MHz) *δ* 170.7 (1C, C_q_ Ac), 139.6, 138.4, 137.9, 135.1, 133.4, 133.2 (6C, 6 × C_q_ arom.), 128.7–125.6 (22C, arom.), 100.2 (1C, C-1′), 98.5 (1C, C-1), 80.1 (1C, C-4), 79.2 (1C, C-2), 77.2 (1C, C-3), 75.6 (1C, C-3′), 75.3 (1C, Bn-*C*H_2_), 74.5 (1C, C-2′), 74.0 (1C, C-4′), 73.9, 73.8, 73.7 (3C, 2 × Bn-*C*H_2_, NAP-*C*H_2_), 72.9 (1C, C-5′), 71.1 (1C, C-6′), 70.0 (1C, C-5), 67.9 (1C, C-6), 55.5 (1C, C-1-O*C*H_3_), 21.2 (1C, Ac-*C*H_3_) ppm; UHR ESI-QTOF (positive ion): *m*/*z* calcd for [C_47_H_52_O_12_ + Na]^+^: 831.3351; found: 831.3347.

#### 2.1.10. Methyl (2,3-di-O-benzyl-4,6-O-benzylidene-α-d-glucopyranosyl)-(1→4)-[2,3-di-O-acetyl-6-O-(2-naphthyl)methyl-β-d-glucopyranosyl]-(1→4)-2,3,6-tri-O-benzyl-α-d-glucopyranoside (**25**)

Compound **19** (2.60 g, 3.055 mmol) was glycosylated with **24** (2.48 g, 4.582 mmol, 1.5 equiv.) according to general method E. The crude product was purified by silica gel chromatography (6:4 then 1:1 *n*-hexane/EtOAc) to give **25** (2.40 g, 61%) as a light yellow foam and **19** (764 mg, 20%) starting material as a light yellow foam. [α]_D_^25^ +2.5 (*c* 0.12, CHCl_3_); *R*_f_ 0.37 (65:35 *n*-hexane/acetone); ^1^H NMR (CDCl_3_, 400 MHz) *δ* 7.78–7.14 (m, 37H, arom.), 5.47 (1H, H_Ac_), 5.15 (t, *J* = 9.2 Hz, 1H, H-3′), 5.08–4.45 (m, 16H, 5 × Bn-C*H*_2_, NAP-C*H*_2_, H-1, H-1′, H-1″, H-2′), 4.11–4.03 (m, 2H, H-4′, H-6″a), 3.91–3.78 (m, 3H, H-3, H-3″, H-4), 3.84–3.77 (m, 2H, H-5″, H-6a), 3.69–3.60 (m, 4H, H-5, H-6a,b’, H-6b), 3.49–3.43 (m, 3H, H-2, H-4″, H-6″b), 3.35 (s, 3H, C-1-OC*H*_3_), 3.36–3.33 (m, 1H, H-2″), 3.13–3.11 (m, 1H, H-5′), 1.96, 1.87 (2 × s, 6H, 2 × Ac-C*H*_3_) ppm; ^13^C NMR (CDCl_3_, 100 MHz) *δ* 171.2, 169.6 (2C, 2 × C_q_ Ac), 139.6, 138.7, 138.3, 137.9, 137.7, 137.5, 135.7, 133.3, 132.9 (9C, 9 × C_q_ arom.), 128.9–125.8 (37C, arom.), 101.3 (1C, C_ac_), 100.1 (1C, C-1′), 98.4 (1C, C-1), 98.3 (1C, C-1″), 82.1 (1C, C-4″), 80.3 (1C, C-4), 79.3 (1C, C-2), 79.2 (1C, C-2″), 78.1 (1C, C-3″), 77.1 (1C, C-3), 75.4, 75.2 (2C, 2 × Bn-*C*H_2_), 75.0 (1C, C-5′), 74.7 (2C, C-3′, C-4′), 73.7, 73.6, 73.5, 73.4 (4C, 3 × Bn-*C*H_2_, NAP-*C*H_2_), 72.7 (1C, C-2′), 69.8 (1C, C-5), 68.9 (1C, C-6″), 68.1 (1C, C-6), 67.9 (1C, C-6′), 63.5 (1C, C-5″), 55.4 (1C, C-1-O*C*H_3_), 21.0, 20.9 (2C, 2 × Ac-*C*H_3_) ppm; UHR ESI-QTOF (positive ion): *m*/*z* calcd for [C_76_H_80_O_18_ + Na]^+^: 1303.5237; found: 1303.5237.

#### 2.1.11. Methyl (2,3,6-tri-O-benzyl-α-d-glucopyranosyl)-(1→4)-[2,3-di-O-acetyl-6-O-(2-naphthyl)methyl-β-d-glucopyranosyl]-(1→4)-2,3,6-tri-O-benzyl-α-d-glucopyranoside (**26**)

Compound **25** (506 mg, 0.395 mmol) was converted to **26** by general method F. The crude product was purified by silica gel chromatography (6:4 *n*-hexane/EtOAc, then 1:1 *n*-hexane/acetone) to give **26** (381 mg, 75%) as a white foam. [α]_D_^25^ +13.1 (*c* 0.13, CHCl_3_); *R*_f_ 0.40 (6:4 *n*-hexane/acetone); ^1^H NMR (CDCl_3_, 400 MHz) *δ* 7.78–7.12 (m, 37H, arom.), 5.15 (t, *J* = 9.3 Hz, 1H, H-3′), 5.08–4.34 (m, 18H, 6 × Bn-C*H*_2_, NAP-C*H*_2_, H-1, H-1′, H-1″, H-2′), 4.01 (t, *J* = 9.3 Hz,1H, H-4′), 3.89–3.86 (m, 2H, H-3, H-4), 3.83–3.72 (m, 3H, H-5″, H-6a, H-6′a), 3.70–3.61 (m, 4H, H-3″, H-5, H-6b, H-6′b), 3.49–3.47 (m, 4H, H-2, H-4″, H-6″a,b), 3.35 (s, 3H, C-1-OC*H*_3_), 3.35–3.32 (m, 1H, H-2″), 3.16–3.13 (m, 1H, H-5′), 2.26 (s, 1H, H-4″-OH), 1.96, 1.87 (2 × s, 6H, 2 × Ac-C*H*_3_) ppm; ^13^C NMR (CDCl_3_, 100 MHz) *δ* 171.4, 169.5 (2C, 2 × C_q_ Ac), 139.6, 138.8, 138.3, 138.0, 137.8, 137.7, 136.0, 133.3, 132.9 (9C, 9 × C_q_ arom.), 128.7–125.8 (37C, arom.), 100.1 (1C, C-1′), 98.4 (1C, C-1), 97.9 (1C, C-1″), 80.5 (1C, C-3″), 80.2 (1C, C-4), 79.8 (1C, C-2″), 79.2 (1C, C-2), 77.1 (1C, C-3), 75.4 (2C, C-4′, C-5′), 75.3, (2C, 2 × Bn-*C*H_2_), 74.6 (1C, C-3′), 73.7, 73.6, 73.5, 73.2, 73.0 (5C, 4 × Bn-*C*H_2_, NAP-*C*H_2_), 72.7 (1C, C-2′), 71.2 (1C, C-5″), 70.7 (1C, C-4″), 69.9 (1C, C-5), 69.6 (1C, C-6″), 68.4 (1C, C-6), 67.9 (1C, C-6′), 55.4 (1C, C-1-O*C*H_3_), 21.1, 20.9 (2C, 2 × Ac-*C*H_3_) ppm; UHR ESI-QTOF (positive ion): *m*/*z* calcd for [C_76_H_82_O_18_ + Na]^+^: 1305.5393; found: 1305.5393.

#### 2.1.12. Methyl (2,3,6-tri-O-benzyl-α-d-glucopyranosyl)-(1→4)-(2,3-di-O-acetyl-β-d-glucopyranosyl)-(1→4)-2,3,6-tri-O-benzyl-α-d-glucopyranoside (**27**)

Compound **26** (1.622 g, 1.265 mmol) was converted to **27** by general method G. The crude product was purified by silica gel chromatography (9:1 CH_2_Cl_2_/acetone) to give **27** (1.125 g, 75%) as a colourless syrup. [α]_D_^25^ +8.6 (*c* 0.22, CHCl_3_); *R*_f_ 0.38 (9:1 CH_2_Cl_2_/acetone); ^1^H NMR (CDCl_3_, 400 MHz) *δ* 7.40–7.23 (m, 30H, arom.), 5.12 (t, *J* = 9.3 Hz, 1H, H-3′), 4.92–4.39 (m, 16H, 6 × Bn-C*H*_2_, H-1, H-1′, H-1″, H-2′), 3.82–3.40 (m, 15H, skeleton protons), 3.37 (s, 3H, C-1-OC*H*_3_), 3.06–3.03 (m, 1H, H-5′), 2.28 (s, 1H, H-4″-OH), 1.95, 1.88 (2 × s, 6H, 2 × Ac-C*H*_3_), 1.71–1.65 (m, 1H, H-6-OH) ppm; ^13^C NMR (CDCl_3_, 100 MHz) *δ* 170.3, 169.6 (2C, 2 × C_q_ Ac), 139.4, 138.8, 138.4, 138.0, 137.9, 137.6 (6C, 6 × C_q_ arom.), 128.8–127.2 (30C, arom.), 99.9 (1C, C-1′), 98.6 (1C, C-1), 98.1 (1C, C-1″), 80.7, 80.0, 79.8, 79.0, 77.1, 75.3, 75.2, 74.7, 72.7, 71.5, 70.6, 69.9 (12C skeleton carbons), 75.4, 73.9, 73.7, 73.4 (6C, 6 × Bn-*C*H_2_), 69.5 (1C, C-6″), 67.6 (1C, C-6), 61.5 (1C, C-6′), 55.5 (1C, C-1-O*C*H_3_), 21.1, 20.9 (2C, 2 × Ac-*C*H_3_) ppm; UHR ESI-QTOF (positive ion): *m*/*z* calcd for [C_65_H_74_O_18_ + Na]^+^: 1165.4767; found: 1165.4770.

#### 2.1.13. Sodium [methyl (2,3,6-tri-O-benzyl-α-d-glucopyranosyl)-(1→4)-(2,3-di-O-acetyl-β-d-glucopyranosyl-uronate)-(1→4)-2,3,6-tri-O-benzyl-α-d-glucopyranoside (**28**)

Compound **27** (1.013 g, 0.887 mmol) was oxidized according to general method H. The crude product was purified by silica gel chromatography (97:3 to 9:1 CH_2_Cl_2_/MeOH) to give **28** (597 mg, 60%) as a white foam. [α]_D_^25^ +23.8 (*c* 0.13, CHCl_3_); *R*_f_ 0.27 (97:3 CH_2_Cl_2_/MeOH); ^1^H NMR (CDCl_3_, 400 MHz) *δ* 7.41–7.20 (m, 30H, arom.), 5.12 (t, *J* = 9.0 Hz, 1H, H-3′), 5.01 (d, *J* = 3.1 Hz, 1H, H-1″), 4.97–4.86 (m, 3H, Bn-*C*H_2_, H-2′), 4.75–4.42 (m, 12H, 5 × Bn-C*H*_2_, H-1, H-1′), 4.07 (t, *J* = 9.0 Hz, 1H, H-4′), 3.91–3.81 (m, 2H, H-3, H-4), 3.77–3.69 (m, 4H, H-3″, H-4″, H-5′, H-6″a), 3.65–3.58 (m, 4H, H-5, H-6a,b, H-6″b), 3.53 (t, *J* = 9.4 Hz, 1H, H-5″), 3.45 (dd, *J* = 3.6 Hz, *J* = 9.2 Hz, 1H, H-2), 3.39 (dd, *J* = 3.2 Hz, *J* = 9.8 Hz, 1H, H-2″), 3.36 (s, 1H, H-4″-OH), 3.33 (s, 3H, C-1-OC*H*_3_), 1.94, 1.84 (2 × s, 6H, 2 × Ac-C*H*_3_) ppm; ^13^C NMR (CDCl_3_, 100 MHz) *δ* 170.1, 169.4 (2C, 2 × C_q_ Ac), 139.0, 138.7, 138.3, 137.9, 137.6, 137.5 (6C, 6 × C_q_ arom.), 128.8–127.3 (30C, arom.), 100.0 (1C, C-1′), 98.4 (1C, C-1), 98.2 (1C, C-1″), 80.2 (1C, C-3″), 79.9 (1C, C-3), 79.5 (1C, C-2″), 79.1 (1C, C-2), 77.0 (1C, C-4), 76.5 (1C, C-4′), 75.6, 75.2 (2C, 2 × Bn-*C*H_2_), 74.8 (1C, C-5′), 73.8 (1C, C-3′), 73.8, 73.7, 73.6, 73.1 (4C, 4 × Bn-*C*H_2_), 72.2 (1C, C-2′), 71.3 (1C, C-4″), 70.7 (1C, C-5″), 69.8 (1C, C-5), 69.6 (1C, C-6), 67.6 (1C, C-6″), 55.4 (1C, C-1-O*C*H_3_), 20.9, 20.8 (2C, 2 × Ac-*C*H_3_) ppm; UHR ESI-QTOF (positive ion): *m*/*z* calcd for [C_65_H_72_O_19_ + Na]^+^: 1179.4560; found: 1179.4559.

#### 2.1.14. Methyl (2-azido-3,4-di-O-benzoyl-6-O-benzyl-2-deoxy-α-d-glucopyranosyl)-(1→4)-[2,3-di-O-benzoyl-6-O-(2-naphthyl)methyl-β-d-glucopyranosyl]-(1→4)-2,3,6-tri-O-benzyl-α-d-glucopyranoside (**30**)

Compound **17** [33] (240 mg, 0.516 mmol, 1.4 equiv.) was glycosylated with **29** (390 mg, 0.369 mmol) according to general method **E**. The crude product was purified by silica gel chromatography (6:4 *n*-hexane/acetone) to give **30** (345 mg, 64%) as a colourless syrup. [α]_D_^25^ +31.4 (*c* 0.17, CHCl_3_); *R*_f_ 0.46 (6:4 *n*-hexane/acetone); ^1^H NMR (CDCl_3_, 500 MHz) *δ* 7.98–6.93 (m, 47H, arom.), 5.82 (td, *J* = 6.3 Hz, *J* = 10.5 Hz, 1H, H-3″), 5.71 (td, *J* = 6.2 Hz, *J* = 9.5 Hz, 1H, H-3′), 5.50 (td, *J* = 5.4 Hz, *J* = 10.0 Hz, 1H, H-4″), 5.42–5.33 (m, 2H, H-1″, H-2′), 5.13 (dd, *J* = 5.1 Hz, *J* = 11.6 Hz, 1H, Bn-*C*H_2_a), 4.86–4.78 (m, 2H, H-1′, Bn-C*H*_2_b), 4.75–4.33 (m, 7H, H-1, 2 × Bn-*C*H_2_, NAP-*C*H_2_), 4.30–4.26 (m, 2H, H-4′, Bn-C*H*_2_a), 4.15–4.12 (m, 1H, H-5″), 4.08 (dd, *J* = 4.2 Hz, *J* = 12.0 Hz, 1H, Bn-*C*H_2_b), 4.01–3.95 (m, 1H, H-4), 3.92–3.88 (m, 1H, H-3), 3.84–3.76 (m, 2H, H-6′a,b), 3.69–3.64 (m, 1H, H-6a), 3.54–3.52 (m, 2H, H-5, H-5′), 3.49–3.47 (m, 1H, H-2), 3.46–3.42 (m, 1H, H-6b), 3.34 (dt, *J* = 4.0 Hz, *J* = 10.6 Hz, 1H, H-2″), 3.26 (s, 3H, C-3′-OC*H*_3_), 3.25–3.18 (m, 2H, H-6″a,b) ppm; ^13^C NMR (CDCl_3_, 125 MHz) *δ* 165.5, 165.3, 165.2, 165.1 (4C, 4 × C=O Bz), 139.6, 139.3, 137.7, 137.3, 135.9, 132.9, 129.5, 129.2, 129.0, 128.9 (11C, 11 × C_q_ arom.), 133.3–125.8 (47C, arom.), 100.2 (1C, C-1′), 98.8 (1C, C-1″), 98.4 (1C, C-1), 80.3 (1C, C-3), 79.1 (1C, C-2), 77.0 (1C, C-4), 75.4 (1C, C-3′), 75.3 (1C, Bn-*C*H_2_), 75.0 (1C, C-4′), 74.9 (1C, C-5′), 73.8, 73.7, 73.6, 73.5 (4C, NAP-*C*H_2_, 3 × Bn-*C*H_2_), 72.9 (1C, C-2′), 70.3 (1C, C-3″), 70.0 (1C, C-5″), 69.6 (1C, C-5), 69.3 (1C, C-6′), 69.0 (1C, C-4″), 67.8 (1C, C-6), 67.5 (1C, C-6″), 61.4 (1C, C-2″), 55.3 (1C, C-1-O*C*H_3_) ppm; UHR ESI-QTOF (positive ion): *m*/*z* calcd for [C_86_H_81_N_3_O_19_ + Na_2_]^2+^: 752.7624; found: 752.7623.

#### 2.1.15. Methyl (2-azido-3,4-di-O-benzoyl-6-O-benzyl-2-deoxy-α-d-glucopyranosyl)-(1→4)-(2,3-di-O-benzoyl-β-d-glucopyranosyl)-(1→4)-2,3,6-tri-O-benzyl-α-d-glucopyranoside (**31**)

Compound **30** (330 mg, 0.226 mmol) was converted to **31** according to general method G. The crude product was purified by silica gel chromatography (6:4 *n*-hexane/EtOAc) to give **31** (245 mg, 82%) as a colourless syrup. [α]_D_^25^ +5.4 (*c* 0.13, CHCl_3_); *R*_f_ 0.38 (6:4 *n*-hexane/EtOAc); ^1^H NMR (CDCl_3_, 500 MHz) *δ* 7.90–7.17 (m, 40H, arom.), 5.80 (t, *J* = 10.1 Hz, 1H, H-3″), 5.69 (t, *J* = 9.5 Hz, 1H, H-3′), 5.48 (t, *J* = 9.8 Hz, 1H, H-4″), 5.36 (d, *J* = 3.8 Hz, 1H, H-1″), 5.29 (t, *J* = 8.9 Hz, 1H, H-2′), 5.00 (d, *J* = 11.1 Hz, 1H, Bn-*C*H_2_a), 4.86 (d, *J* = 11.1 Hz, 1H, Bn-C*H*_2_b), 4.80 (d, *J* = 12.3 Hz, 1H, Bn-C*H*_2_a), 4.71 (d, *J* = 11.9 Hz, 1H, Bn-*C*H_2_a), 4.68–4.64 (m, 2H, H-1′, Bn-*C*H_2_b), 4.58–4.55 (m, 2H, H-1, Bn-C*H*_2_b), 4.48 (d, *J* = 12.1 Hz, 1H, Bn-C*H*_2_b), 4.34 (d, *J* = 12.0 Hz, 1H, Bn-C*H*_2_b), 4.18 (t, *J* = 9.4 Hz, 1H, H-4′), 4.11 (t, *J* = 7.1 Hz, 1H, H-5″), 3.93–3.83 (m, 2H, H-3, H-4), 3.75 (d, *J* = 12.0 Hz, 1H, H-6′a), 3.62 (d, *J* = 10.9 Hz, 1H, H-6a), 3.57–3.49 (m, 5H, H-2, H-5, H-6′a, H-6″a,b), 3.39 (d, *J* = 10.1 Hz, 1H, H-6b), 3.35–3.29 (m, 2H, H-2″, H-5′), 3.27 (s, 3H, C-3′-OC*H*_3_), 2.12–2.10 (m, 1H, H-6′-OH) ppm; ^13^C NMR (CDCl_3_, 125 MHz) *δ* 165.5, 165.2, 165.2, 165.1 (4C, 4 × C=O Bz), 139.4, 138.3, 137.6, 137.2, 129.4, 129.2, 128.9, 128.8 (8C, 11 × C_q_ arom.), 133.4–127.5 (40C, arom.), 100.1 (1C, C-1′), 99.1 (1C, C-1″), 98.4 (1C, C-1), 80.0 (1C, C-3), 79.0 (1C, C-2), 77.1 (1C, C-4), 75.5 (1C, Bn-*C*H_2_), 75.4 (1C, C-3′), 74.7 (1C, C-4′), 74.5 (1C, C-5′), 73.8, 73.7, 73.6 (3C, 3 × Bn-*C*H_2_), 72.8 (1C, C-2′), 70.5 (1C, C-3″), 70.2 (1C, C-5″), 69.6 (1C, C-5), 69.1 (1C, C-4″), 67.8 (1C, C-6″), 67.5 (1C, C-6), 61.5 (1C, C-6′), 61.2 (1C, C-2″), 55.4 (1C, C-1-O*C*H_3_) ppm.

#### 2.1.16. Sodium [methyl (2-azido-3,4-di-O-benzoyl-6-O-benzyl-2-deoxy-α-d-glucopyranosyl)-(1→4)-(2,3-di-O-benzoyl-β-d-glucopyranosyl-uronate)-(1→4)-2,3,6-tri-O-benzyl-α-d-glucopyranoside (**32**)

Compound **31** (230 mg, 0.174 mmol) was oxidized according to general method H. The crude product was purified by silica gel chromatography (6:4 *n*-hexane/acetone) to give **32** (172 mg, 73%) as a colourless syrup. [α]_D_^25^ +4.4 (*c* 0.18, CHCl_3_); *R*_f_ 0.38 (6:4 *n*-hexane/acetone); ^1^H NMR (CDCl_3_, 500 MHz) *δ* 7.90–7.16 (m, 40H, arom.), 5.85 (t, *J* = 10.1 Hz, 1H, H-3″), 5.59 (t, *J* = 9.2 Hz, 1H, H-3′), 5.41 (t, *J* = 9.7 Hz, 1H, H-4″), 5.29 (d, *J* = 3.5 Hz, 1H, H-1″), 5.27 (t, *J* = 8.6 Hz, 1H, H-2′), 4.99 (d, *J* = 11.2 Hz, 1H, Bn-*C*H_2_a), 4.80 (d, *J* = 11.2 Hz, 1H, Bn-C*H*_2_b), 4.75–4.71 (m, 2H, 2 × Bn-C*H*_2_a), 4.65 (d, *J* = 7.7 Hz, 1H, H-1′), 4.58–4.55 (m, 2H, 2 × Bn-*C*H_2_b), 4.53 (d, *J* = 3.5 Hz, 1H, H-1), 4.47 (d, *J* = 11.8 Hz, 1H, H-1, Bn-C*H*_2_b), 4.44–4.40 (m, 1H, H-5″), 4.32–4.27 (m, 2H, H-4′, Bn-C*H*_2_b), 3.94–3.85 (m, 3H, H-3, H-4, H-5′), 3.62–3.56 (m, 3H, H-6a, H-6″a,b), 3.50–3.47 (m, 2H, H-2, H-5), 3.39 (d, *J* = 10.2 Hz, 1H, H-6b), 3.33 (dd, *J* = 3.8 Hz, *J* = 10.1 Hz, 1H, H-2″), 3.25 (s, 3H, C-3′-OC*H*_3_) ppm; ^13^C NMR (CDCl_3_, 125 MHz) *δ* 165.4, 165.3, 165.0, 164.9 (4C, 4 × C=O Bz), 138.9, 138.2, 137.5, 136.8, 129.3, 129.0, 128.9, 128.8 (8C, 11 × C_q_ arom.), 133.5–127.5 (40C, arom.), 99.7 (2C, C-1′, C-1″), 98.4 (1C, C-1), 80.2 (1C, C-3), 79.1 (1C, C-2), 77.1 (1C, C-4′), 76.5 (1C, C-4), 75.9 (1C, Bn-*C*H_2_), 74.3 (1C, C-3′), 74.0, 73.8 (2C, 2 × Bn-*C*H_2_), 73.7 (1C, C-5′), 73.5 (1C, Bn-*C*H_2_), 72.3 (1C, C-2′), 70.6 (1C, C-3″), 69.9 (1C, C-5″), 69.5 (1C, C-5), 69.3 (1C, C-4″), 68.8 (1C, C-6″), 67.3 (1C, C-6), 61.3 (1C, C-2″), 55.4 (1C, C-1-O*C*H_3_) ppm; UHR ESI-QTOF (positive ion): *m*/*z* calcd for [C_75_H_70_N_3_NaO_20_ + Na_2_]^2+^: 700.7117; found: 700.7117.

#### 2.1.17. Sodium [methyl (2-amino-3,4-di-O-benzoyl-2-deoxy-α-d-glucopyranosyl)-(1→4)-(2,3-di-O-benzoyl-β-d-glucopyranosyl-uronate)-(1→4)-α-d-glucopyranoside (**33**)

Compound **32** (165 mg, 0.122 mmol) was debenzylated in the presence of CH_3_COOH (8.0 µL, 0.134 mmol, 1.1 equiv.) according to general method B. The crude product was purified by silica gel chromatography (9:1 CH_3_CN/H_2_O) to give **33** (111 mg, 89%) as a colourless syrup. [α]_D_^25^ +64.4 (*c* 0.09, MeOH); *R*_f_ 0.31 (9:1 CH_3_CN/H_2_O); ^1^H NMR (MeOD, 500 MHz) *δ* 7.95–7.23 (m, 20H, arom.), 5.93–5.89 (m, 1H, H-3′), 5.79–5.72 (m, 1H, H-3″), 5.51–5.49 (m, 2H, H-1″, H-2′), 5.45–5.40 (m, 1H, H-4″), 5.16 (d, *J* = 7.1 Hz, 1H, H-1′), 4.64–4.62 (m, 2H, H-1, H-4′), 4.33–4.29 (m, 2H, H-5′, H-5″), 3.80–3.53 (m, 8H, H-2, H-3, H-4, H-5, H-6a,b, H-6′a,b), 3.46–3.38 (m, 1H, H-2″), 3.28 (s, 3H, C-3′-OC*H*_3_) ppm; ^13^C NMR (MeOD, 125 MHz) *δ* 174.8 (1C, C=O, COOH), 167.6, 167.4, 166.9, 166.5 (4C, 4 × C=O Bz), 130.5, 130.3, 130.2 (4C, 4 × C_q_ arom.), 134.7–129.2 (20C, arom.), 100.8 (1C, C-1′), 100.6 (1C, C-1), 97.5 (1C, C-1″), 79.8 (1C, C-4), 76.8 (1C, C-5′), 76.7 (1C, C-3′), 75.5 (1C, C-4′), 73.5 (1C, C-2′), 73.0 (1C, C-3), 72.9 (1C, C-2), 72.7 (1C, C-3″), 71.8 (1C, C-5), 71.6 (1C, C-5″), 70.3 (1C, C-4″), 60.7 (2C, C-6, C-6″), 55.5 (1C, C-1-O*C*H_3_), 55.2 (1C, C-2″) ppm; UHR ESI-QTOF (positive ion): *m*/*z* calcd for [C_47_H_49_NO_20_ + Na]^+^: 970.2740; found: 970.2743.

### 2.2. Biological Investigations

#### 2.2.1. Investigation of the Anti-Inflammatory Effect on THP-1 Cells

The THP-1 human monocyte cell line was obtained from ATCC (TIB-202, Manassas, VA, USA) and cells were maintained in RPMI 1640 medium (Sigma-Aldrich, St. Louis, MI, USA) containing 10% heat-inactivated fetal bovine serum (FBS), 10 mM HEPES, 100 μg/mL penicillin/streptomycin, 50 mM 2-mercaptoethanol (all from Sigma-Aldrich) in a humidified incubator with 5% CO_2_ at 37 °C. TNFα secretion from THP-1 cells was assessed using ELISA, with supernatants collected 6 hrs after cells were treated with trisaccharides and/or lipopolysaccharide (LPS, Sigma-Aldrich). A TNFα ELISA kit was sourced from BD Biosciences, and measurements were carried out in accordance with the manufacturer’s instructions. An EnVision 2105 Multimode Plate Reader (Perkin Elmer, Waltham, MA, USA) was utilized for quantifying cytokine levels, and concentrations were calculated using the cubic logistic model with GraphPad Prism 9.1.2 for Windows (GraphPad Software).

#### 2.2.2. Investigation of the NF-κB Inflammatory Pathway

To examine the possible anti-inflammatory effects of these molecules, the NF-κB pathway was investigated on MCF-7 cells. In this experiment, 30,000 cells/well were seeded on round glass cover-slips placed into 24-well plates. The next day, cells were pre-incubated with the different materials in 30 µM HBSS for 24 h at 37 °C. After this, cells were washed twice with HBSS and incubated with TNF-α (50 ng/mL) for 30 min to stimulate the NF-κB inflammatory pathway. After the incubation time, cells were washed twice with HBSS and fixed with methanol/acetone 1:1 for 5 min at −20 °C. After this incubation time, cells were washed three times with HBSS, and the nonspecific binding sites were blocked with fetal bovine serum (FBS) for 15 min at room temperature. Cells were then incubated with 2 µg/mL anti-p65 antibody (RELA/NF-κB p65 Antibody (F-6) Alexa Fluor^®^ 488) (Santa Cruz Biotechnology, Inc., Heidelberg, Germany) for 1 h at 37 °C. After this incubation, cells were washed three times with HBSS, and cell nuclei were stained with DAPI (283 nM) for 5 min at 37 °C. After this, cells were washed once with HBSS, and round glass cover-slips were glued to the slides. Fluorescence microscopy measurements and analyses were carried out by a Zeiss Axioscope A1 (Zeiss, Jena, Germany) fluorescent microscope and ZEN 2011 Microsoft Software (Zeiss, Jena, Germany). The following filters were used to examine the samples: DAPI: excitation G 365 nm, emission BP 445/50 nm; fluorescein: excitation BP 470/40 nm, emission BP525/50 nm.

##### Investigation of the Inflammatory Markers Interleukin (IL)-1B and IL-10 on MCF-7 Cells

MCF-7 cells were seeded and cultured in 6-well plates for 24 h, until they formed a confluent monolayer. The next day, the medium was discarded and replaced with either 5 µM TNF-α, 30 µM trisaccharide (compound **12**) or 5 µM TNF-α +30 µM trisaccharide (compound **12**) containing complete growth medium. Cells were incubated for an additional 24 h. The treatment was performed in biological replicates (*n* = 3). Total RNA was isolated from treated and untreated (control) MCF-7 cells by TRI reagent^®®^ (Sigma-Aldrich, St. Louis, MI, USA) according to the manufacturer’s instructions. RNA from each sample (2000 ng) was reverse transcribed to cDNA using a Tetro cDNA Synthesis Kit in a final volume of 20 µL. The following primer pairs were used: *IL-1B* 5-CCACAGACCTTCCAGGAGAATG-3′ (sense), 5′-GTGCAGTTCAGTGATCGTACAGG-3′ (antisense), *IL-10* 5′-TCTCCGAGATGCCTTCAGCAGA-3′ (sense), and 5′-TCAGACAAGGCTTGGCAACCCA-3′ (antisense) (Sigma-Aldrich, St. Louis, MI, USA). HPRT1 was used as the internal reference gene. mRNA levels were measured using iQ™ SYBR^®^ Green Supermix (Bio-Rad Laboratories Inc., Hercules, CA, USA). Reactions were conducted according to the manufacturer’s protocol using the MyiQ2 two-colour real-time PCR detection system (Bio-Rad Laboratories Inc.). All real-time amplifications were measured in triplicates. Results were evaluated with Bio-Rad iQ5 software, Version 2.1 (Bio-Rad Laboratories Inc.) and changes in mRNA levels were calculated using the 2^40−Ct^ method. The PCR protocol included an initial enzyme activation (95 °C for 3 min), then 40 cycles of amplification (95 °C for 15 s, 65 °C for 1 min), followed by a final annealing step (55 °C for 20.5 min). At least 3 independent experiments were performed.

#### 2.2.3. Investigation of the Cell Growth Inhibitory Activity on MCF-7, A2780, WM35 and HaCaT Cell Lines

Effect of the synthesized compounds on cellular viability was investigated by the MTT assay using MCF-7 human breast cancer, A2780 human ovarian carcinoma, WM35 human melanoma and spontaneously immortalized HaCaT human keratinocyte cell lines. MCF-7 human breast cancer cells from ATCC were cultured in Dulbecco’s modified Eagle’s medium (DMEM) supplemented with l-glutamine, 10% FBS, and 1% penicillin/streptomycin. WM35 and A2780 cells were cultured in RPMI1640 Medium (ThermoFisher, Waltham, MA, USA) containing 10% fetal bovine serum (FBS, ThermoFisher) and penicillin and streptomycin antibiotics (ThermoFisher, Waltham, MA, USA). HaCaT cells were cultured in DMEM (ThermoFisher, Waltham, MA, USA) supplemented with FBS and penicillin/streptomycin. Cells were cultured in a humified thermostat at a constant temperature (37 °C) and in the presence of 5% CO_2_. Media were changed every 2–3 days and cells were regularly sub-cultured when confluence of the monolayer culture reached ca. 80%. To determine cellular viability, the MTT assay was used as described in our earlier works [30,35]. Briefly, the number of viable cells was indirectly assessed by measuring the conversion of 3-{4,5-dimethilthiasol-2-il}-2,5-diphenyltetrasolium bromide (MTT, Sigma-Aldrich, St. Louis, MI, USA) to coloured formazan crystals by mitochondrial dehydrogenases active in living cells. Cells were seeded in 96-well microplates (5000 cells per well density for MCF-7, A2780 and HaCaT and 10,000 cells per well density for WM35) and were cultured for 3 days and treated by the compounds daily. An equal amount of vehicle solvent (DMSO, Sigma-Aldrich) was used to treat the control group. Cells were then incubated with 1 mg/mL MTT for 3 h, and precipitated formazan crystals were dissolved in isopropanol supplemented with 10% 1 M HCl and 10% Triton X 100 (all from VWR). The concentration of formazan was assessed colorimetrically by measuring absorbance at 570 and 690 nm (MCF-7, FLUOstar OPTIMA, BMG Labtech.) or 565 nm (A2780, WM35 and HaCaT). Viability was calculated as percent of control based on the measured absorbance, and data were analyzed by IBM SPSS software version 20 (IBM, Armonk, NY, USA) or GraphPad Prism 8.3.1 (549) (GraphPad Software, La Jolla, CA, USA) using Student’s two-tailed, unpaired *t*-test (paired comparisons) or one-way ANOVA with Bonferroni’s post hoc test (multiple comparisons), and *p*-values < 0.05 were regarded as significant differences. Graphs were plotted using GraphPad Prism 8.3.1 (549).

#### 2.2.4. Investigation of the Antioxidant Activity of the Trisaccharides

##### TAC Assay

The Total Antioxidant Capacity (TAC) assay was carried out as described by Szőke et al. [36]. Briefly, the method is based on the scavenging of ABTS*•* radicals and their conversion to a colourless product. The decolourization induced by a compound is compared to the decolourization caused by Trolox, giving the Trolox equivalent antioxidant capacity (TEAC) value. Briefly, 10 µL sample, 20 µL of Myoglobin Working Solution and 150 µL of ABTS Substrate Working Solution were mixed and incubated for 5 min at room temperature. At the end of incubation, 100 mL of Stop Solution was added and endpoint absorbance at 405 nm was read. All experiments were performed in triplicates and repeated three times.

##### ORAC Assay

The ORAC assay was carried out as described by Glazer et al. [37] with modifications. Freshly prepared AAPH stock (37.5 mM) solution and fluorescein stock solution (75 mM) in phosphate buffer (pH 7.0) were used for the assay. The concentration of the test compounds was 100 µM. The final reaction volume of the wells was 200 µL (in a black-sided micro-plate), containing 65 µL of PBS buffer, 20 µL of fluorescein and 15 µL of compounds of interest, except the blank, and the reactions were initiated by adding 100 µL of AAPH stock solution. The mixture was incubated at 37 °C and the fluorescence (excitation wavelength of 485 nm and emission wavelength of 520 nm) was monitored every 2 min for 1 h by a FLUOstar OPTIMA (BMG LABTECH) plate reader. The area under the curve (AUC) was calculated as follows:

AUC = 0.5 + (R2/R1) + (R3/R1) + (R4/R1) + … + (Rn/R1) (1)

where R1 is the initial fluorescence reading at 0 min and Rn is the fluorescence reading at n min. Finally, the net AUC was obtained by subtracting the AUC of the blank from that of a sample.

AUCnet = AUC sample − AUC blank (2)

All measurements were carried out in triplicate and repeated three times for each tested compound. Data are expressed as the mean ± SEM.

##### FRAP Assay

The ferric-reducing ability of the investigated compounds was determined by the FRAP assay as described by Csepanyi et al. [38]. The assay stock solution contained acetate buffer (pH 3.6, 300 mM), TPTZ solution (10 mM) in 40 mM HCl and ferric chloride (FeCl_3_) solution (20 mM). The freshly prepared working solution contained 10 mL of acetate buffer and 1–1 mL of TPTZ and ferric chloride solution. The FRAP reagent was activated by incubation at 37 °C for 15 min.

The reaction mixture consisted of 190 µL FRAP working solution and 10 µL of the test compound (100 µM), except the blank sample in a 96-well plate. After 30 min of incubation at 37 °C, the absorbance of the coloured product was measured at 593 nm. The FRAP values were expressed as Trolox equivalents (µM), based on the Trolox standard calibration curve. All measurements were carried out in triplicate and repeated three times for each investigated compound. Data are expressed as the mean ± SEM.

##### CUPRAC Assay

The CUPRAC assay was carried out as described by Apak et al. [39]. This assay is based on the reduction of copper(II) ion to copper(I) at neutral pH. The reduced copper(I) forms a complex with neocuproine (Nc), changing the colour, which can be measured at 450 nm. A total of 50 μL copper(II) chloride solution (10 mM), 50 μL ammonium acetate buffer (pH 7.0), 50 μL of Nc solution (7.5 mM, in 96% EtOH) and 50 μL of purified water were mixed. Finally, 5 μL freshly prepared solution of the test compounds (in 96% EtOH at 100 µM) was added and incubated for an hour. At the end, the absorbance was read at 450 nm. Trolox was used as a reference, and the results were expressed as TEAC values. All experiments were performed in triplicates and repeated three times.

## 3. Results

### 3.1. Synthesis of the Trisaccharides

The monosaccharide building blocks used for the synthesis of non-glycosaminoglycan trisaccharides (**16** [34] and **17** [33]) were already synthesized in our previous work. The protecting group strategy was designed in such a way that the uronic acid function can be performed at the oligosaccharide level; for this, the primary position of the precursor glucose derivative was protected with an –ONAP ether group (NAP = (2-naphthyl)methyl), which can be selectively removed in the presence of other groups. Positions containing –OH or –OSO_3_Na in the final products were protected with benzyl groups. The trisaccharides containing d-glucuronic acid were constructed using disaccharide acceptors containing a uronic acid precursor and monosaccharide donors.

**Scheme 1:**
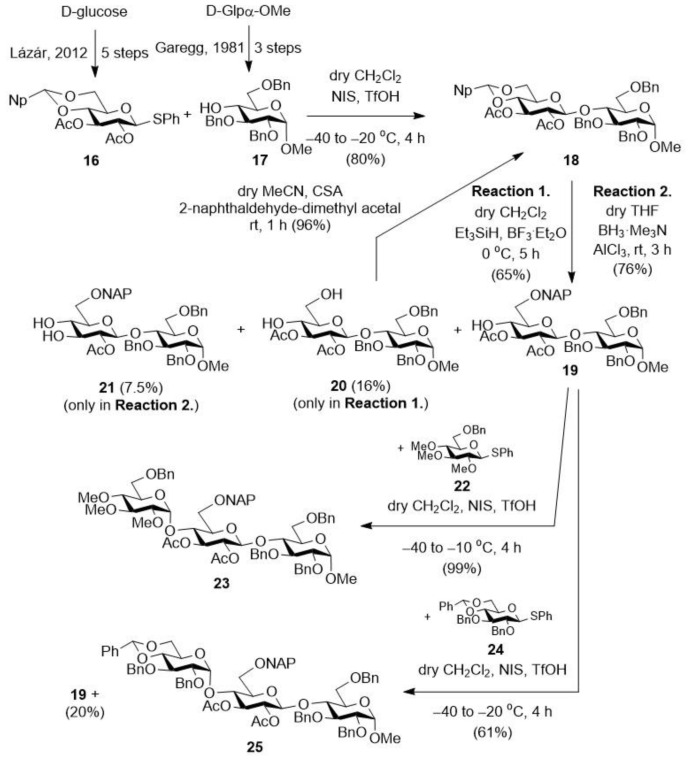
Preparation of the planned protected trisaccharides (**23** and **25**) starting form monosaccharide building blocks (**16** [34] and **17** [33])**.** NAP = (2-naphthyl)methyl; Np = (2-(naphthyl)methylene).

We started the synthesis with the preparation of the properly protected precursor disaccharide acceptor required for the production of non-glycosaminoglycan trisaccharides (Scheme 1).

For this, the monosaccharide acceptor (**17**) [33] was glycosylated with the protected thioglycoside donor (**16**) [34] in dry CH_2_Cl_2_ using the NIS/TfOH promoter system. In the reaction, the expected protected disaccharide (**18**) was formed with good yield and excellent stereoselectivity thanks to the acetyl group in position 2 of the donor (which, being a participating group, controls the stereochemistry of the resulting glycosidic bond). We then converted this derivative (**18**) into an acceptor in order to construct the trisaccharide skeleton. For this, the 4,6-*O*-Np-acetal ring of the non-reducing end glucose unit was selectively opened to form a NAP ether group at position C-6 and to liberate a hydroxyl group at position C-4. The ring-opening reaction was first carried out in dry CH_2_Cl_2_ using the Et_3_SiH/BF_3_·Et_2_O reagent combination [40]. In the reaction, the expected 4-OH-containing disaccharide was formed with good regioselectivity, but with only a moderate yield, and as a by-product, we identified the 4,6-di-OH derivative (**20**), which is a decomposition product formed during the hydrolysis of the acetal ring. Formation of the 4-ONAP/6-OH derivative was not observed. By re-acetalizing the diol (**20**) with 2-naphthaldehyde-dimethyl acetal reagent and using anhydrous 10-camphorsulfonic acid (CSA) catalyst, we obtained the starting, fully protected disaccharide (**18**), which could be used again in a ring-opening reaction, thus minimizing the loss resulting from hydrolysis.

The ring-opening reaction was repeated using the BH_3_·Me_3_N/AlCl_3_ reagent combination [41,42]. Fortunately, the yield was improved, the 6-ONAP derivative (**19**) was formed with a good yield (76%), and a partially deacetylated product (**21**) was identified as the only by-product, with a 7.5% yield.

Next, the thus-obtained disaccharide acceptor (**19**) was first glycosylated with the 2,3,4-tri-OMe-containing monosaccharide donor (**22**). The expected, protected trisaccharide (**23**), which was also synthesized in our previous work, was formed in the reaction with good yield and stereoselectivity [30]. The previously used disaccharide acceptor (**19**) was also glycosylated with the acetal-protected monosaccharide donor (**24**). In the reaction, the expected, protected trisaccharide (**25**) was formed with good yield and stereoselectivity, and we also recovered the unreacted acceptor disaccharide (**19**), which can be used again in the coupling reaction.

**Scheme 2:**
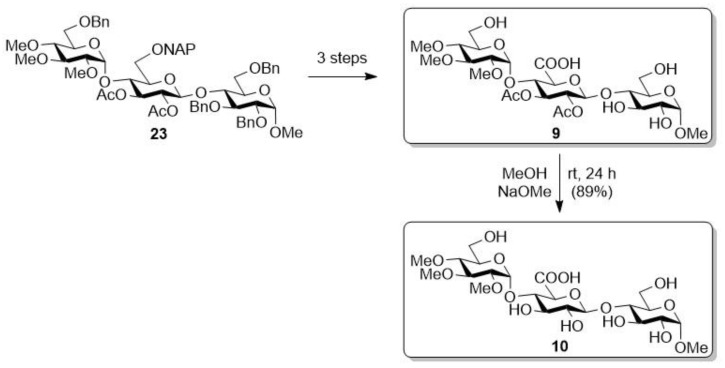
Transformation of the –OMe group containing protected trisaccharide (**23**) [30].

The trisaccharide, containing methyl groups on the non-reducing glucose unit (**23**), was further transformed in three steps into a derivative containing free –OHs and acetyl groups (Scheme 2, **9**), based on our previous work, and then, under Zemplén conditions, the acetyl groups on the uronic acid unit were removed, thus reaching a derivative with free –OHs and –OMe groups (**10**). In our previous studies, these derivatives (**9**, **10**) were prepared, but their biological activity was not tested, so we produced them again for the present biological studies [30].

**Scheme 3:**
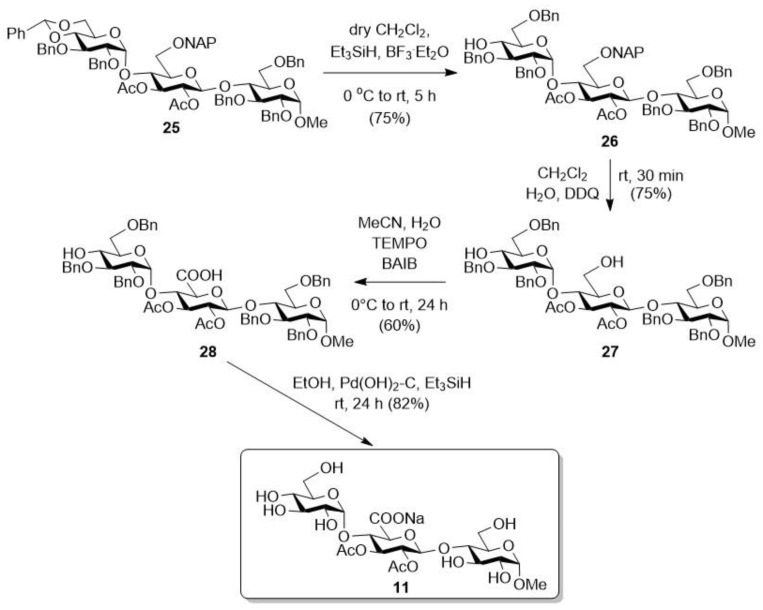
Transformation of the second protected trisaccharide (**25**).

After that, we started the transformation of another protected trisaccharide (Scheme 3, **25**). To do this, we first opened the benzylidene acetal protecting group by using the Et_3_SiH/BF_3_·Et_2_O reagent combination, thus obtaining the 6-OBn-containing derivative (**26**). Afterwards, in aqueous CH_2_Cl_2_, the NAP group on the middle sugar unit was removed under oxidative conditions, using 2,3-dichloro-5,6-dicyano-1,4-benzoquinone (DDQ) [43], thereby freeing the hydroxyl group at position C-6 of this unit (**27**). The resulting primary –OH was then oxidized to carboxylic acid using a TEMPO/BAIB reagent combination in aqueous acetonitrile, thus successfully converting the middle unit into glucuronic acid (**28**). By removing the benzyl groups by catalytic hydrogenation, a derivative containing free hydroxyls and acetyl groups on the uronic acid unit (**11**) was obtained, which was later used in biological studies.

**Scheme 4:**
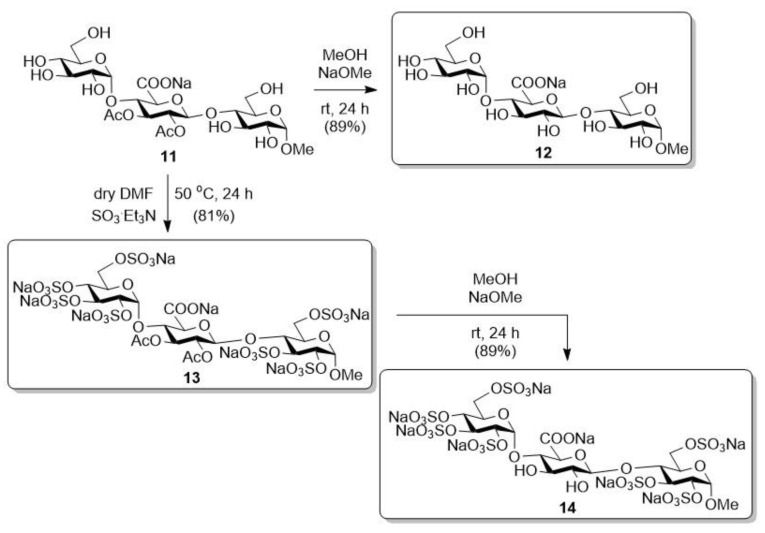
Transformation of the trisaccharide containing free hydroxyls and acetyl groups (**11**).

After that, this derivative (**11**) was further transformed into the planned target compounds (**12**–**14**) (Scheme 4). On the one hand, the acetyl groups were removed from the molecule under Zemplén conditions, thus obtaining the completely free derivative (**12**). On the other hand, a compound containing free hydroxyls and acetyl groups (**11**) was *O*-sulphated in dry DMF using the SO_3_·Et_3_N complex, thus producing the persulphated/acetylated derivative (**13**) in good yield. Finally, the acetyl groups on the uronic acid unit were selectively removed from this compound (**13**) under alkaline conditions, thus obtaining the sulphate/–OH-containing derivative (**14**) [44].

With this, we produced six non-glycosaminoglycan heparin analogue trisaccharides with diverse (methyl, hydroxyl, acetyl, sulphate) functional group patterns.

**Scheme 5:**
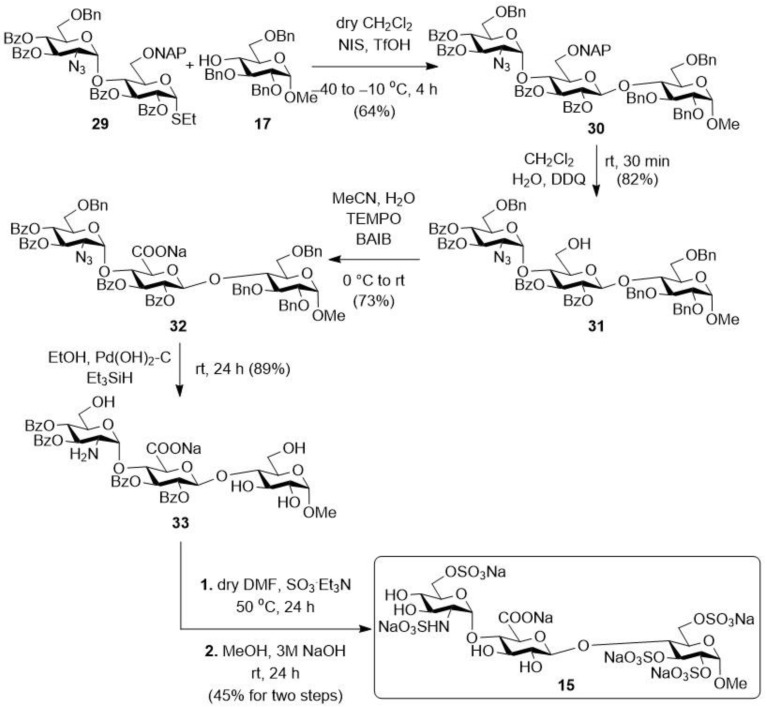
Synthesis of trisaccharide derivatives containing glucosamine moiety (**15**).

In the further part of the research, we also produced a glycosaminoglycan analogue in order to investigate how the presence of the –NH group affects its biological activity (Scheme 5). For this, the previously used monosaccharide acceptor (**17**) was glycosylated with the disaccharide donor containing a 2-azido-glucose unit at the non-reducing end (**29**). The expected protected trisaccharide (**30**) was formed in the reaction with good yield and stereoselectivity. After that, the NAP-protecting group was removed from position C-6 of the glucuronic acid precursor unit under oxidative conditions, thereby freeing the primary hydroxyl group (**31**). The carboxyl function was formed in aqueous acetonitrile, by oxidation of the primary –OH, using the TEMPO/BAIB reagent combination. In the reaction, the uronic acid-containing molecule (**32**) was formed with a good yield (73%), from which the benzyl ether groups were removed by catalytic hydrogenation, and the azido group was simultaneously reduced to –NH_2_ (**33**). The final functionalization pattern was formed on this derivative (**33**) obtained in two additional steps. First, the free –OHs and the –NH_2_ were sulphated using SO_3_·Et_3_N complex at 50 °C in dry DMF, and then the benzoyl ester-protecting groups were removed under alkaline conditions, thus releasing the four hydroxyls.

After the successful synthesis, the obtained seven trisaccharides were tested for their cell growth inhibitory, anti-inflammatory and antioxidant effects.

### 3.2. Biological Investigations

#### 3.2.1. Investigation of the Anti-Inflammatory Effect on THP-1 Cell Line

**Figure 3:**
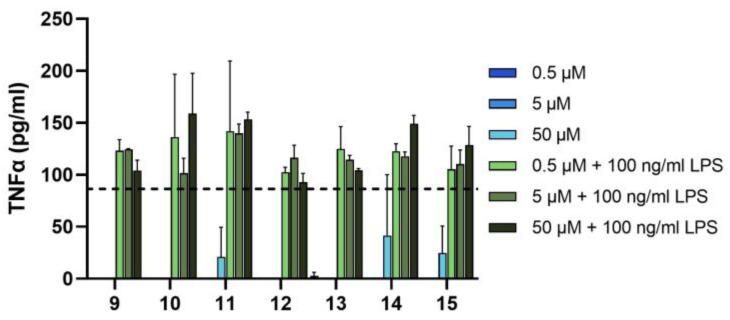
Trisaccharides do not show any anti-inflammatory effect on THP-1 cells. THP-1 cells were treated with vehicle or trisaccharides alone (black bars) or in combination with LPS (100 ng/mL, grey bars). TNFα production was determined with ELISA from supernatants. Bars represent mean ± SD of representative results from *n* ≥ 3 independent experiments. Vehicle-treated cells did not produce any TNFα. Dashed line shows TNFα release from cells treated with both the vehicle of trisaccharides and LPS. ND: not determined. LPS: lipopolysaccharide.

First, we investigated the anti-inflammatory activity of the seven derivatives (**9**–**15**) in the THP-1 cell line. To determine the putative anti-inflammatory effect of the synthesized trisaccharides, we applied them in a wide concentration range (0.5, 5, 50 μM) to the THP-1 monocytic cell line, both alone and in conjunction with the Toll-like receptor 2/4 agonist LPS (Figure 3). THP-1 cells respond to LPS stimulation with the secretion of proinflammatory cytokines, such as TNFα. Trisaccharides were applied to THP1 cells 10 min before treatment with LPS. Six hours after the initial treatment, we collected the supernatant of the cells, from which we measured the concentration of TNFα using ELISA. We found that without LPS, only the highest concentration of three trisaccharides (namely **11**, **14** and **15**) produced measurable amounts of the cytokine. In combination with LPS, none of the tested substances caused a statistically significant change in the amount of cytokine secreted. Based on these findings, we conclude that the newly synthesized trisaccharides do not exhibit pro- or anti-inflammatory effects in this model.

#### 3.2.2. Investigation of the Effect of the Trisaccharides on MCF-7 Cell Line

##### Investigation of the NF-κB Signalling Pathway

**Figure 4:**
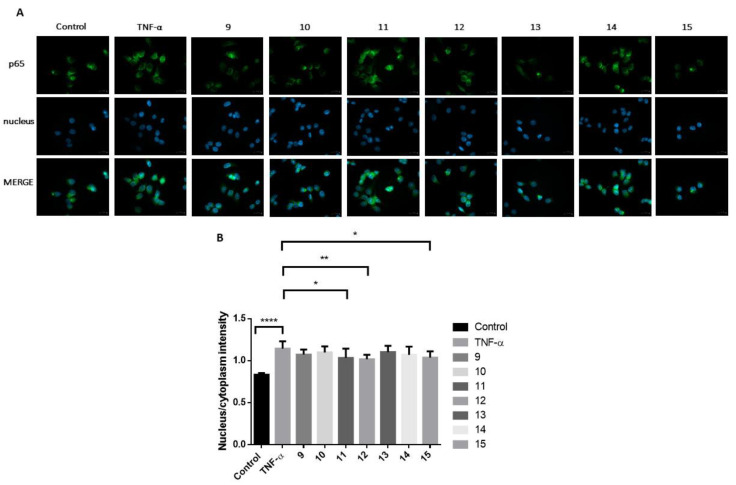
The effect of the synthesized trisaccharides (**9**–**15**) on the activation of the NF-κB inflammatory pathway by TNF-α in MCF-7 cells after 24 h pretreatment with the test substances at 30 μM concentration. (**A**) The NF-κB p65 subunit was labelled with a monoclonal antibody (green), localized in the cytoplasm in unstimulated control cells and translocated into the cell nuclei (blue) after TNF-α stimulation. (**B**) The selected molecules (**11**, **12** and **15**) significantly reduced (*p* < 0.05, *p* < 0.01, and *p* < 0.05 respectively) the p65 subunit’s nuclear translocation, showing an inhibitory potential on the NF-κB inflammatory pathway, compared to the TNF-α-treated, positive control cells. The effect of the test substances did not reach the control, unstimulated level. Mean ± S.D. values are shown; *n* = 10 cells were analyzed/group. * *p* < 0.05; ** *p* < 0.01; **** *p* < 0.0001.

Next, using another inflammation model, the inhibitory potential of the trisaccharide compounds (**9**–**15**) on NF-κB pathway activation was investigated in MCF-7 cells (Figure 4). Twenty-four-hour pretreatment of cells with the selected molecules (**11**, **12** and **15**) at a concentration of 30 μM significantly reduced (*p* < 0.05, *p* < 0.01, and *p* < 0.05 respectively) the NF-κB p65 subunit’s translocation into the cell nucleus from the cytoplasm caused by TNF-α, indicating the inhibition of this inflammatory pathway.

##### Investigation of the Inflammatory Markers Interleukin (IL)-1B and IL-10 on MCF-7 Cells

**Figure 5:**
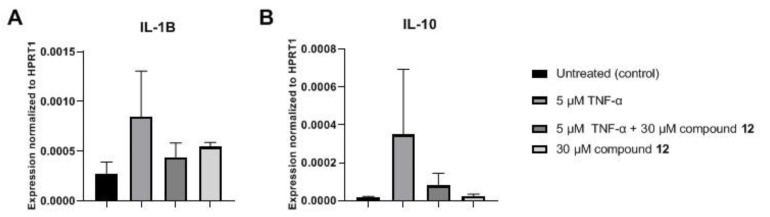
Expression level of IL-1B (**A**) and IL-10 (**B**). Gene expression level changes calculated by 2^40−ct^ method after 24 h treatment with the selected trisaccharide (**12**) and TNF-α. Data points are presented as mean (SEM) (*n* = 3).

Examining the possible anti-inflammatory effect of the selected trisaccharide derivative (**12**), the inflammatory markers interleukin (IL)-1B and IL-10 were investigated on MCF-7 cells using RT-qPCR. Treatment of breast cancer cells with TNF-α alone stimulated the release of IL-1B and IL-10. However, the levels of IL-1B and IL-10 decreased following treatment with the combination of this derivative (**12**) with TNF-α (Figure 5). As a result, the investigated trisaccharide (**12**) can exert an anti-inflammatory effect on the tested cell line.

##### Cytotoxicity Studies on MCF-7 Cells

**Figure 6:**
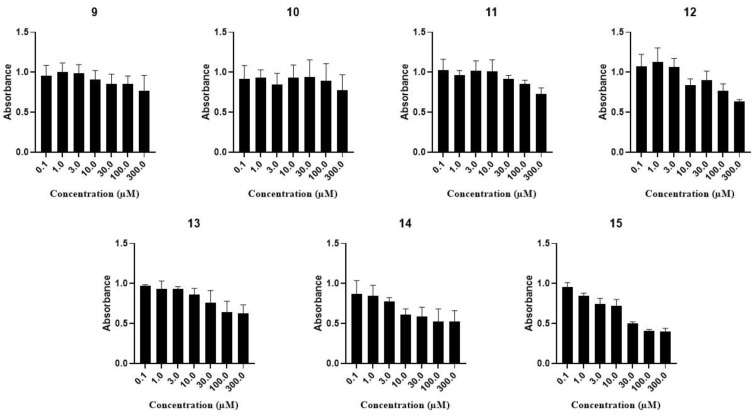
Cytotoxicity of the trisaccharides (**11**, **12**, **13**, **14**, **9**, **10** and **15**) on MCF-7 cells. Cells were incubated for 72 h with the test solutions. Viability was determined by the MTT assay after 24 h of treatment with the compounds applied in the indicated concentrations. Data are presented as mean SEM, *n* = 6 at each data point.

After that, we also examined the cytotoxic effect of our molecules. Our results have shown that some of our compounds (**11**, **12**, **13**, **14** and **15**) showed growth inhibitory effects on cancerous MCF-7 cell lines but only at high concentrations; therefore, no IC50 values were determined. Specifically, another two derivatives (**9** and **10**) did not exhibit a dose-dependent effect on viability (Figure 6).

##### Investigation of the Cell Growth Inhibitory Activity on A2780, WM35 and HaCaT Cell Lines

**Figure 7:**
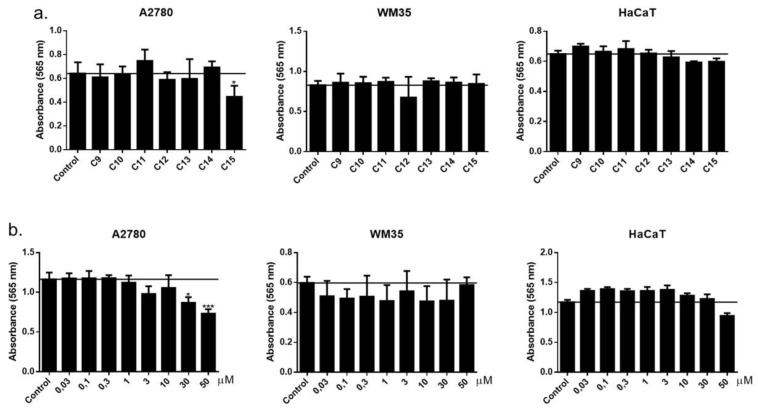
(**a**) Effect of different compounds (**9**–**15**) used in 50 µM on the viability of the investigated cell lines (non-cancerous and cancerous). (**b**) Concentration-dependent effect of the active compound (**15**) on the viability of A2780 ovarian carcinoma, WM35 melanoma and HaCaT keratinocyte cell lines. Viability was determined by the MTT assay after 72 h of treatment with the compounds applied in the indicated concentrations. Data are presented as mean ± SEM, *n* = 8 at each data point. * *p* < 0.05; *** *p* < 0.001.

The potential effects of trisaccharide derivatives (**9**–**15**) on the cellular viability of A2780 human ovarian carcinoma, WM35 human melanoma and HaCaT spontaneously immortalized human keratinocyte cell lines were investigated by the MTT assay. Of the trisaccharides tested, only one compound (**15**) reduced the viability of the A2780 ovarian carcinoma cells in 50 µM concentration, while the viability of the other two cell lines was not affected by trisaccharides. During our further investigations, we assessed this compound (**15**) in a wider concentration range on the above-mentioned three cell lines and found that the trisaccharide (**15**) inhibited the growth of A2780 cells only in higher concentrations (30 and 50 µM) and had no effect on cell viability on the other two cell types. In summary, we can conclude that these substances did not markedly influence the viability of the examined cell lines (Figure 7).

#### 3.2.3. Investigation of the Antioxidant Activity of the Trisaccharides

**Figure 8:**
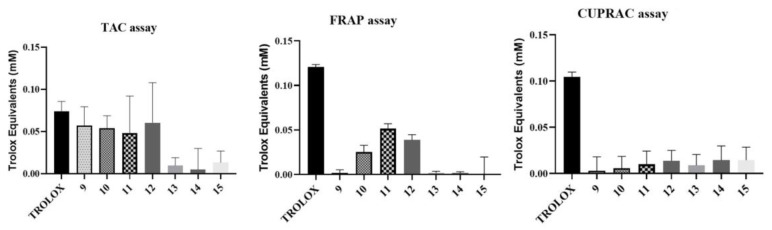
Antioxidant activity of the trisaccharides (**9**–**15**). Data are presented as mean ± SEM, *n* = 9 at each data point.

Finally, we also examined the antioxidant effect of trisaccharides. As depicted in Figure 8, limited reducing power (SET) of the molecules was detected by the CUPRAC assay at a neutral pH. Furthermore, at an acidic pH, employed in FRAP, three compounds (**10**, **11** and **12**) exhibited superior antioxidant effects compared to the other test molecules.

The results of the TAC assay (SET and HAT) also indicated some compounds (**9**, **10**, **11**, **12**) to possess some antioxidant action. However, none of the compounds were active in the ORAC assay, indicating that the molecules do not exert direct peroxyl and hydroxyl radical-scavenging effects. In summary, based on our measurement results, from the point of view of the antioxidant effect, it is favourable if the tested compound does not contain sulphate ester groups, although their effect depends on the mechanism of the test method. Based on the TAC assay, derivatives bearing -OMe groups and free -OH derivatives (**9**, **10**, **11** and **12**) were also found to be active. Based on this method, the presence of -OAc groups did not significantly affect the antioxidant effect. However, based on the FRAP assay, compounds without -OMe groups (**11** and **12**) showed the best activity.

## 4. Conclusions

Based on our previous synthetic work, we successfully synthesized seven heparin/heparosan analogue trisaccharide derivatives, of which six were non-glycosaminoglycan (Glc-GlcA-Glc) and one heparin-related (GlcN-GlcA-Glc) sequences. The synthesis of the non-glycosaminoglycan derivatives took place in 18–20 steps, and the synthesis of the glucosamine derivative took place in 26 steps.

After the synthesis, we investigated the possible biological activity of our compounds. The cell viability tests, which were performed on A2780, HaCaT, MCF-7 and WM-35 cell lines, revealed that the trisaccharide derivatives we produced did not have a significant cell growth inhibitory effect.

We also tested the anti-inflammatory effect of our compounds on THP1 and MCF-7 cell lines. It is clear from the obtained results that the trisaccharides had no anti-inflammatory effect on THP1 cells, but some compounds had a detectable effect on the MCF-7 cell line. The totally free trisaccharide (**12**) proved to be the most effective derivative, whose anti-inflammatory effect was supported by confirming the activation of the NF-κB pathway and by RT-qPCR (also with an effect on IL-1B and IL-10 interleukin markers) tests on the MCF-7 cell line. Based on the tests carried out, this compound has a selective anti-inflammatory effect, depending on the cell type.

Finally, we also examined the antioxidant effect of our compounds. It can be seen that, depending on the mechanism of the antioxidant effect, some of our compounds have significant antioxidant activity (TAC assay: **9**, **10**, **11**, **12**; FRAP assay: **10**, **11**, **12**). These results correlate well with the results of the anti-inflammatory effect measurements.

In summary, we successfully proved that heparin/heparosan analogue derivatives with a smaller number of members can also have biological activity, since one of the trisaccharide derivatives we prepared has promising anti-inflammatory and antioxidant effects. From the results of our previous [30] and current work, it is clear that the compounds that carried both –OMe and sulphate ester groups (heparin analogue derivatives) had an inhibitory effect on cell growth. Anti-inflammatory and antioxidant effects were shown by the compounds with free hydroxyl groups (they did not contain either the –OMe or sulphate ester part, and were heparosan analogues). The position of the sulphate ester groups is also important, but we cannot draw far-reaching conclusions from this point of view, since our compounds always contain this functional group in the same position. What all derivatives had in common was that they contained a glucuronic acid moiety.

## Data Availability

The original contributions presented in the study are included in the article/supplementary material, further inquiries can be directed to the corresponding authors.

## Supplementary Materials

The following supporting information can be downloaded at https://www.mdpi.com/article/10.3390/biom14091052/s1. Figure S1: ^1^H and ^13^C NMR spectra of compound **9**. Figure S2: ^1^H, ^13^C, COSY and HSQC NMR spectra of compound **10**. Figure S3: ^1^H, ^13^C, COSY and HSQC NMR spectra of compound **11**. Figure S4: ^1^H, ^13^C, COSY, HMBC and HSQC NMR spectra of compound **12**. Figure S5: ^1^H, ^13^C, COSY and HSQC NMR spectra of compound **13**. Figure S6: ^1^H, ^13^C, COSY, HMBC and HSQC NMR spectra of compound **14**. Figure S7: ^1^H, ^13^C, COSY and HSQC NMR spectra of compound **15**. Figure S8: ^1^H, ^13^C, COSY and HSQC NMR spectra of compound **18**. Figure S9: ^1^H, ^13^C, COSY and HSQC NMR spectra of compound **19**. Figure S10: ^1^H, ^13^C, COSY and HSQC NMR spectra of compound **20**. Figure S11: ^1^H, ^13^C, COSY and HSQC NMR spectra of compound **21**. Figure S12: ^1^H, ^13^C, COSY and HSQC NMR spectra of compound **25**. Figure S13: ^1^H, ^13^C, COSY and HSQC NMR spectra of compound **26**. Figure S14: ^1^H, ^13^C NMR spectra of compound **27**. Figure S15: ^1^H, ^13^C, COSY and HSQC NMR spectra of compound **28**. Figure S16: ^1^H, ^13^C, COSY and HSQC NMR spectra of compound **30**. Figure S17: ^1^H, ^13^C, COSY and HSQC NMR spectra of compound **31**. Figure S18: ^1^H, ^13^C, COSY and HSQC NMR spectra of compound **32**. Figure S19: ^1^H, ^13^C, COSY and HSQC NMR spectra of compound **33**.

## References

[1] Murata K., Ochiai Y., Akashio K. (1985). Polydispersity of acidic glycosaminoglycan components in human liver and the changes at different stages in liver cirrhosis. Gastroenterology.

[2] Jackson R.L., Busch S.J., Cardin A.D. (1991). Glycosaminoglycans: Molecular properties, protein interactions, and role in physiological processes. Physiol. Rev..

[3] Yeung B.K.S., Chong P.Y.C., Petillo P.A. (2002). Synthesis of glycosaminoglycans. J. Carbohydr. Chem..

[4] Grand E., Kovensky J., Pourceau G., Toumieux S., Wadouachi A., Rauter A.P., Lindhorst T., Queneau Y. (2014). Chapter 11: Anionic oligosaccharides: Synthesis and applications. Carbohydrate Chemistry: Chemical and Biological Approaches.

[5] Mende M., Bednarek C., Wawryszyn M., Sauter P., Biskup M.B., Schepers U., Bräse S. (2016). Chemical Synthesis of Glycosaminoglycans. Chem. Rev..

[6] Jin L., Abrahams J.P., Skinner R., Petitou M., Pike R.N., Carrell R.W. (1997). The anticoagulant activation of antithrombin by heparin. Proc. Natl. Acad. Sci. USA.

[7] Desai U.R., Petitou M., Bjork I., Olson S.T. (1998). Mechanism of Heparin Activation of Antithrombin: Role of Individual Residues of the Pentasaccharide Activating Sequence in the Recognition of Native and Activated States of Antithrombin. J. Biol. Chem..

[8] Mulloy B., Hogwood J., Gray E., Lever R., Page C.P. (2016). Pharmacology of Heparin and Related Drugs. Pharmacol. Rev..

[9] Capila I., Linhardt R.J. (2002). Heparin–Protein Interactions. Angew. Chem. Int. Ed..

[10] Kjelle L., Lindahl U. (2018). Specificity of glycosaminoglycan-protein interactions. Curr. Opin. Struct. Biol..

[11] Weiss R.J., Esko J.D., Tor Y. (2017). Targeting heparin and heparan sulfate protein interactions. Org. Biomol. Chem..

[12] Cassinelli G., Naggi A. (2016). Old and new applications of non-anticoagulant heparin. Int. J. Cardiol..

[13] Glantz M.J., Burger P.C., Friedman A.H., Radtke R.A., Massey E.W., Schold S.C. (1994). Treatment of radiation-induced nervous system injury with heparin and warfarin. Neurology.

[14] Casu B., Guerrini M., Guglieri S., Naggi A., Perez M., Torri G., Cassinelli G., Ribatti D., Carminati P., Giannini G. (2004). Undersulfated and Glycol-Split Heparins Endowed with Antiangiogenic Activity. J. Med. Chem..

[15] Pisano C., Aulicino C., Vesci L., Casu B., Naggi A., Torri G., Ribatti D., Belleri M., Rusnati M., Presta M. (2005). Undersulfated, low-molecular-weight glycol-split heparin as an antiangiogenic VEGF antagonist. Glycobiology.

[16] Chiodelli P., Bugatti A., Urbinati C., Rusnati M. (2015). Heparin/Heparan Sulfate Proteoglycans Glycomic Interactome in Angiogenesis: Biological Implications and Therapeutical Use. Molecules.

[17] Ludwig R.J. (2009). Therapeutic use of heparin beyond anticoagulation. Curr. Drug Discov. Technol..

[18] Zhou H., Roy S., Cochran E., Zouaoui R., Chu C.L., Duffner J., Zhao G., Smith S., Galcheva-Gargova Z., Karlgren J. (2011). M402, a Novel Heparan Sulfate Mimetic, Targets Multiple Pathways Implicated in Tumor Progression and Metastasis. PLoS ONE.

[19] Vogt A.M., Pettersson F., Moll K., Jonsson C., Normark J., Ribacke U., Egwang T.G., Ekre H.-P., Spillmann D., Chen Q. (2006). Release of Sequestered Malaria Parasites upon Injection of a Glycosaminoglycan. PloS Pathog..

[20] Kwon P.S., Oh H., Kwon S.-J., Jin W., Zhang F., Fraser K., Hong J.J., Linhardt R.J., Dordick J.S. (2020). Sulfated polysaccharides effectively inhibit SARS-CoV-2 in vitro. Cell Discov..

[21] Kim S.Y., Jin W., Sood A., Montgomery D.W., Grant O.C., Fuster M.M., Fu L., Dordick J.S., Woods R.J., Zhang F. (2020). Characterization of heparin and severe acute respiratory syndrome-related coronavirus 2 (SARS-CoV-2) spike glycoprotein binding interactions. Antivir. Res..

[22] Yu Y., Shen M., Song Q., Xie J. (2018). Biological activities and pharmaceutical applications of polysaccharide from natural resources: A review. Carbohydr. Polym..

[23] Xie J.-H., Wang Z.-J., Shen M.-Y., Nie S.-P., Gong B., Li H.-S., Zhao Q., Li W.-J., Xie M.-Y. (2016). Sulfated modification, characterization and antioxidant activities of polysaccharide from *Cyclocarya paliurus*. Food Hydrocoll..

[24] Shao P., Chen X., Sun P. (2013). In vitro antioxidant and antitumor activities of different sulfated polysaccharides isolated from three algae. Int. J. Biol. Macromol..

[25] van Boeckel C.A.A., Beetz T., Vos J.N., Dejong A.J.M., van Aelst S.F., van den Bosch R.H., Mertens J.M.R., van der Vlugt F.A. (1985). Synthesis of a Pentasaccharide Corresponding to the Antithrombin III Binding Fragment of Heparin. J. Carbohydr. Chem..

[26] van Boeckel C.A.A., Petitou M. (1993). The Unique Antithrombin III Binding Domain of Heparin: A Lead to New Synthetic Antithrombotics. Angew. Chem. Int. Ed..

[27] Choay J., Lormeau J.-C., Petitou M., Sinay P., Fareed J. (1981). Structural studies on a biologically active hexasaccharide obtained from heparin. Ann. N.Y. Acad. Sci..

[28] Petitou M., Duchaussoy P., Lederman I., Choay J., Sinay P., Jacquinet J.C., Torri G. (1986). Synthesis of heparin fragments. A chemical synthesis of the pentasaccharide *O*-(2-deoxy-2-sulfamido-6-*O*-sulfo-α-d-glucopyranosyl) -(1→4)-*O*-(β-d-glucopyranosyluronic acid)-(1→4)-*O*-(2-deoxy-2-sulfamido-3,6-di-*O*-sulfo-α-d-glucopyranosyl)-(1→4)-*O*-(2-*O*-sulfo-α-l-idopyranosyluronic acid)-(1→4)-2-deoxy-2-sulfamido-6-*O*-sulfo-d-glucopyranose decasodium salt, a heparin fragment having high affinity for antithrombin III. Carbohydr. Res..

[29] Thunberg L., Backstrom G., Lindahl U. (1982). Further characterization of the antithrombin-binding sequence in heparin. Carbohydr. Res..

[30] Lisztes E., Mező E., Demeter F., Horváth L., Bősze S., Tóth B.I., Borbás A., Herczeg M. (2021). Synthesis and Cell Growth Inhibitory Activity of Six Non-glycosaminoglycan-Type Heparin-Analogue Trisaccharides. ChemMedChem.

[31] Peterson S., Liu J. (2012). Deciphering mode of action of heparanase using structurally defined oligosaccharides. J. Biol. Chem..

[32] Ni M., Elli S., Naggi A., Guerrini M., Torri G., Petitou M. (2016). Investigating Glycol-Split-Heparin-Derived Inhibitors of Heparanase: A Study of Synthetic Trisaccharides. Molecules.

[33] Garegg P.J., Hultberg H. (1981). A novel, reductive ring-opening of carbohydrate benzylidene acetals, with unusual regioselectivity. Carbohydr. Res..

[34] Lázár L., Mező E., Herczeg M., Lipták A., Antus S., Borbás A. (2012). Synthesis of the non-reducing end trisaccharide of the antithrombin-binding domain of heparin and its bioisosteric sulfonic acid analogues. Tetrahedron.

[35] Tóth B.I., Dobrosi N., Dajnoki A., Czifra G., Oláh A., Szöllősi A.G., Juhász I., Sugawara K., Paus R., Bíró T. (2011). Endocannabinoids modulate human epidermal keratinocyte proliferation and survival via the sequential engagement of cannabinoid receptor-1 and transient receptor potential vanilloid-1. J. Invest. Dermatol..

[36] Szőke K., Kajtár R., Gyöngyösi A., Czompa A., Fésüs A., Lőrincz E.B., Petróczi F.D., Herczegh P., Bak I., Borbás A. (2023). Pharmacological Evaluation of Newly Synthesized Cannabidiol Derivates on H9c2 Cells. Antioxidants.

[37] DeLange R.J., Glazer A.N. (1989). Phycoerythrin fluorescence-based assay for peroxy radicals: A screen for biologically relevant protective agents. Analyt Biochem..

[38] Csepanyi E., Szabados-Furjesi P., Kiss-Szikszai A., Frensemeier L.M., Karst U., Lekli I., Haines D.D., Tosaki A., Bak I. (2017). Antioxidant Properties and Oxidative Transformation of Different Chromone Derivatives. Molecules.

[39] Apak R., Güclü K., Özyürek M., Karademir S.E. (2004). Novel Total Antioxidant Capacity Index for Dietary Polyphenols and Vitamins C and E, Using Their Cupric Ion Reducing Capability in the Presence of Neocuproine: CUPRAC Method. J. Agric. Food Chem..

[40] Debenham S.D., Toone E.J. (2000). Regioselective reduction of 4,6-O-benzylidenes using triethylsilane and BF3·Et2O Tetrahedron. Asymmetry.

[41] Ek M., Garegg P.J., Hultberg H., Oscarson S. (1983). Reductive Ring Openings of Carbohydrate Benzylidene Acetals Using Borane-Trimethylamine and Aluminium Chloride. Regioselectivity and Solvent Dependance. J. Carbohydr. Chem..

[42] Ohlin M., Johnsson R., Ellervik U. (2011). Regioselective reductive openings of 4,6-benzylidene acetals: Synthetic and mechanistic aspects. Carbohydr. Res..

[43] Xia J., Abbas S.A., Locke R.D., Piskorz C.F., Alderfer J.L., Matta K.L. (2000). Use of 1,2-dichloro 4,5-dicyanoquinone (DDQ) for cleavage of the 2-naphthylmethyl (NAP) group. Tetrahedron Lett..

[44] Despras G., Bernard C., Perrot A., Cattiaux L., Prochiantz A., Lortat-Jacob H., Mallet J.-M. (2013). Toward Libraries of Biotinylated Chondroitin Sulfate Analogues: From Synthesis to In Vivo Studies. Chem. Eur. J..

