# Supplementary material for: Synthesis and Biological Profiling of Seven Heparin and Heparan Sulphate Analogue Trisaccharides
Source: Biomolecules. 2024 Aug 25;14(9):1052. doi: 10.3390/biom14091052 (PMC11429564; doi:10.3390/biom14091052)
Supplement: Supplementary file 1 [file biomolecules-14-01052-s001.zip › biomolecules-3104883-supplementary.pdf]

Synthesis and Biological Profiling of Seven Heparin and Heparan Sulphate Analogue Trisaccharides

Fruzsina Demeter [1], Zsófia Peleskei [1], Katalin Kútvölgyi [1], Ágnes Rusznyák [2,3], Ferenc Fenyvesi [2], Richárd Kajtár [4], Éva Sipos [4], István Lekli [4], Petra Molnár [5], Attila Gábor Szöllősi [5], Erika Lisztes [6], Balázs István Tóth [6,7], Anikó Borbás [1,8] and Mihály Herczeg [1,8,*]

1 Department of Pharmaceutical Chemistry, Faculty of Pharmacy, University of Debrecen, Egyetem tér 1, H-4032 Debrecen, Hungary; demeter.fruzsina@science.unideb.hu (F.D.); peleskei.zsofia@pharm.unideb.hu (Z.P.); kutvolgyi.katalin@pharm.unideb.hu (K.K.); borbas.aniko@pharm.unideb.hu (A.B.)
2 Department of Molecular and Nanopharmaceutics, Faculty of Pharmacy, University of Debrecen, Nagyerdei Körút 98, H-4032 Debrecen, Hungary; rusznyak.agnes@pharm.unideb.hu (Á.R.); fenyvesi.ferenc@pharm.unideb.hu (F.F.)
3 Institute of Healthcare Industry, University of Debrecen, Egyetem tér 1, H-4032 Debrecen, Hungary
4 Department of Pharmacodynamics, Faculty of Pharmacy, University of Debrecen, Nagyerdei Körút 98, H-4032 Debrecen, Hungary; kajtar.richard@euipar.unideb.hu (R.K.); sipos.eva@pharm.unideb.hu (É.S.); lekli.istvan@pharm.unideb.hu (I.L.)
5 Department of Immunology, University of Debrecen, Egyetem tér 1, H-4032 Debrecen, Hungary; molnar.petra@med.unideb.hu (P.M.); szollosi.attila@med.unideb.hu (A.G.S.)
6 Department of Physiology, University of Debrecen, P.O. Box 22, H-4012 Debrecen, Hungary; lisztes.erika@med.unideb.hu (E.L.); toth.istvan@med.unideb.hu (B.I.T.)
7 Department of Physiology, Medical School, University of Pécs, Szigeti út 12, H-7624 Pécs, Hungary
8 HUN-REN-DE Molecular Recognition and Interaction Research Group, University of Debrecen, Egyetem tér 1, H-4032 Debrecen, Hungary

* Correspondence: herczeg.mihaly@pharm.unideb.hu (M.H.)

Abstract: Researchers are paying increasing attention to the strongly negatively charged heteropolysaccharides in cells, in the extracellular matrix or in the cell wall. Examples of such molecules are glycosaminoglycans (e.g. heparin, heparan sulfate). It is well known from the literature that heparin and its derivatives have anti-inflammatory, angiogenic, metastatic and growth factor inhibitory activity. Herein, we present the efficient synthesis of six non-glycosaminoglycan (Glc-GlcA-Glc-sequenced) and one heparin-related (GlcN-GlcA-Glc-sequenced) trisaccharides with various functional group patterns. The anti-inflammatory, antioxidant and cell growth inhibitory/cytotoxic effects of the synthesized compounds were tested. Among the investigated molecules, we have found some derivatives with a promising anti-inflammatory and antioxidant effect.

Keywords: heparin; heparan-sulfate; trisaccharides; anti-inflammatory; antitumor activity

NMR spectra of the synthesized compounds

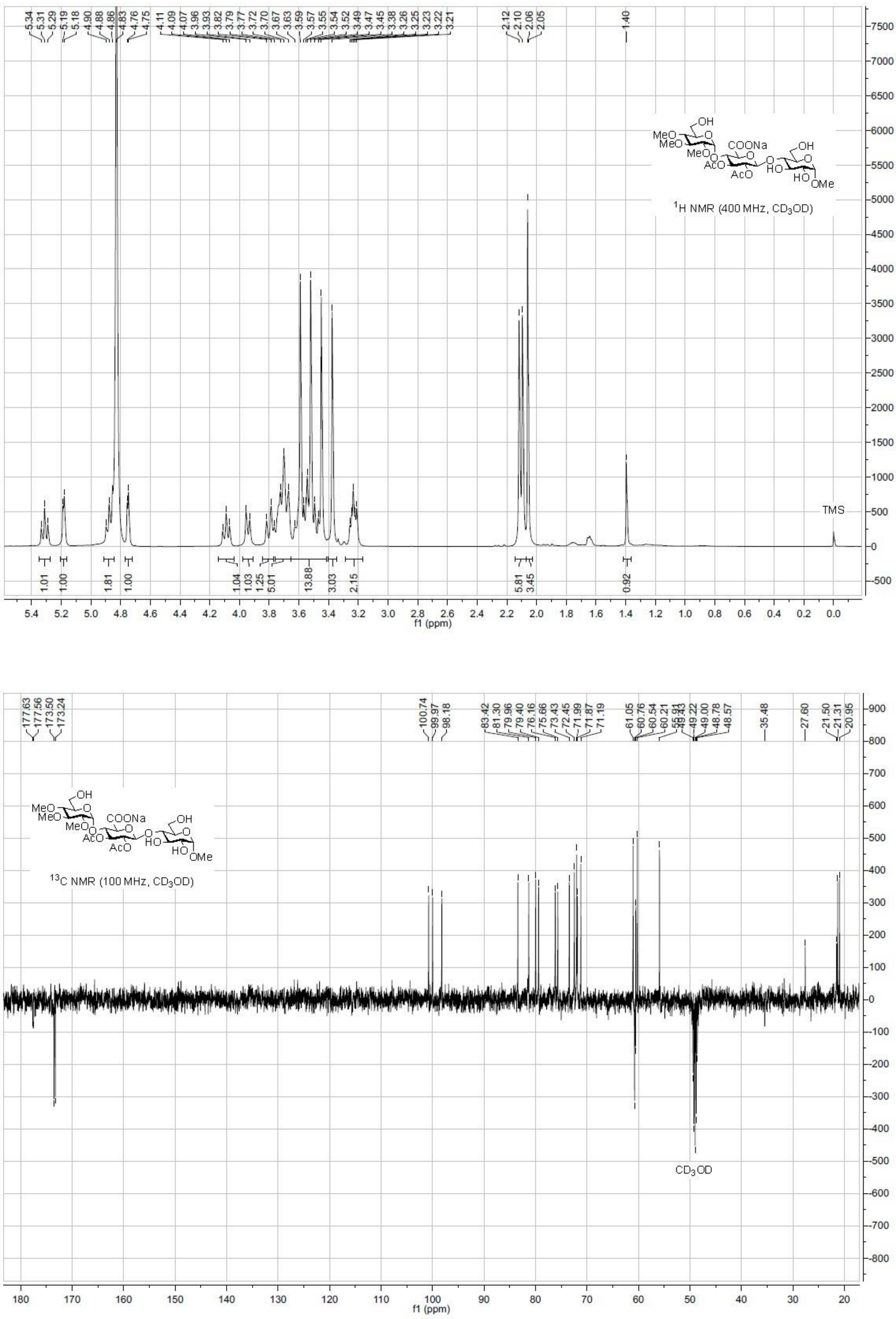

Figure S1. ^{1}H and ^{13}C NMR spectra of compound **9**.

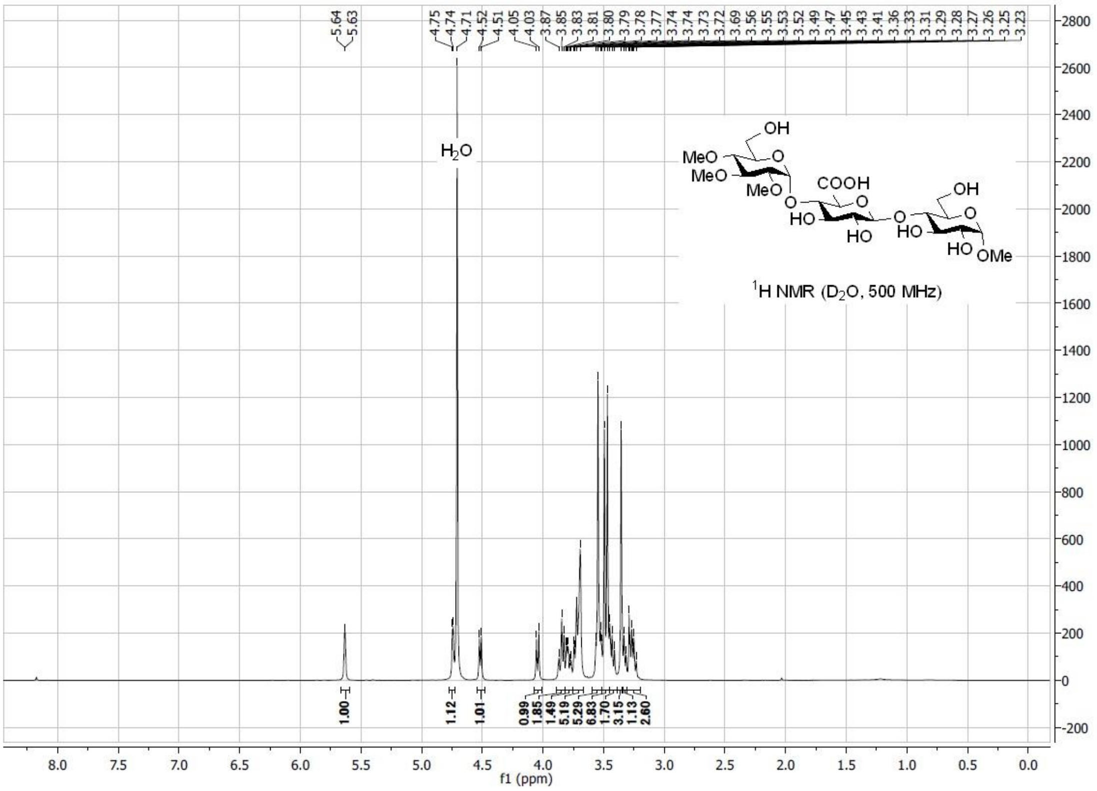
5.64
5.63
4.75
4.74
4.71
4.52
4.51
4.05
4.03
3.87
3.85
3.83
3.81
3.80
3.79
3.78
3.77
3.74
3.74
3.73
3.72
3.69
3.56
3.55
3.53
3.52
3.49
3.47
3.45
3.43
3.41
3.36
3.33
3.31
3.29
3.28
3.27
3.26
3.25
3.23
H2O
OH
MeO
MeO
MeO
O
COOH
OH
HO
HO
HO
HO
OMe
1H NMR (D2O, 500 MHz)
1.00
1.12
1.01
0.99
1.85
1.49
5.19
5.29
6.83
1.70
3.15
1.13
2.60
f1 (ppm)

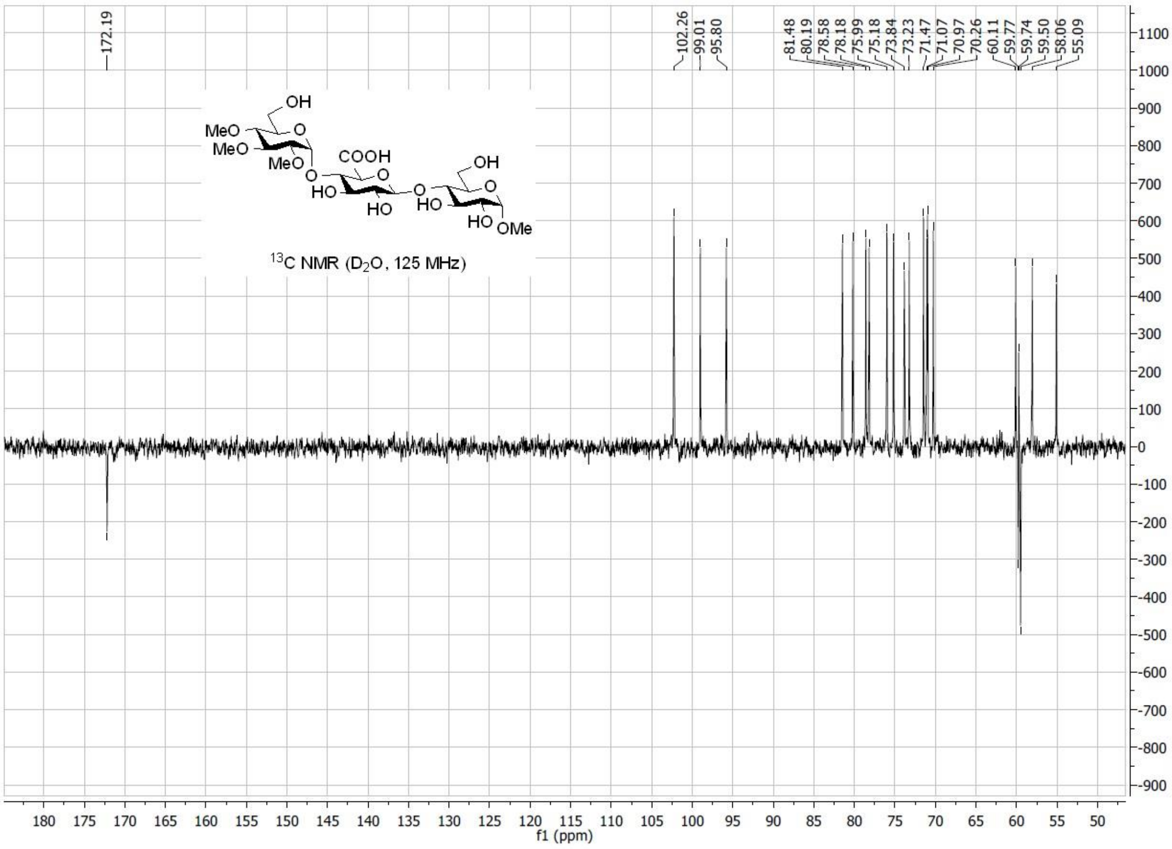
172.19
102.26
99.01
95.80
81.48
80.19
78.58
78.18
75.99
75.18
73.84
73.23
71.47
71.07
70.97
70.26
60.11
59.77
59.74
59.50
58.06
55.09
OH
MeO
MeO
MeO
O
COOH
OH
HO
HO
HO
HO
OMe
13C NMR (D2O, 125 MHz)
f1 (ppm)

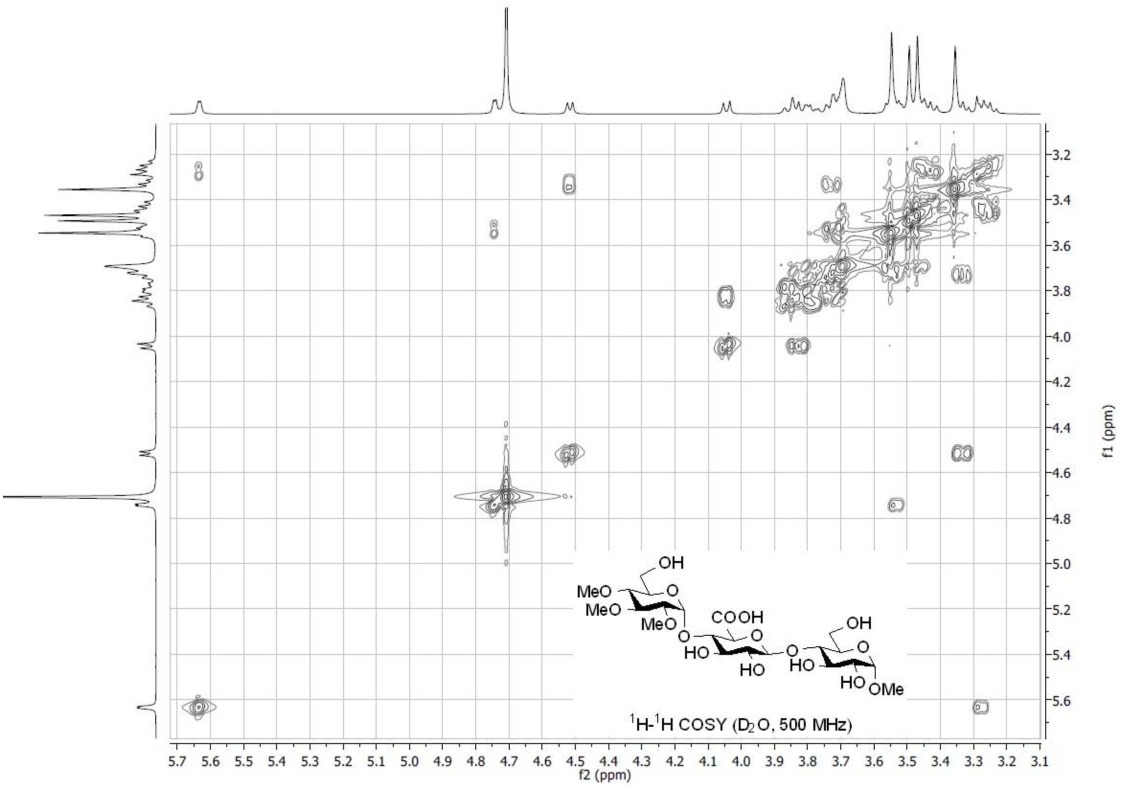

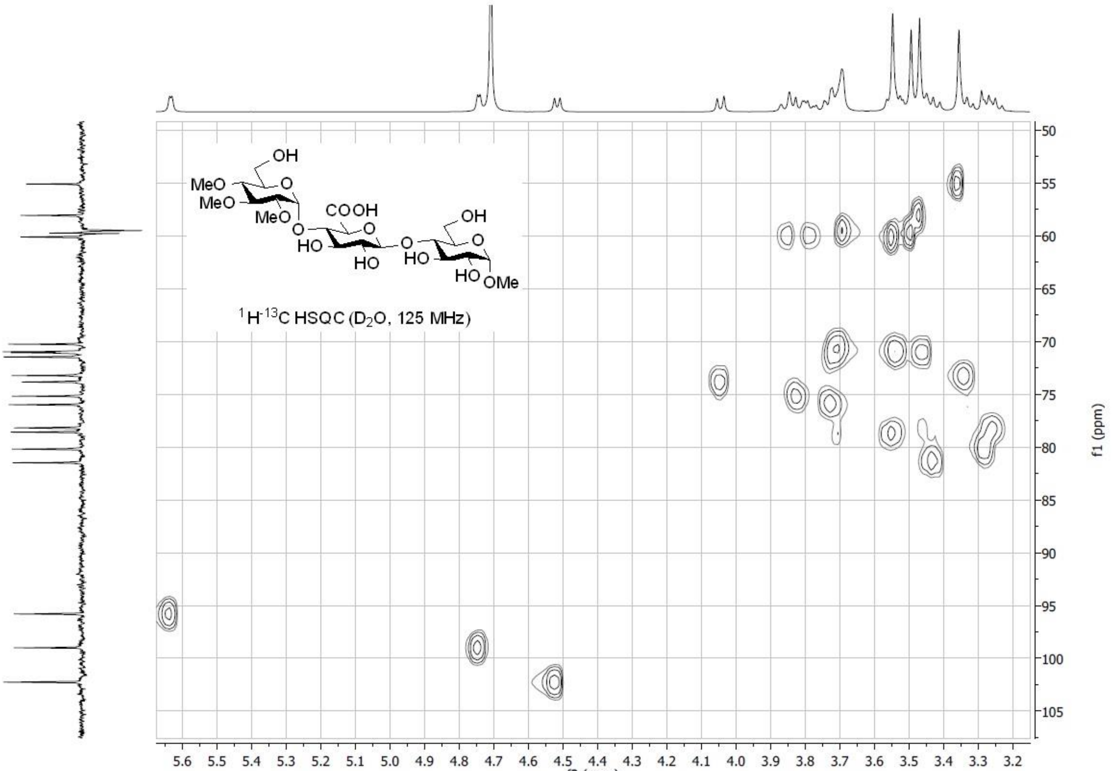

Figure S2. ^{1}H, ^{13}C, COSY and HSQC NMR spectra of compound **10**.

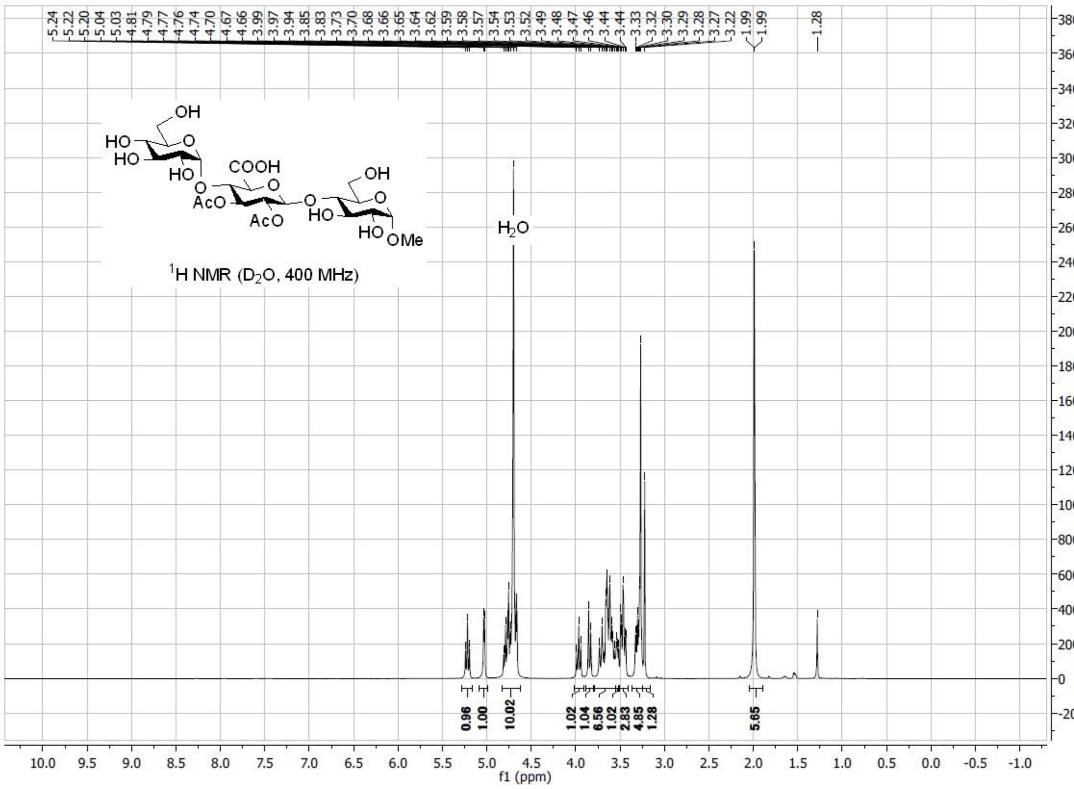

COOH
OH
HO
AcO
OMe
H2O
1H NMR (D2O, 400 MHz)
f1 (ppm)

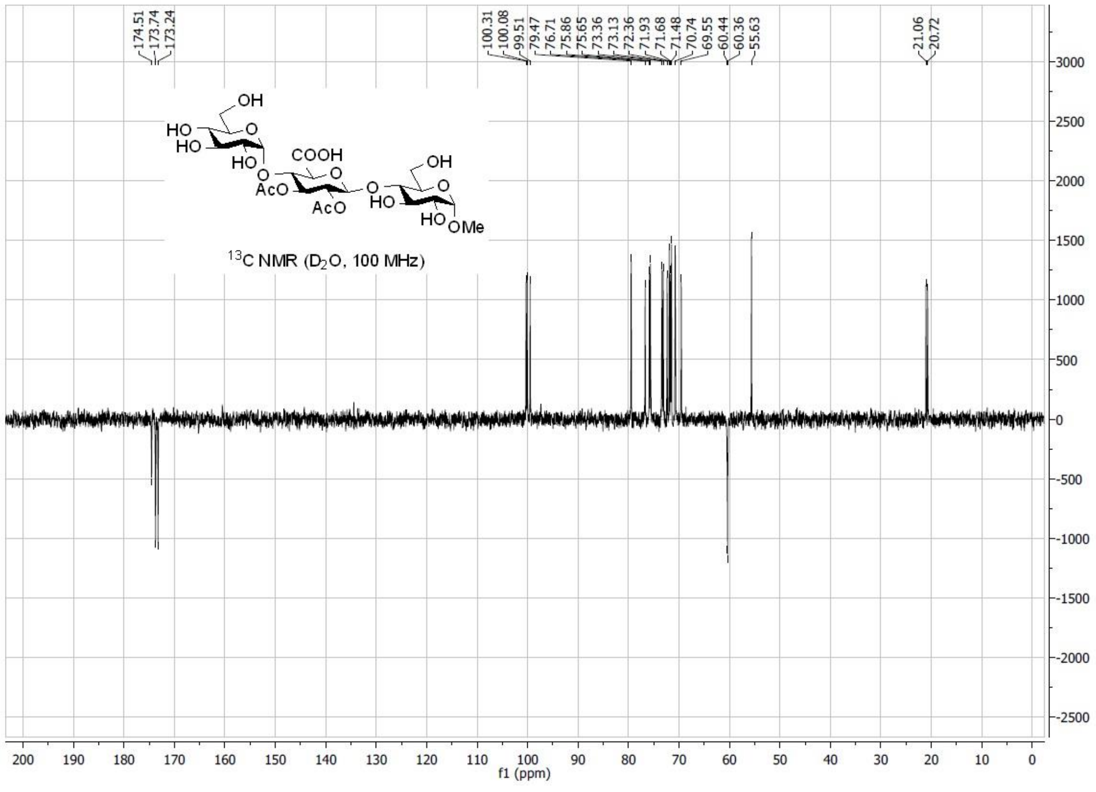

COOH
OH
HO
AcO
OMe
13C NMR (D2O, 100 MHz)
f1 (ppm)

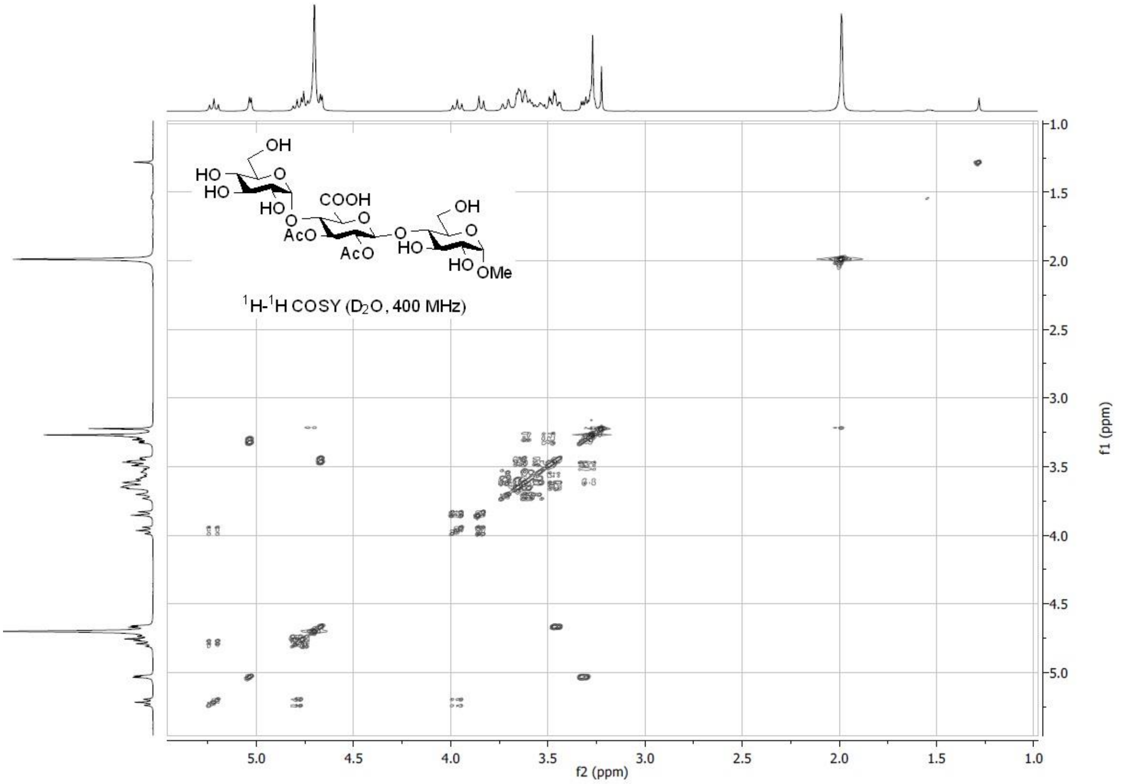

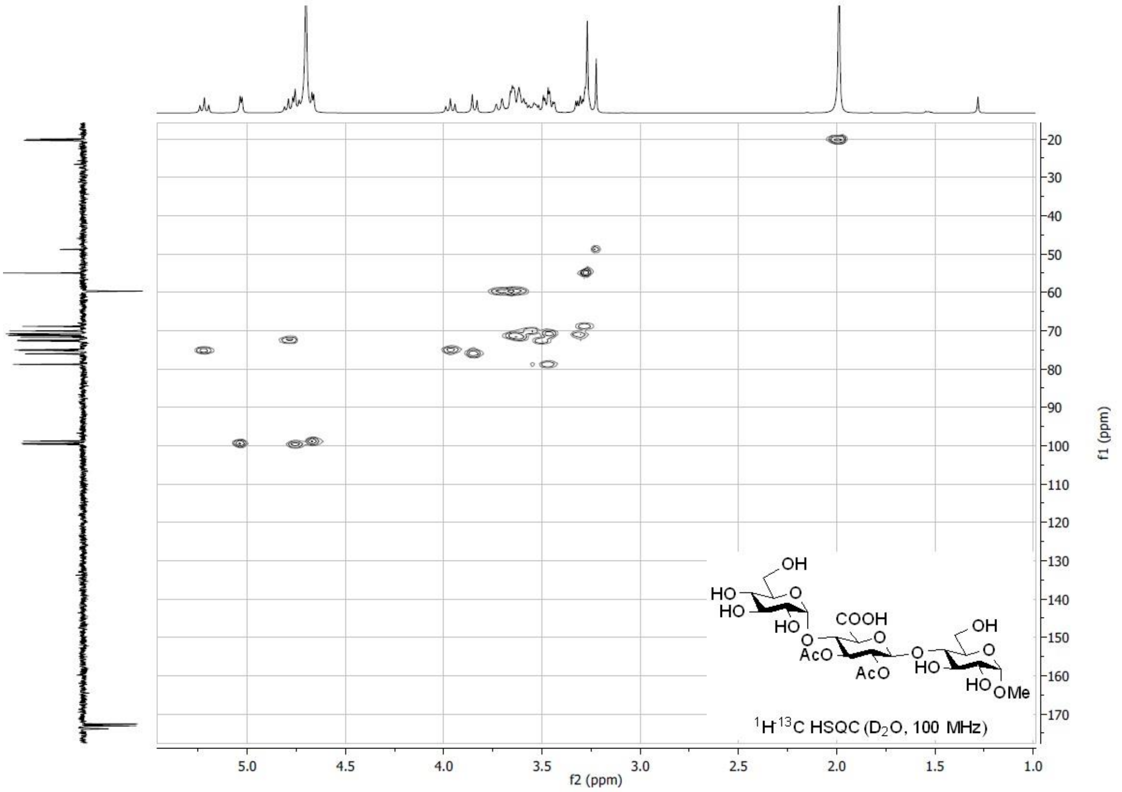

Figure S3. ^{1}H, ^{13}C, COSY and HSQC NMR spectra of compound **11**.

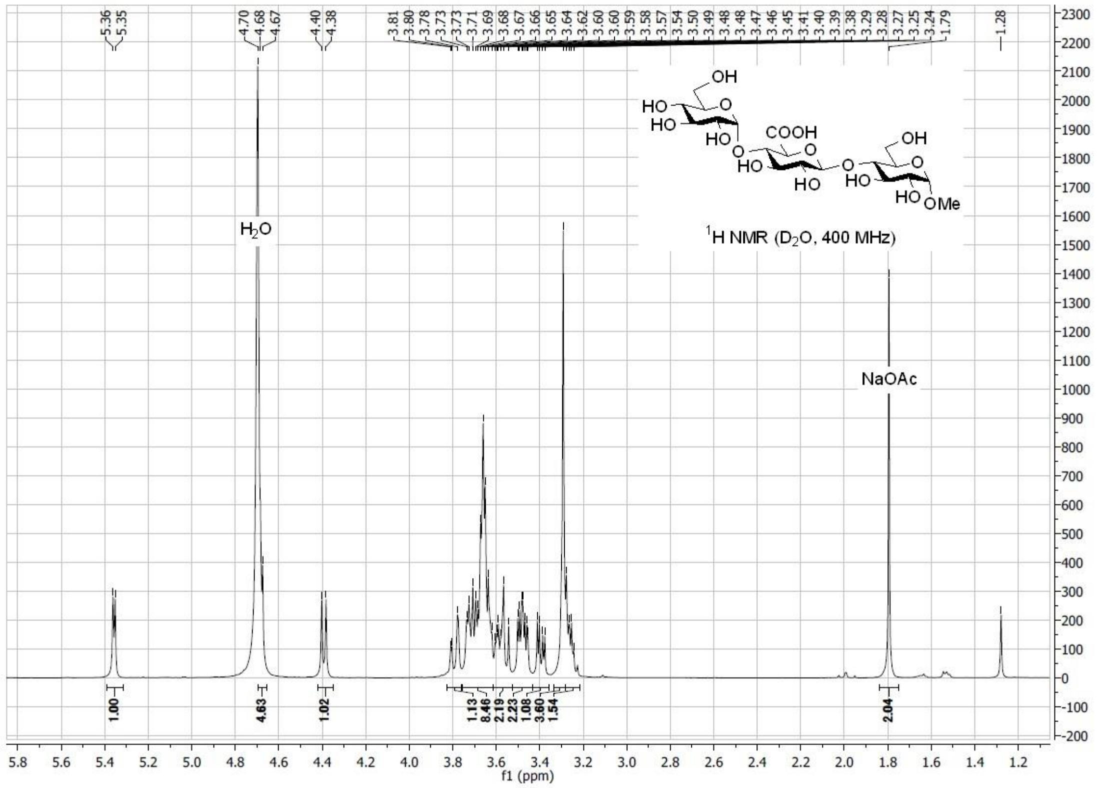
H2O
NaOAc
1H NMR (D2O, 400 MHz)
f1 (ppm)

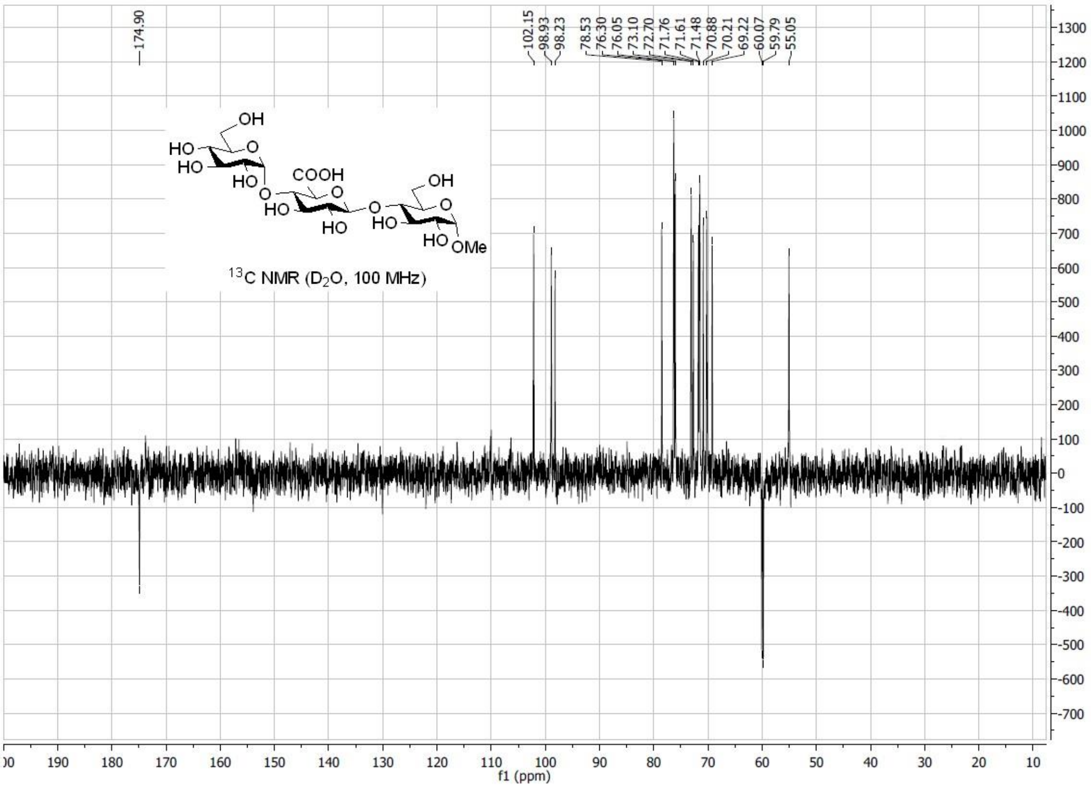
13C NMR (D2O, 100 MHz)
f1 (ppm)

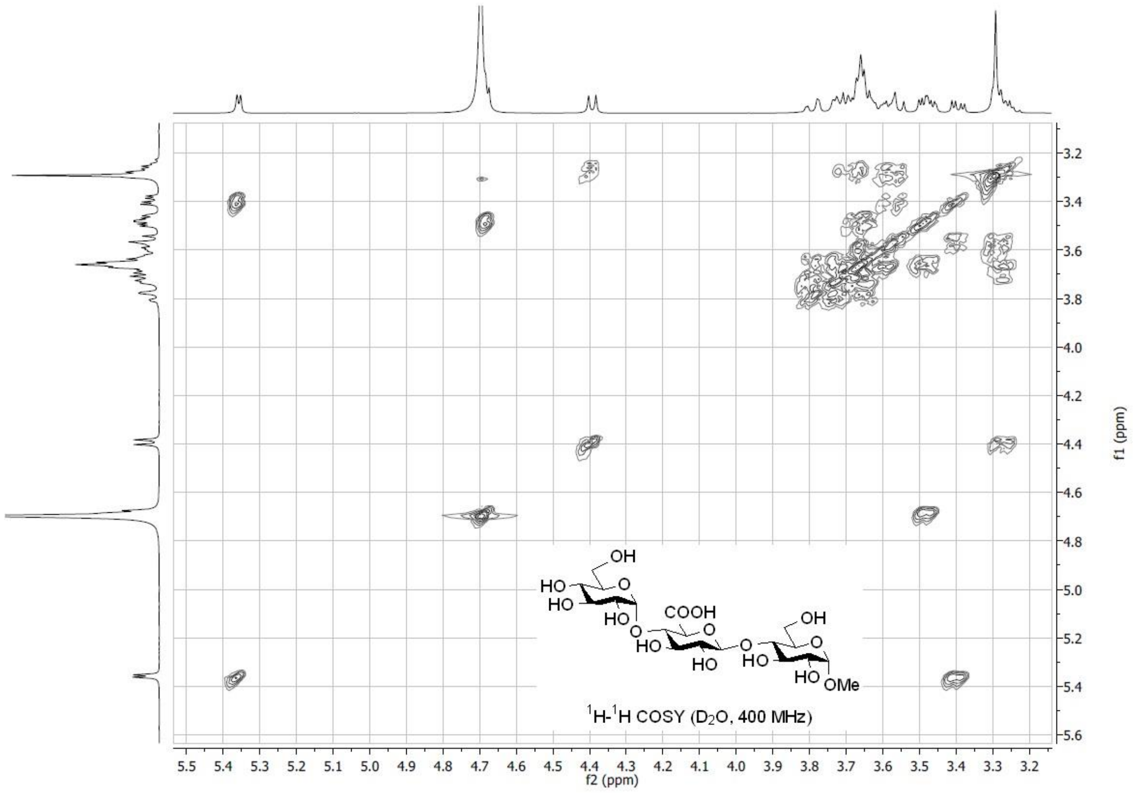

^{1}H-^{1}H COSY (D_2O, 400 MHz)
f2 (ppm)
f1 (ppm)
OH
HO
HO
HO
COOH
HO
HO
OH
HO
HO
OMe

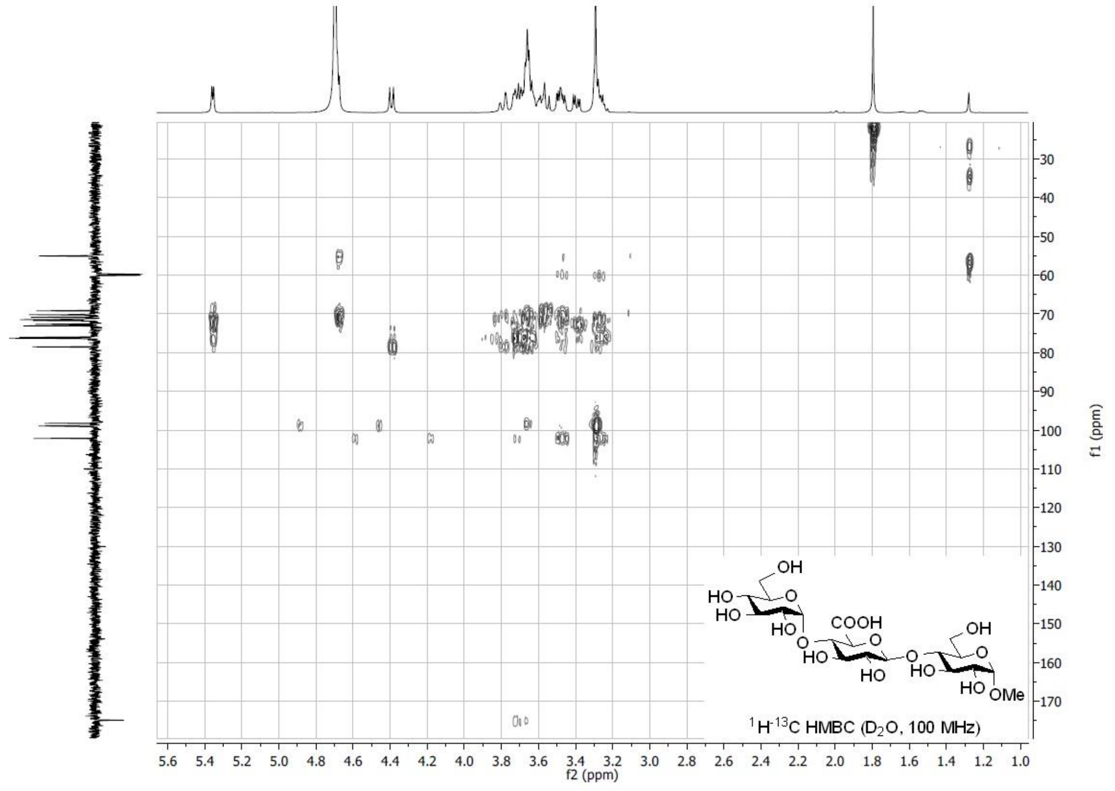

^{1}H-^{13}C HMBC (D_2O, 100 MHz)
f2 (ppm)
f1 (ppm)
OH
HO
HO
HO
COOH
HO
HO
OH
HO
HO
OMe

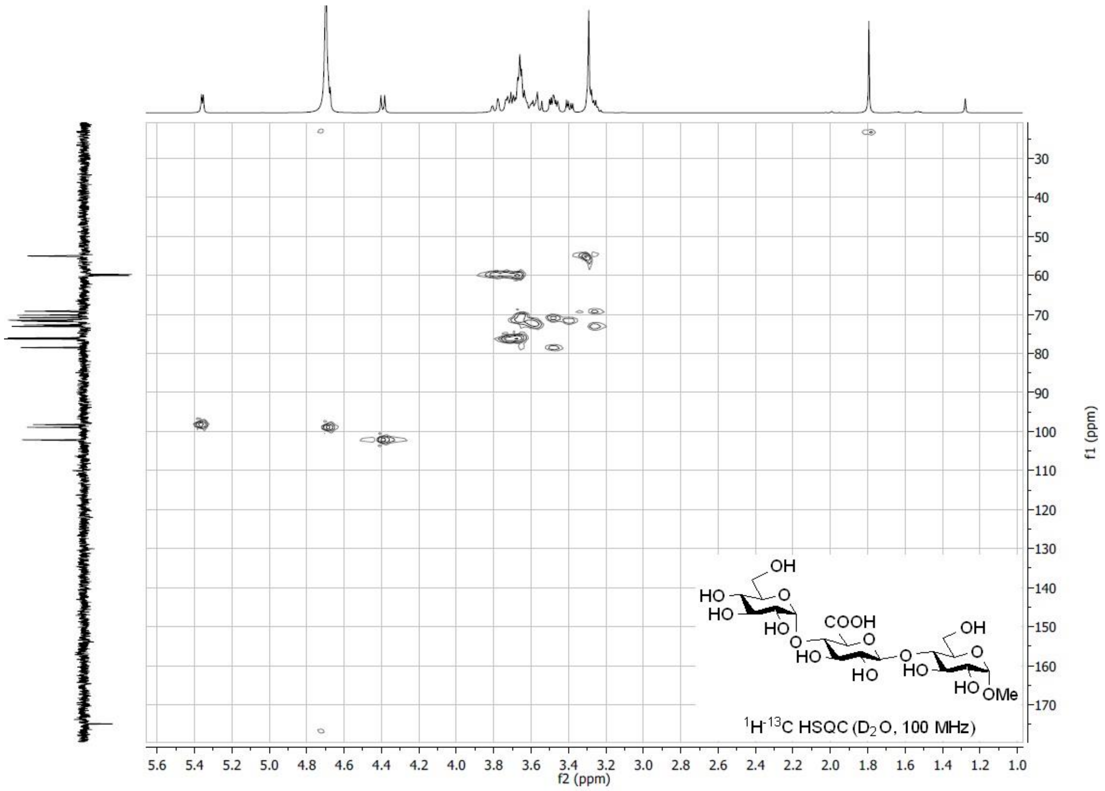

Figure S4. ^{1}H, ^{13}C, COSY, HMBC and HSQC NMR spectra of compound **12**.

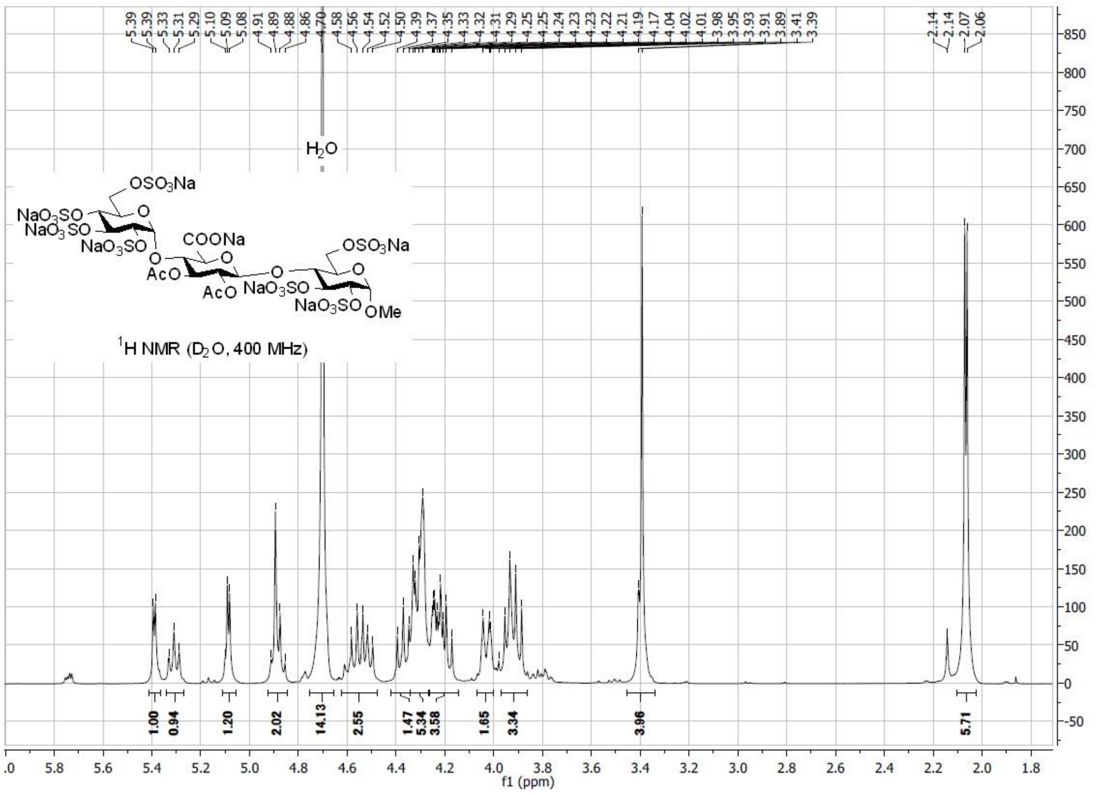

H2O
OSO3Na
NaO3SO
NaO3SO
NaO3SO
COONa
AcO
AcO
NaO3SO
OSO3Na
NaO3SO
OMe
1H NMR (D2O, 400 MHz)
f1 (ppm)

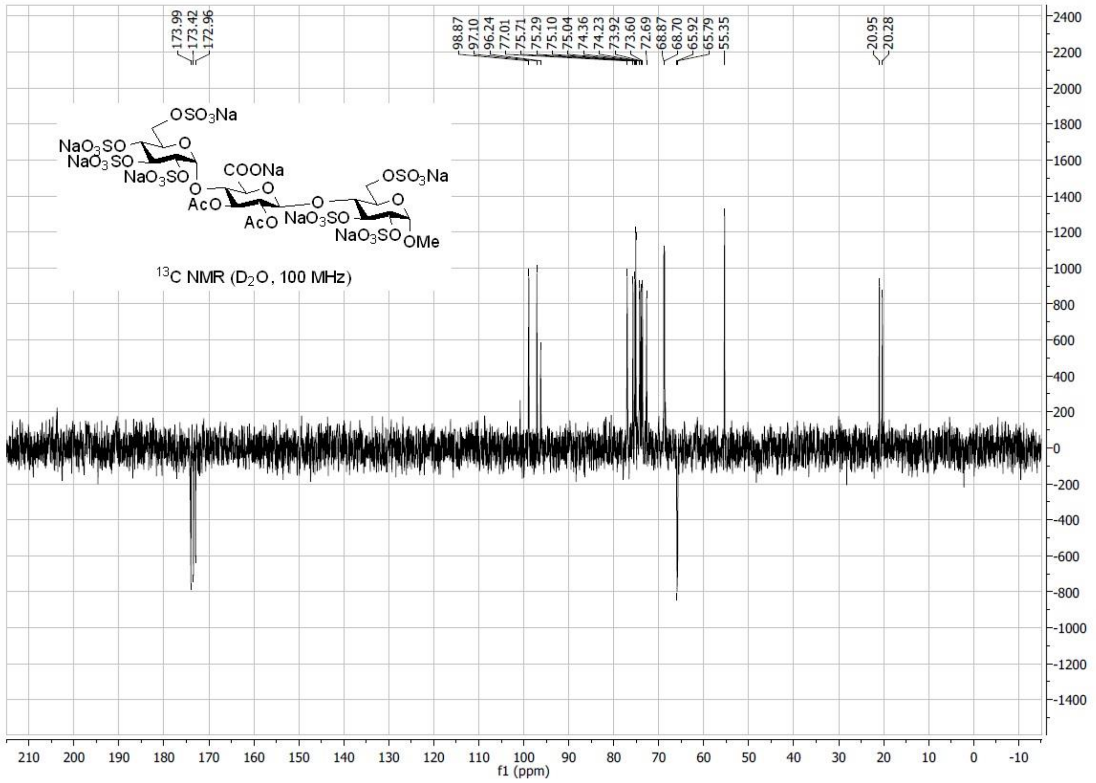

OSO3Na
NaO3SO
NaO3SO
NaO3SO
COONa
AcO
AcO
NaO3SO
OSO3Na
NaO3SO
OMe
13C NMR (D2O, 100 MHz)
f1 (ppm)

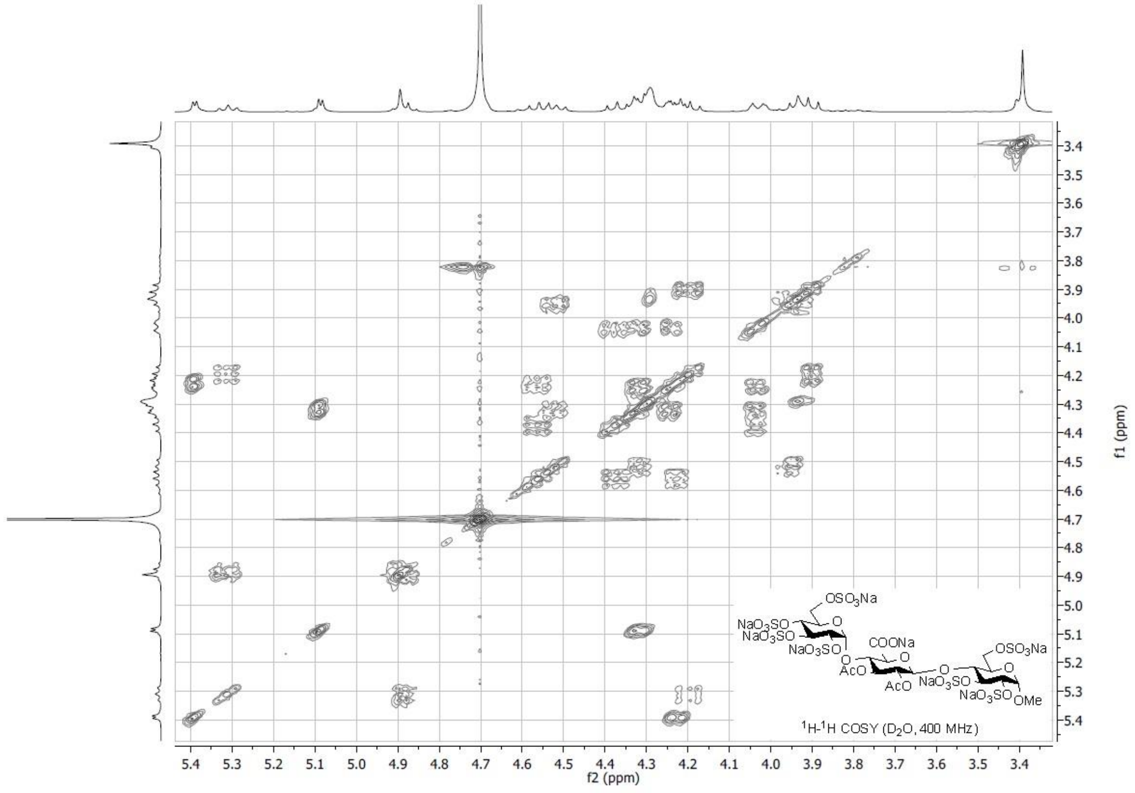

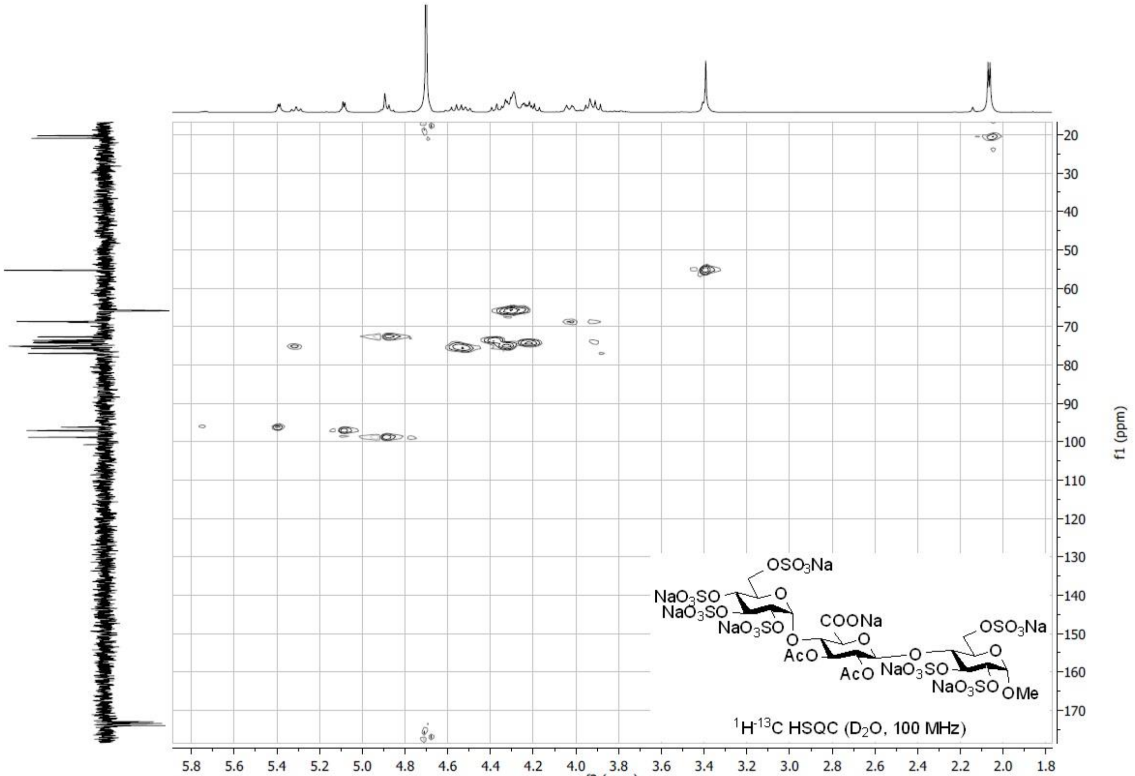

Figure S5. ^{1}H, ^{13}C, COSY and HSQC NMR spectra of compound **13**.

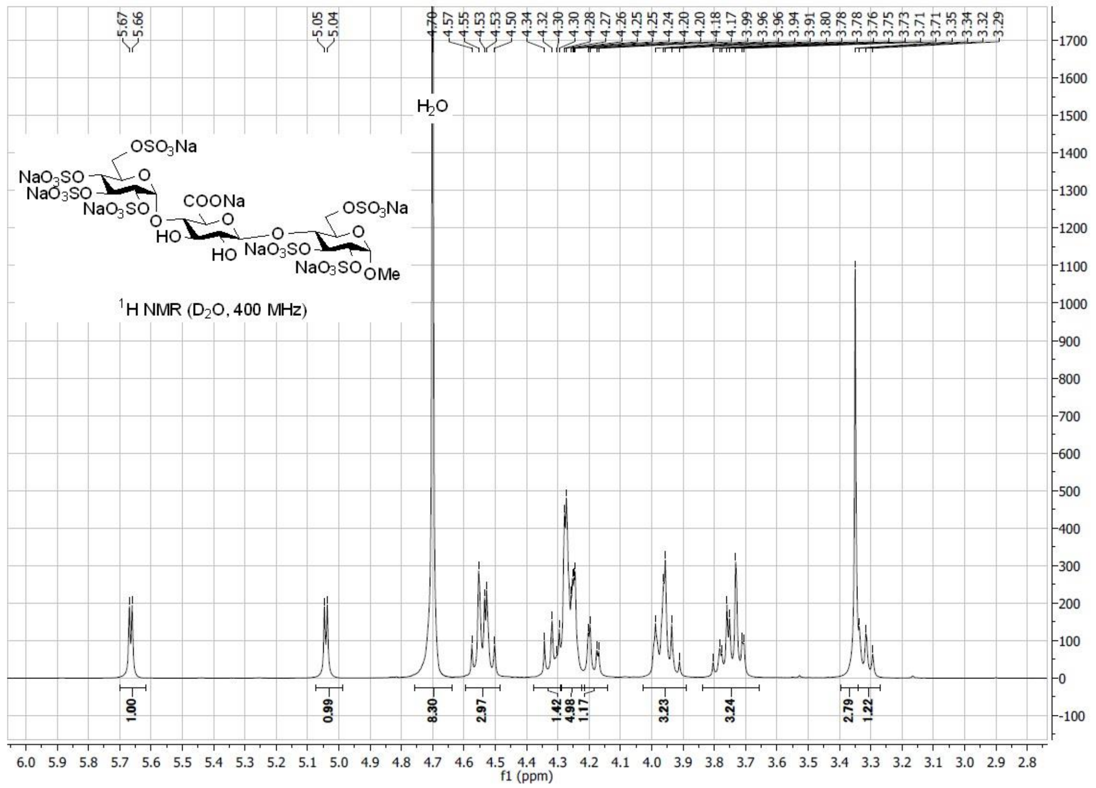

H$_2$O
OSO$_3$Na
NaO$_3$SO
NaO$_3$SO
NaO$_3$SO
COONa
OSO$_3$Na
HO
HO
NaO$_3$SO
NaO$_3$SO
OMe
^{1}H NMR (D$_2$O, 400 MHz)
f1 (ppm)

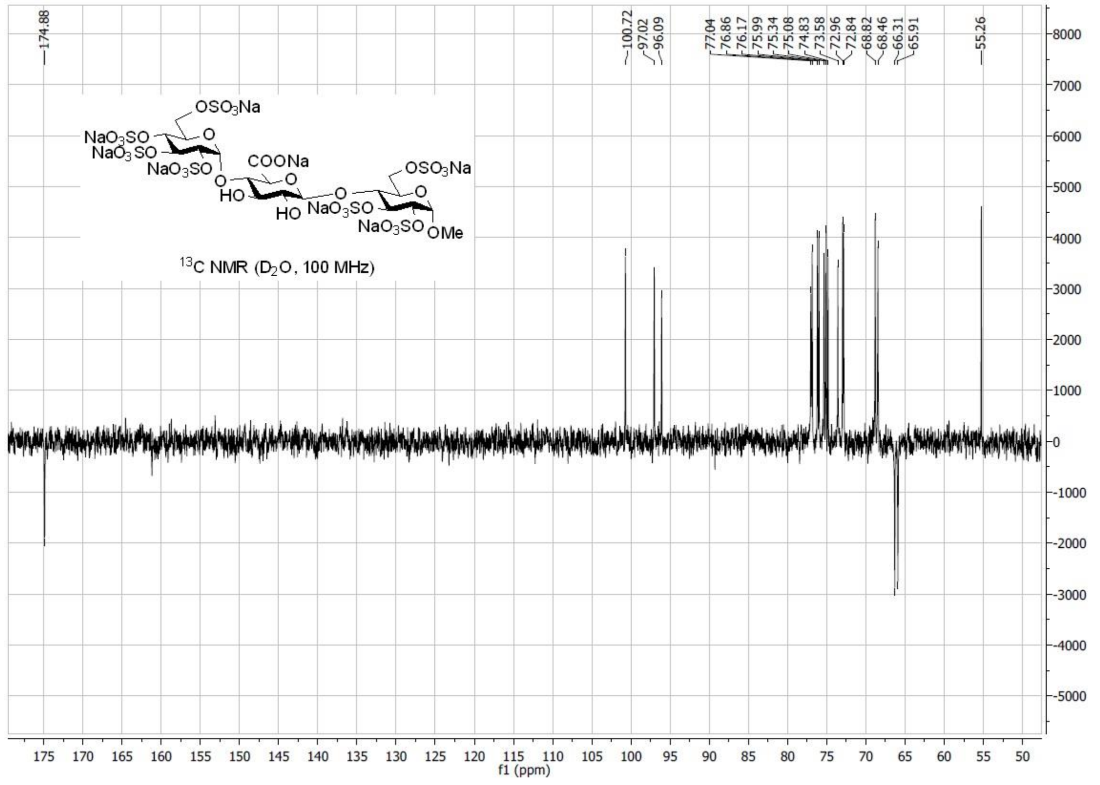

OSO$_3$Na
NaO$_3$SO
NaO$_3$SO
NaO$_3$SO
COONa
OSO$_3$Na
HO
HO
NaO$_3$SO
NaO$_3$SO
OMe
^{13}C NMR (D$_2$O, 100 MHz)
f1 (ppm)

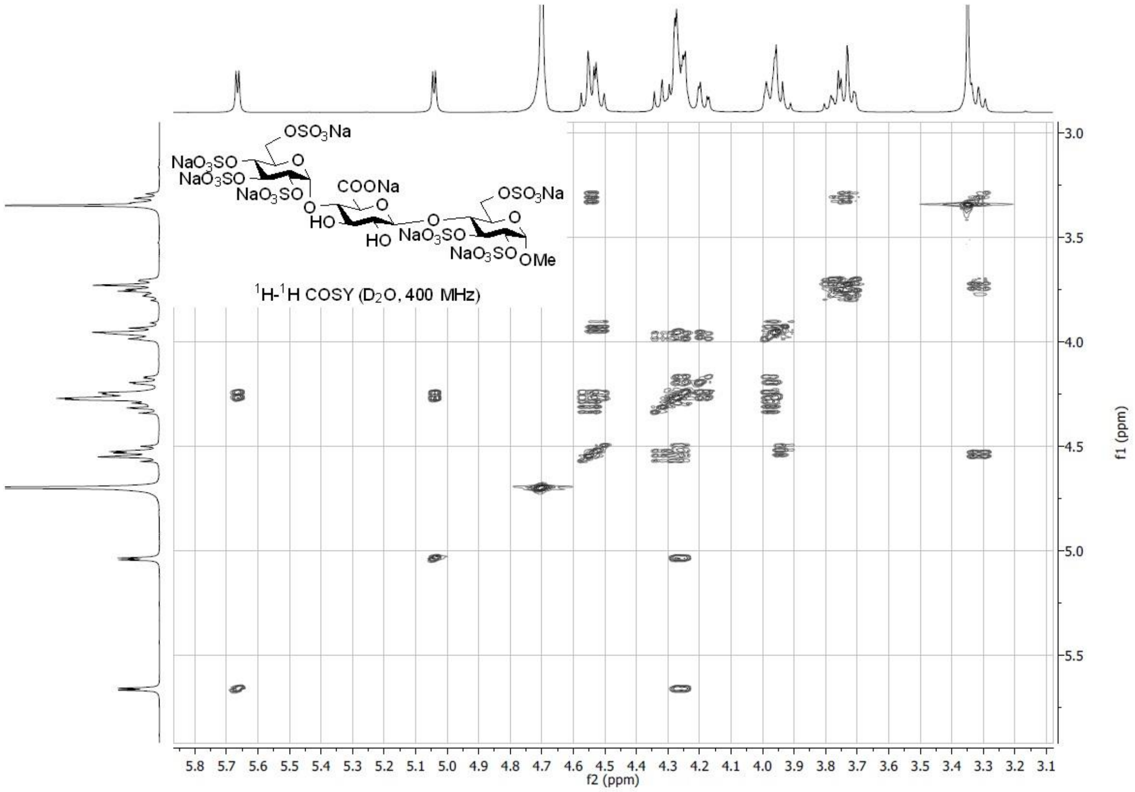
OSO3Na
NaO3SO
NaO3SO
NaO3SO
COONa
HO
HO
NaO3SO
OSO3Na
NaO3SO
OMe
1H-1H COSY (D2O, 400 MHz)
f1 (ppm)
f2 (ppm)

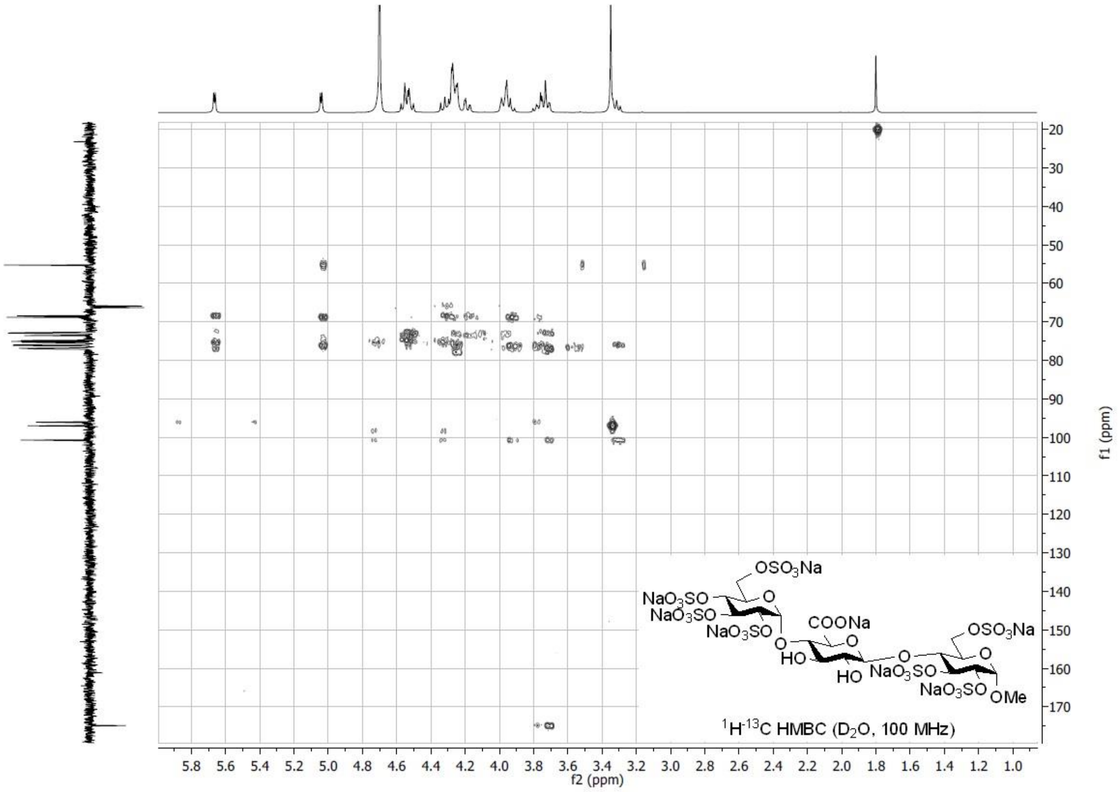
OSO3Na
NaO3SO
NaO3SO
NaO3SO
COONa
HO
HO
NaO3SO
OSO3Na
NaO3SO
OMe
1H-13C HMBC (D2O, 100 MHz)
f1 (ppm)
f2 (ppm)

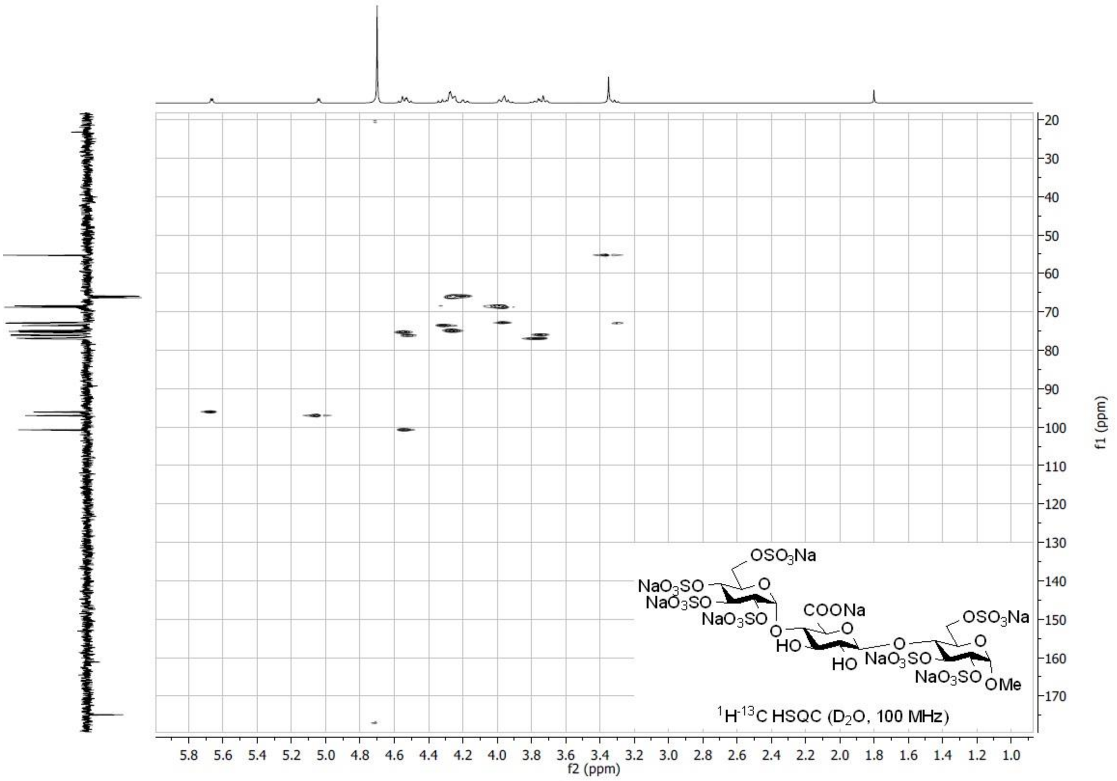

Figure S6. ^1H, ^{13}C, COSY, HMBC and HSQC NMR spectra of compound **14**.

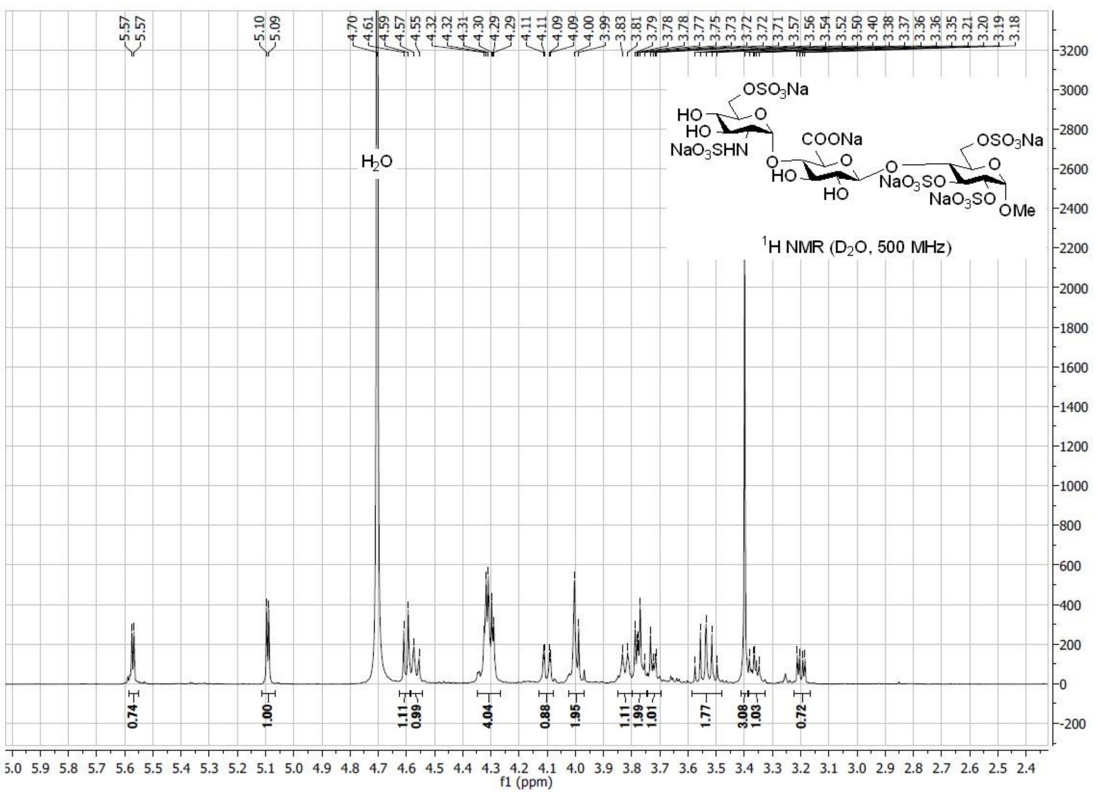
H$_2$O
^{1}H NMR (D$_2$O, 500 MHz)
f1 (ppm)

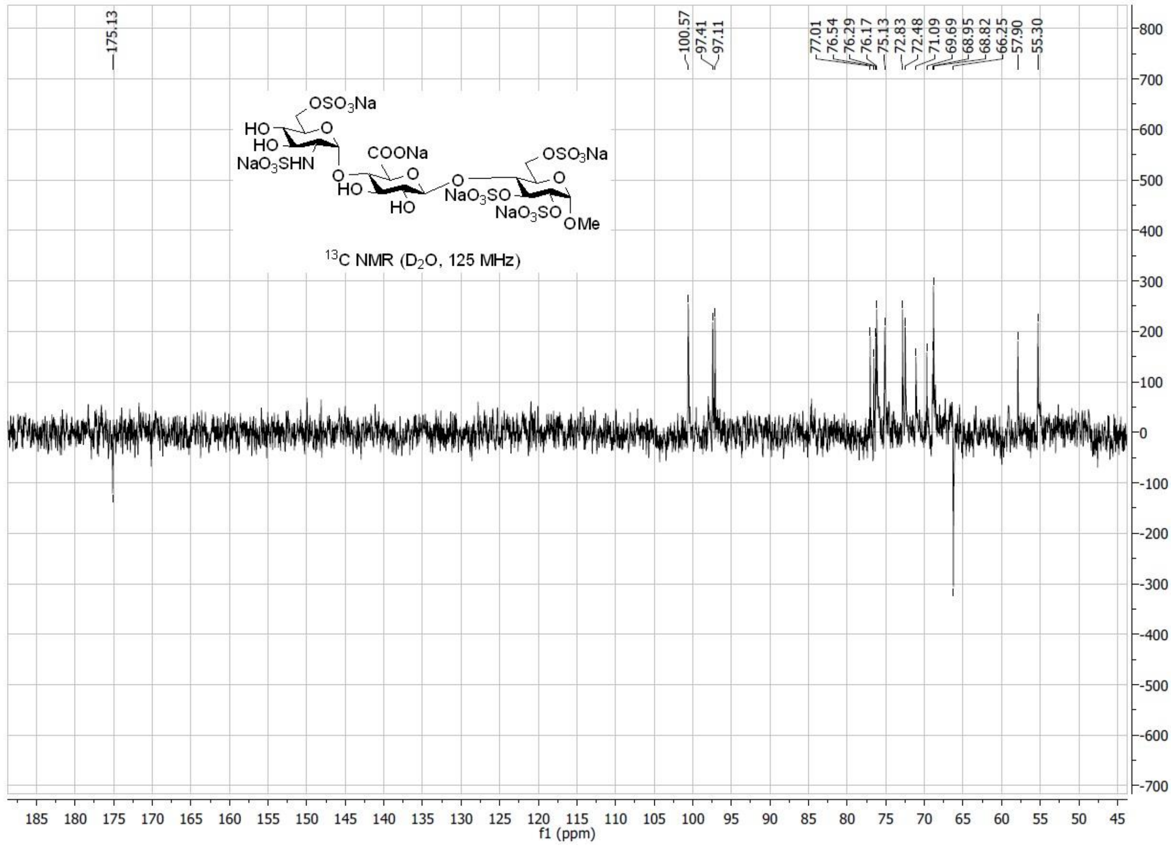
^{13}C NMR (D$_2$O, 125 MHz)
f1 (ppm)

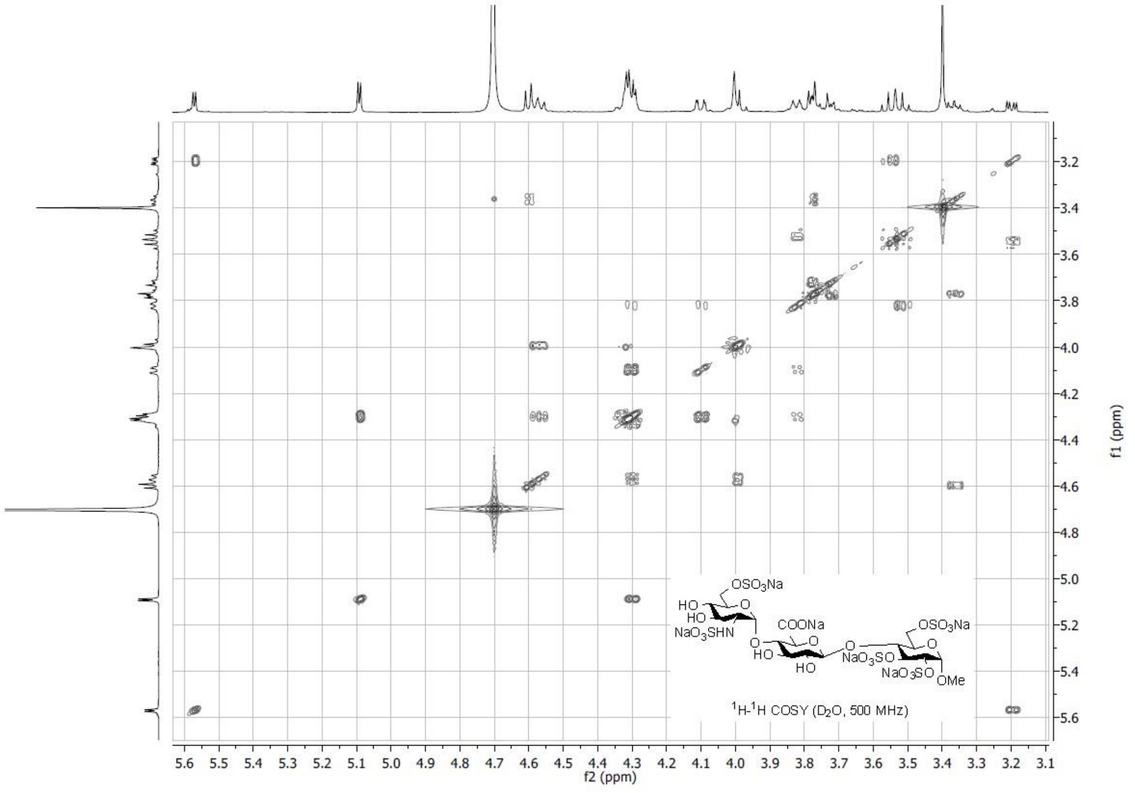

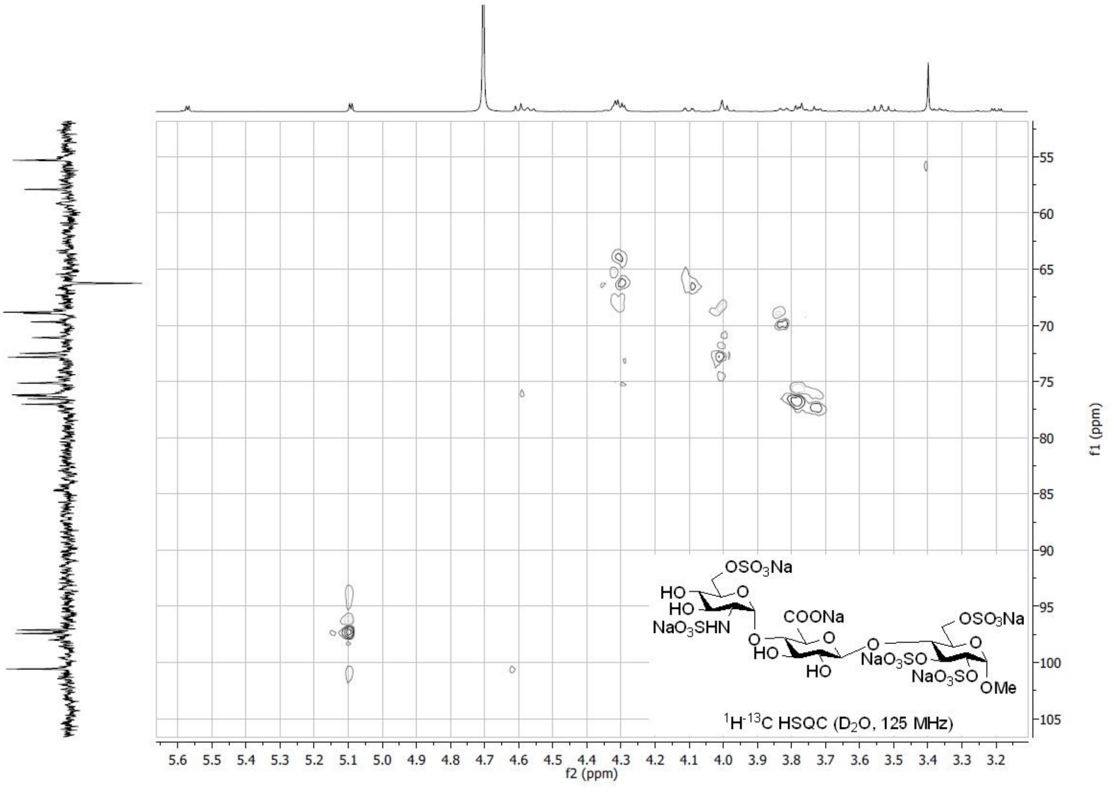

Figure S7. ^{1}H, ^{13}C, COSY and HSQC NMR spectra of compound **15**.

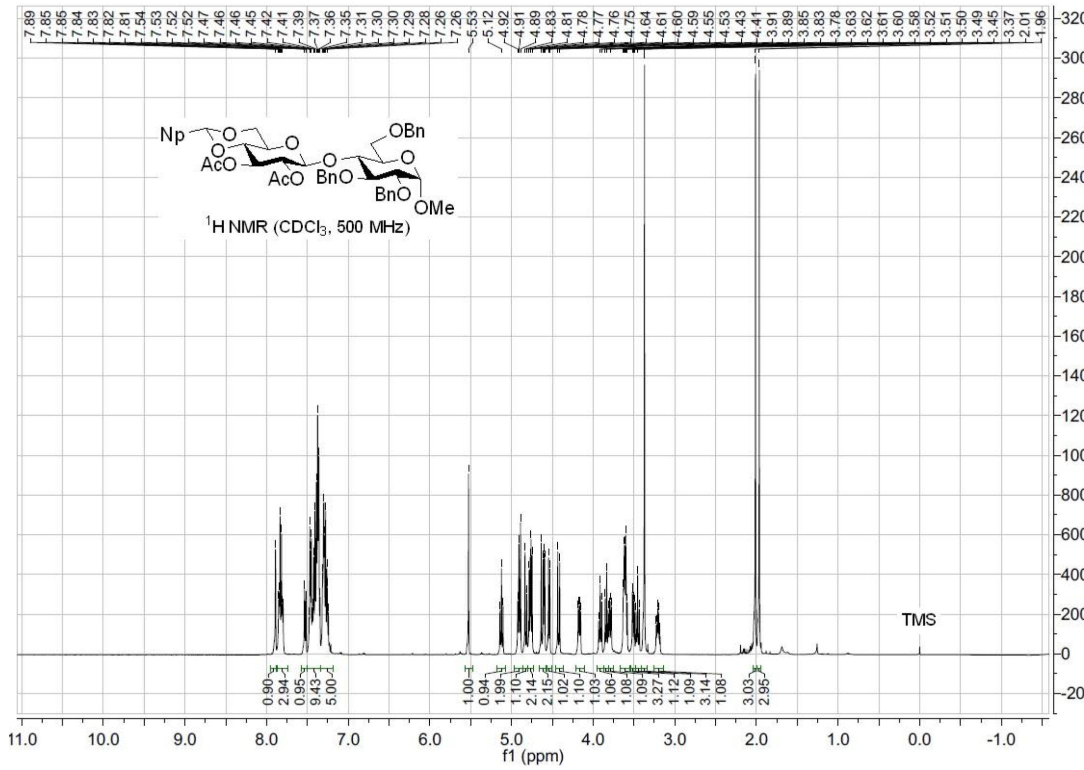

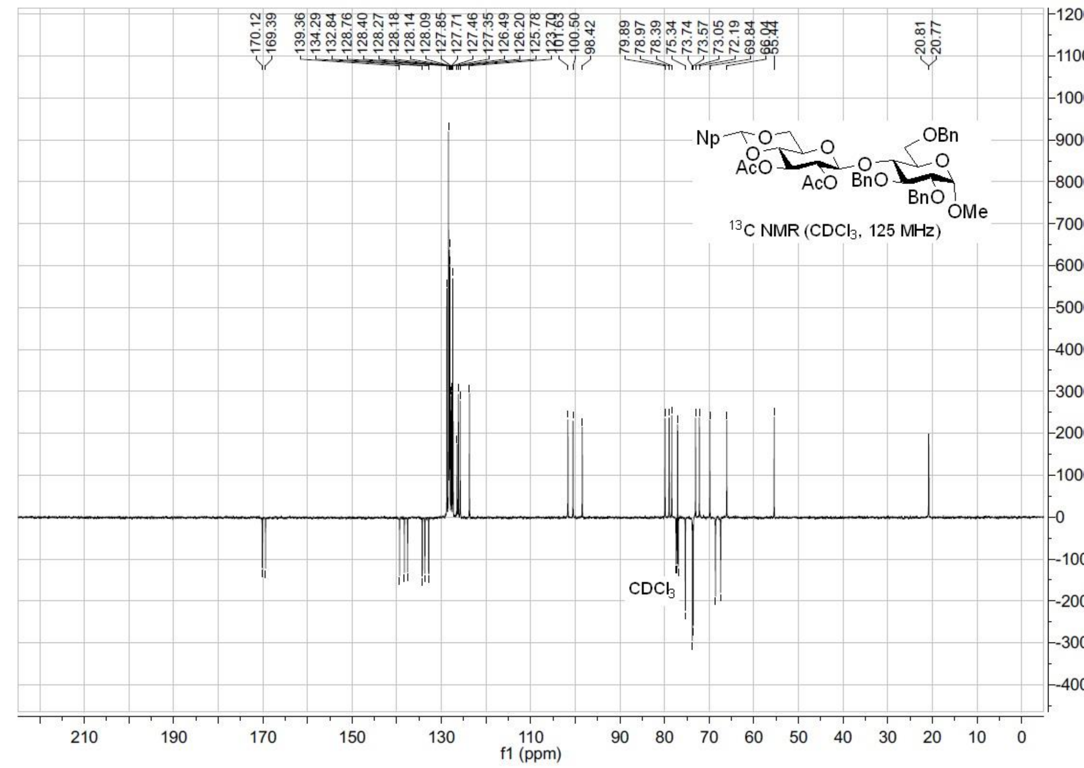

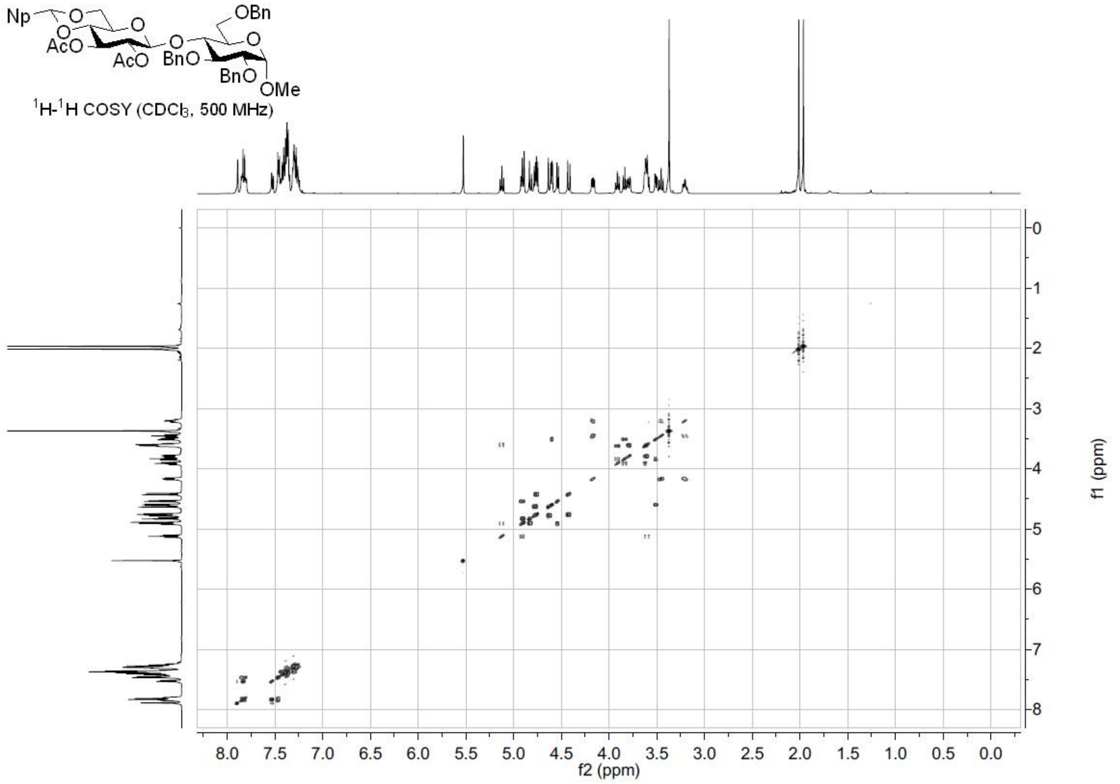

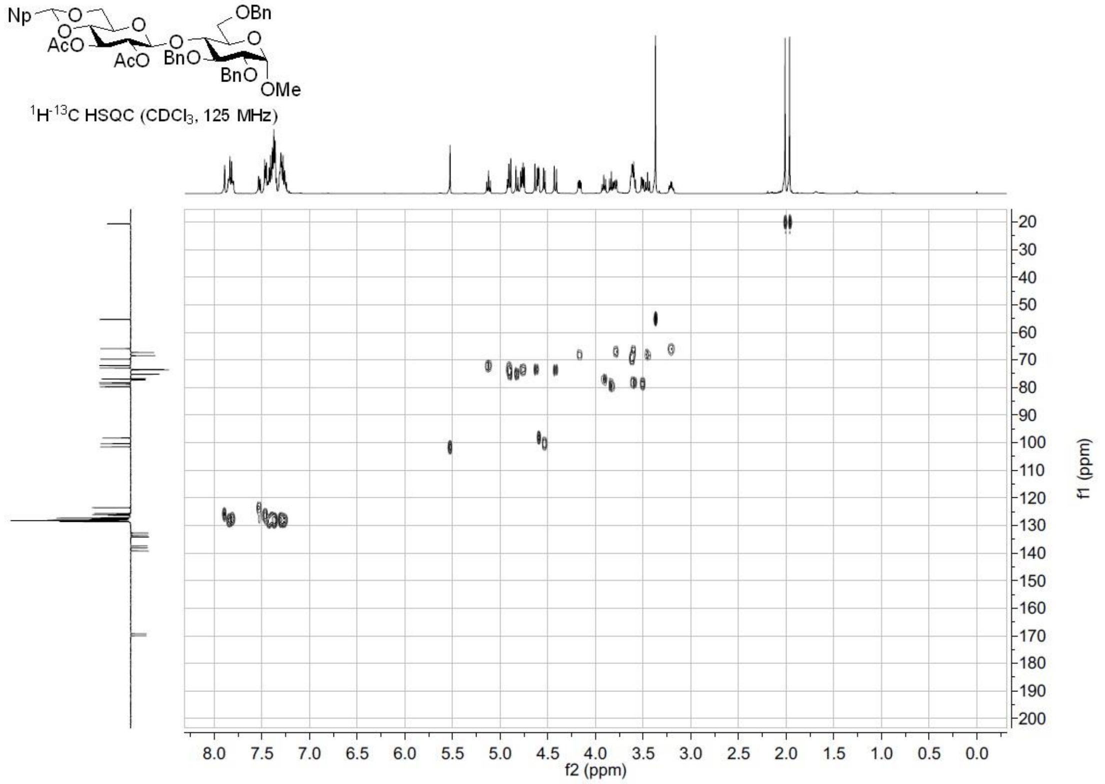

Figure S8. ^{1}H, ^{13}C, COSY and HSQC NMR spectra of compound **18**.

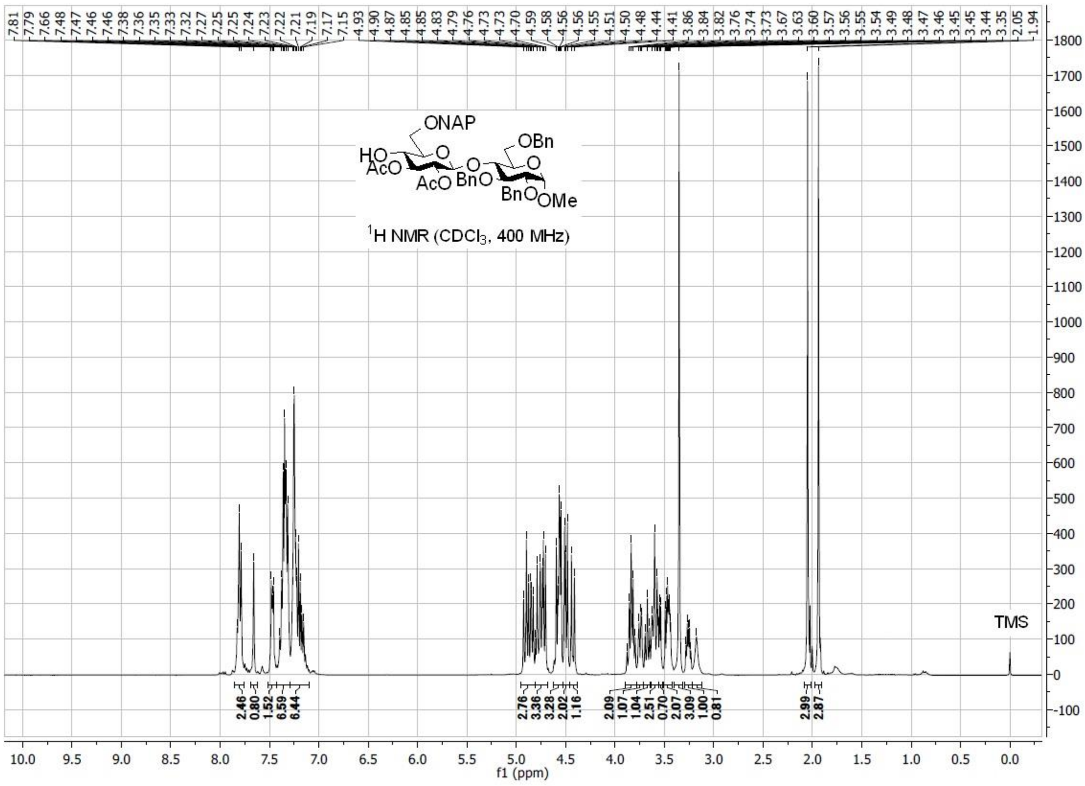
ONAP
OBn
HO
AcO
AcO
BnO
BnO
OMe
^{1}H NMR (CDCl3, 400 MHz)
TMS
f1 (ppm)

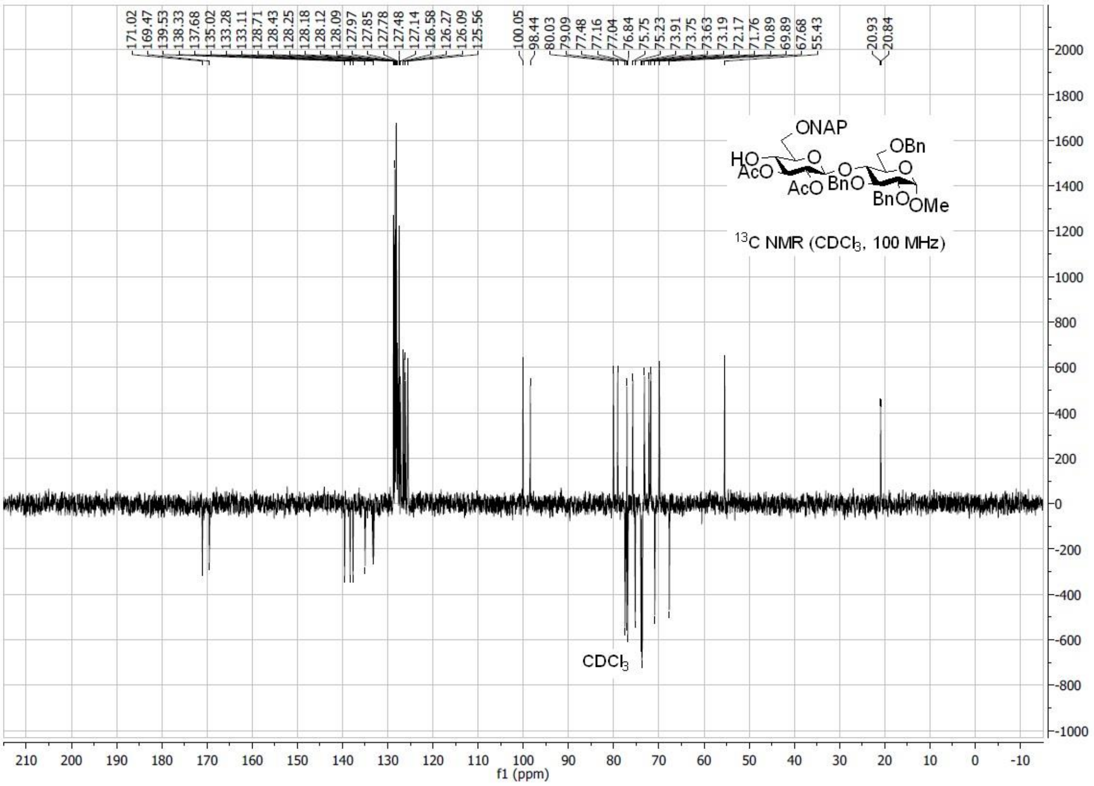
ONAP
OBn
HO
AcO
AcO
BnO
BnO
OMe
^{13}C NMR (CDCl3, 100 MHz)
CDCl3
f1 (ppm)

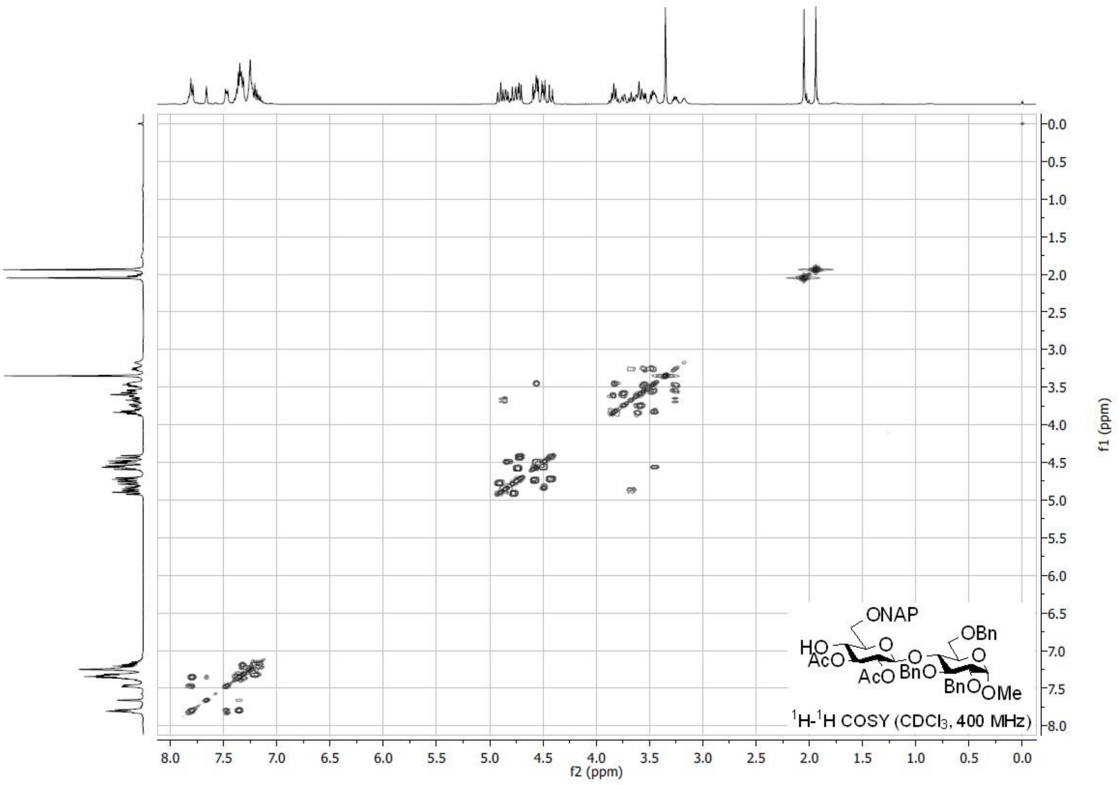

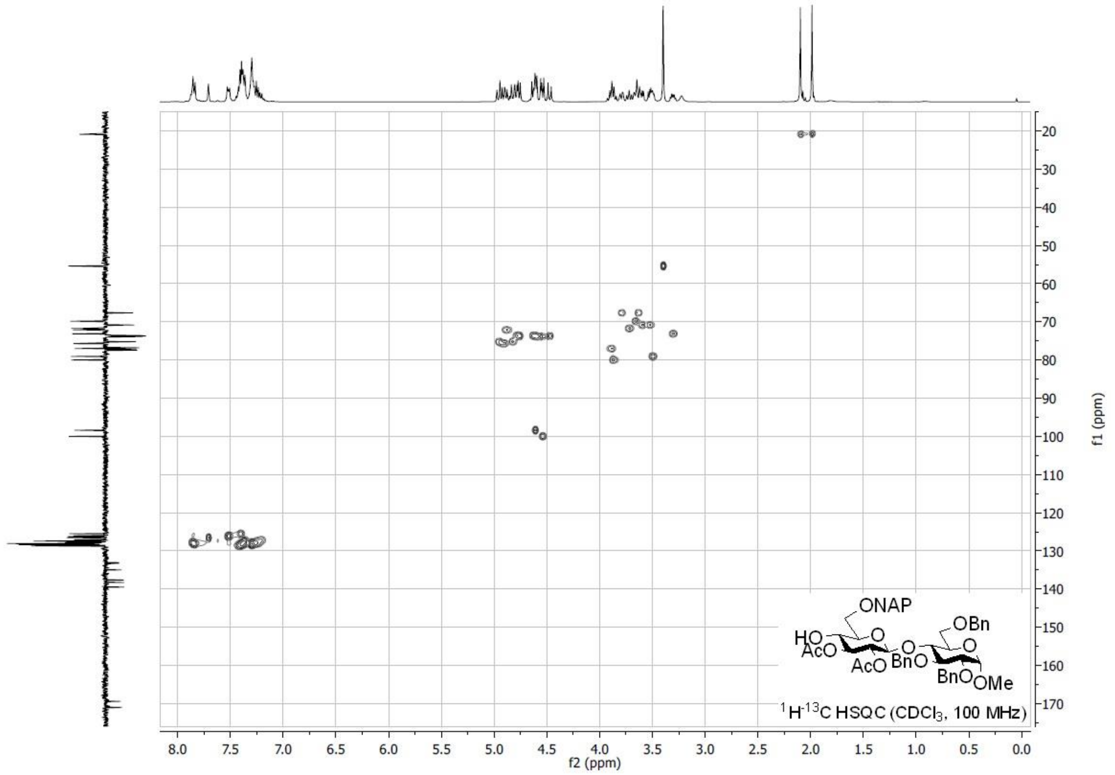

Figure S9. ^{1}H, ^{13}C, COSY and HSQC NMR spectra of compound **19**.

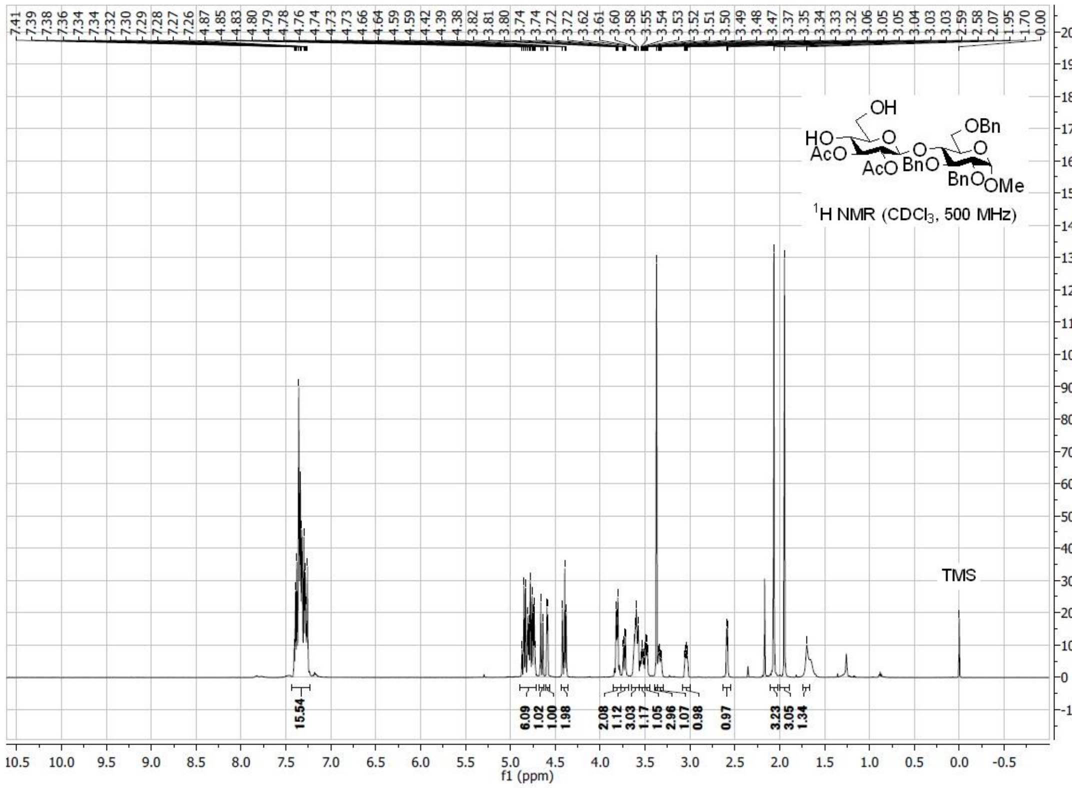

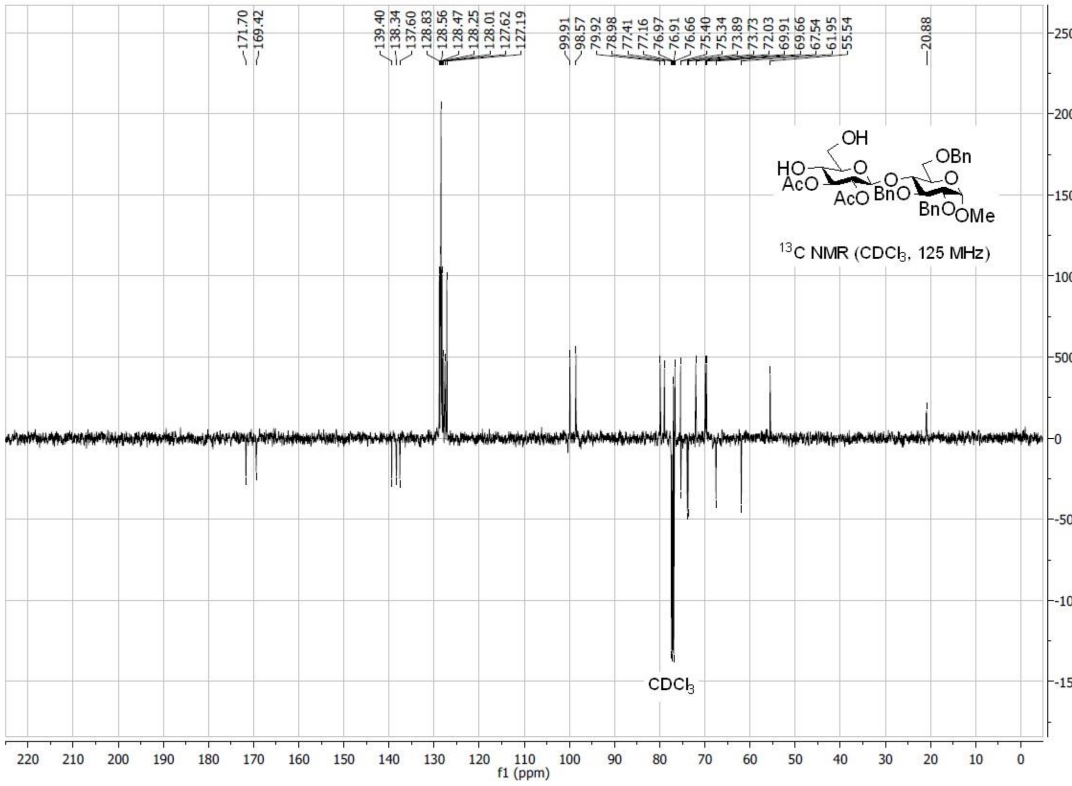

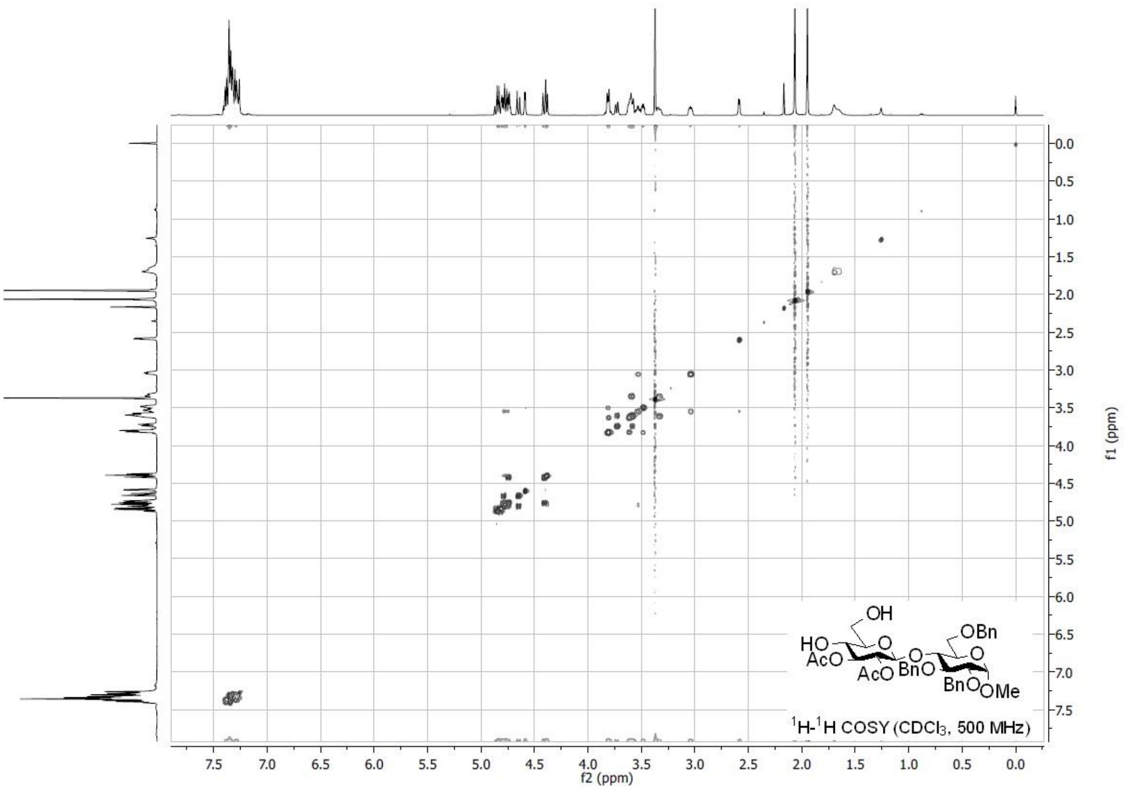

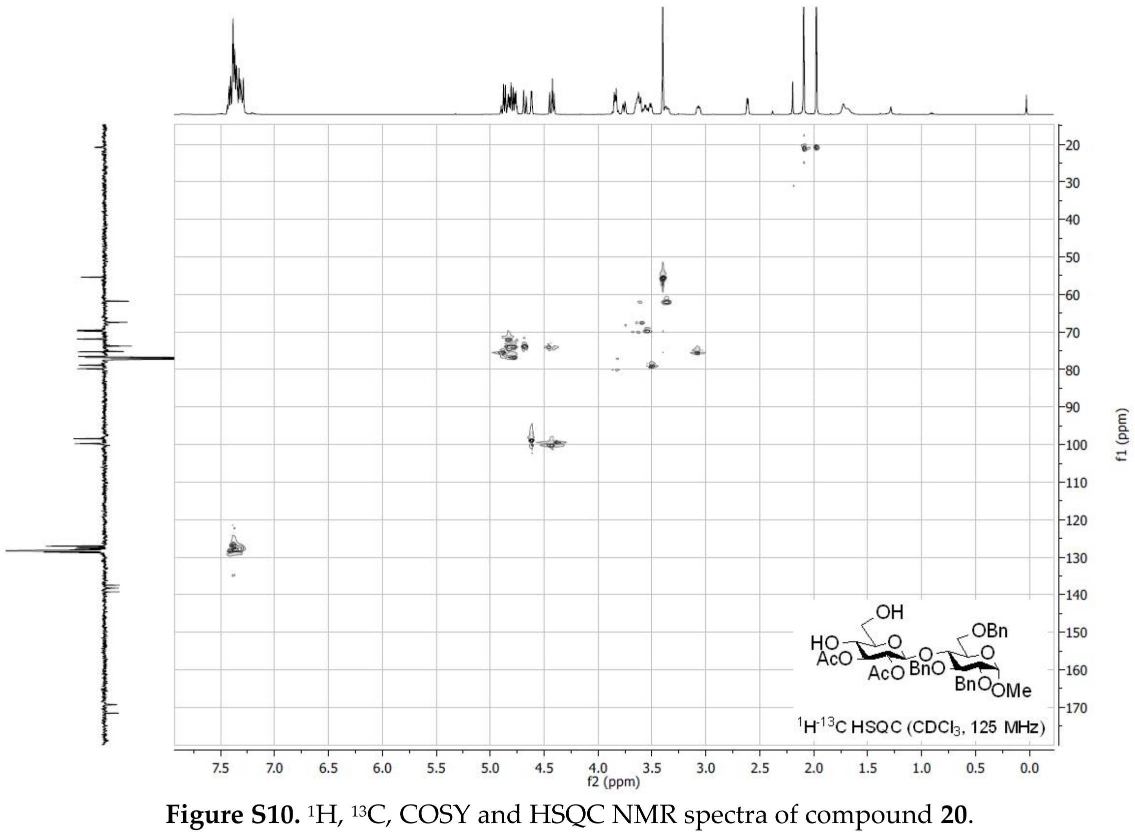

Figure S10. ^{1}H, ^{13}C, COSY and HSQC NMR spectra of compound **20**.

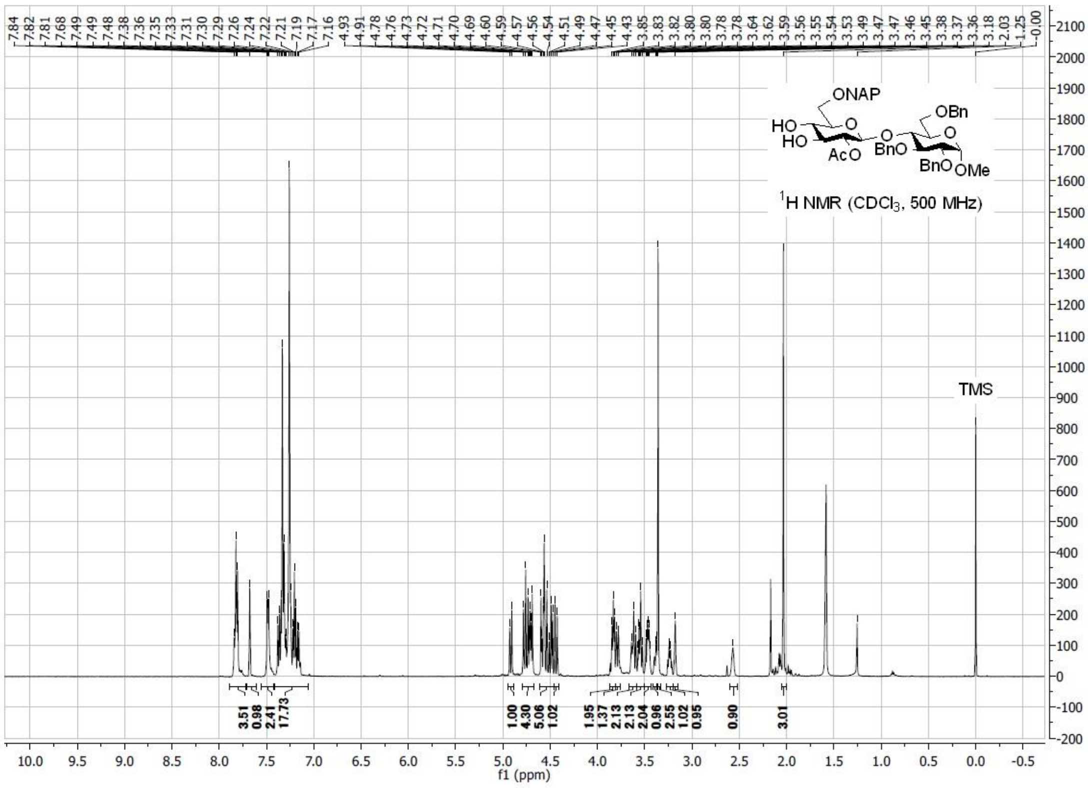
ONAP
HO
HO
AcO
BnO
OBn
BnO
OMe
^{1}H NMR (CDCl$_3$, 500 MHz)
TMS
f1 (ppm)

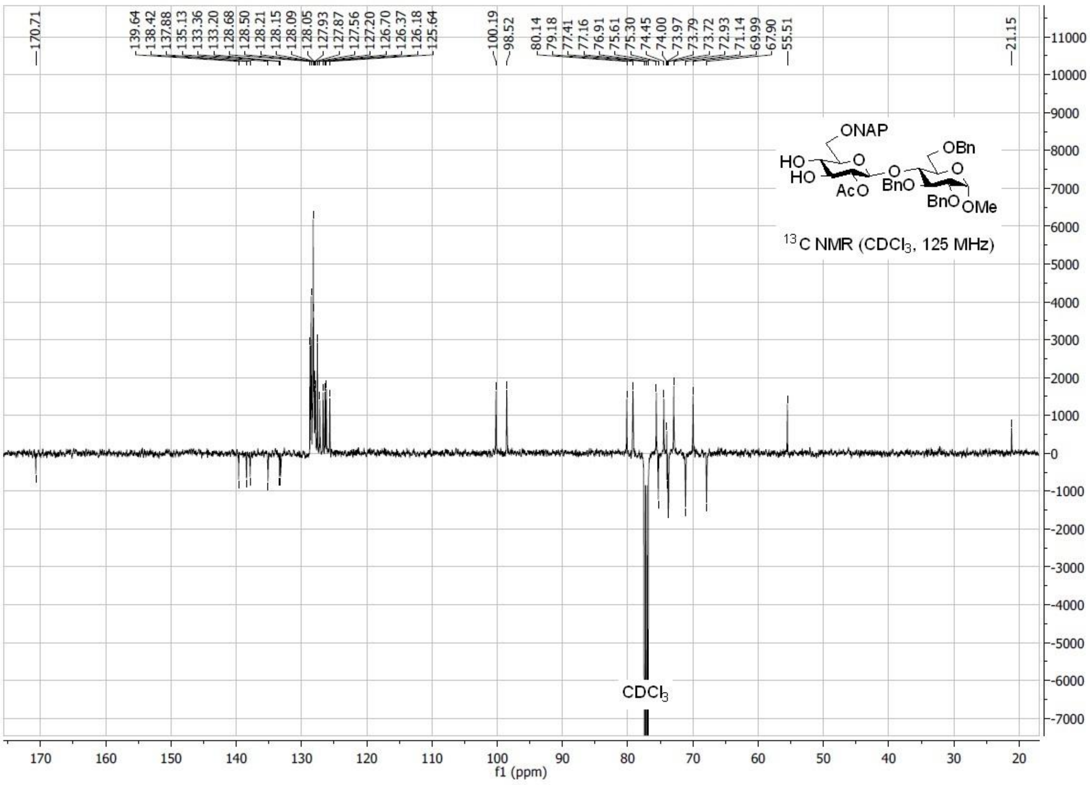
ONAP
HO
HO
AcO
BnO
OBn
BnO
OMe
^{13}C NMR (CDCl$_3$, 125 MHz)
CDCl$_3$
f1 (ppm)

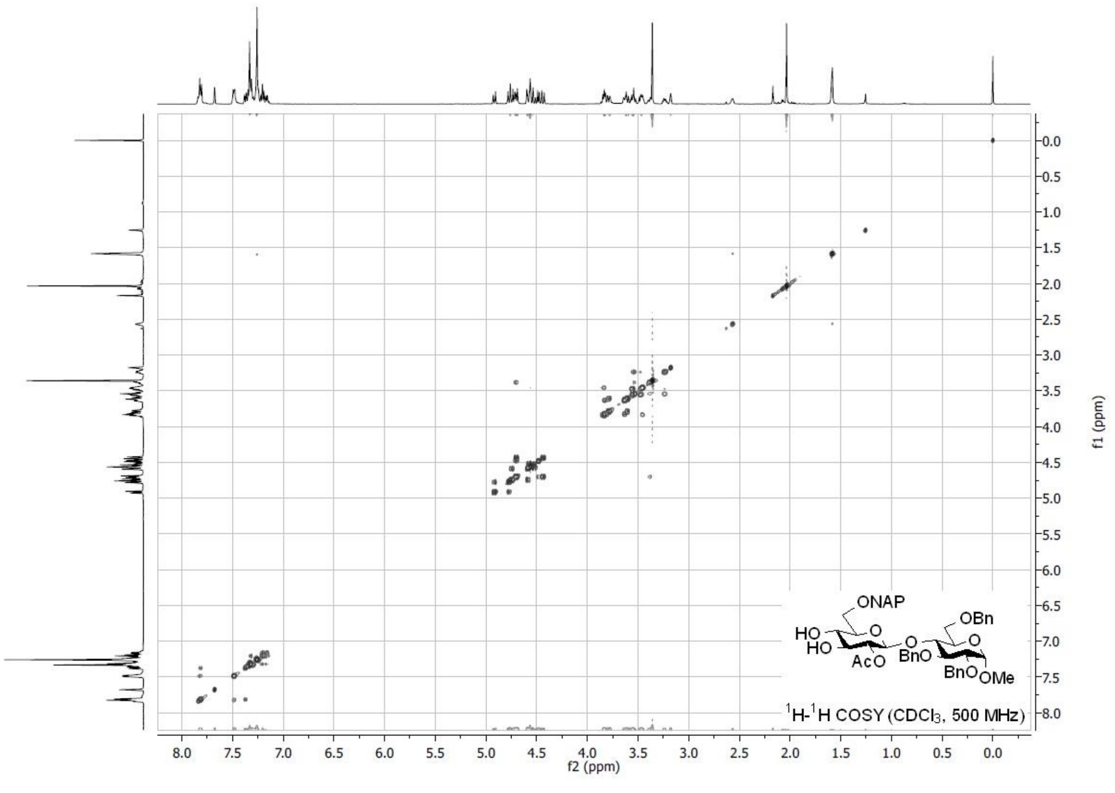

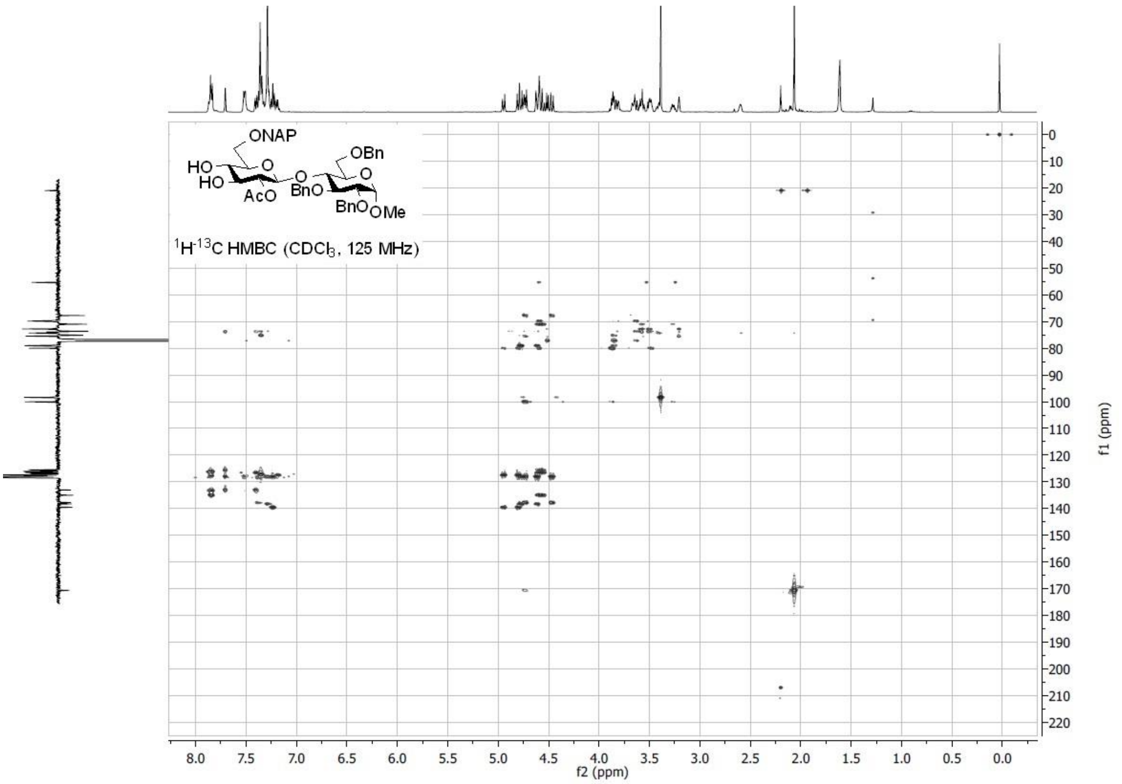

Figure S11. ^{1}H, ^{13}C, COSY and HSQC NMR spectra of compound **21**.

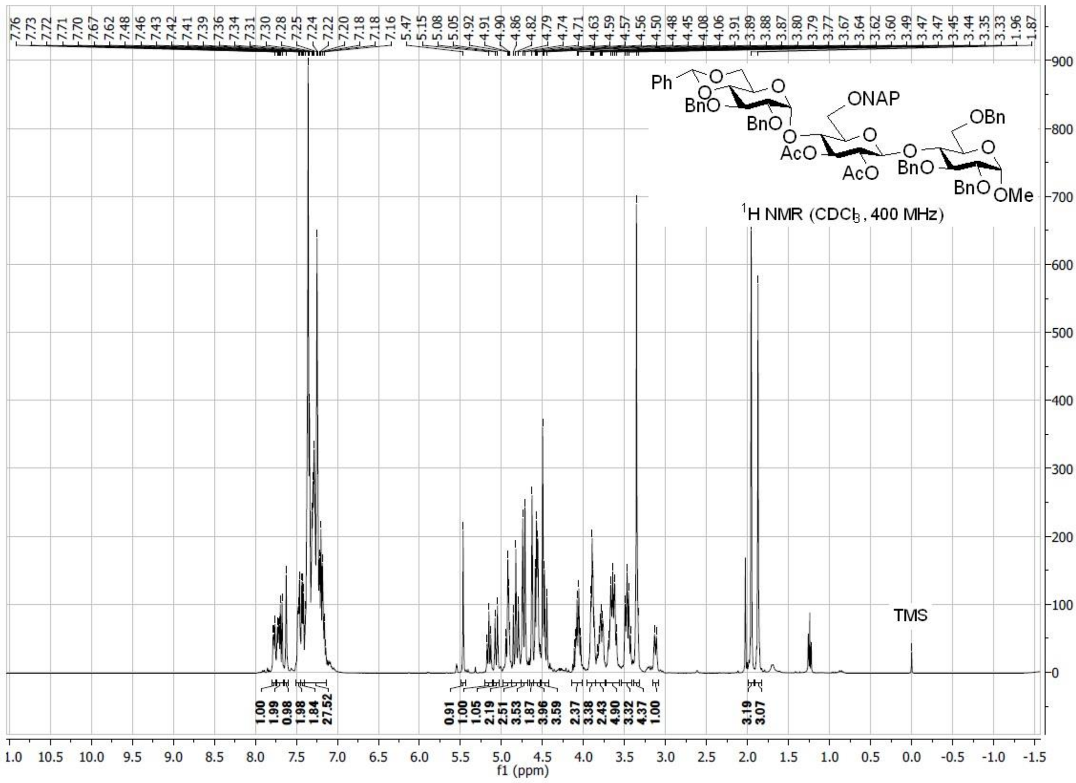
ONAP
OBn
Ph
BnO
AcO
OMe
^{1}H NMR (CDCl$_3$, 400 MHz)
TMS
f1 (ppm)

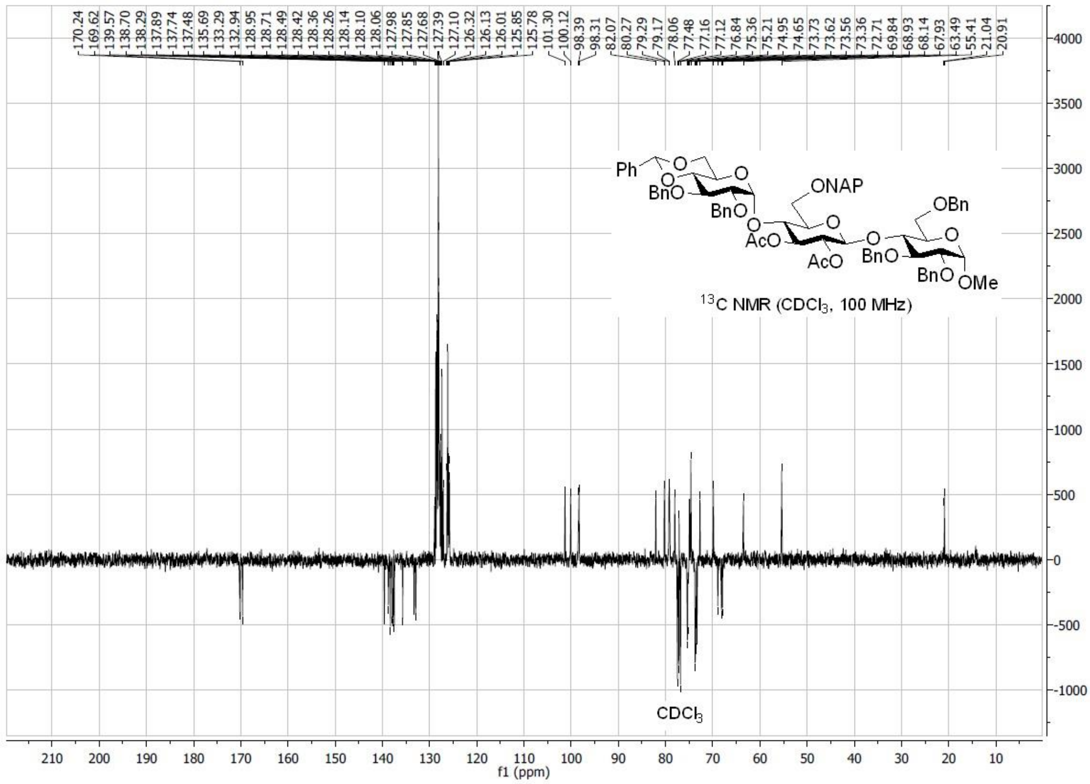
ONAP
OBn
Ph
BnO
AcO
OMe
^{13}C NMR (CDCl$_3$, 100 MHz)
CDCl$_3$
f1 (ppm)

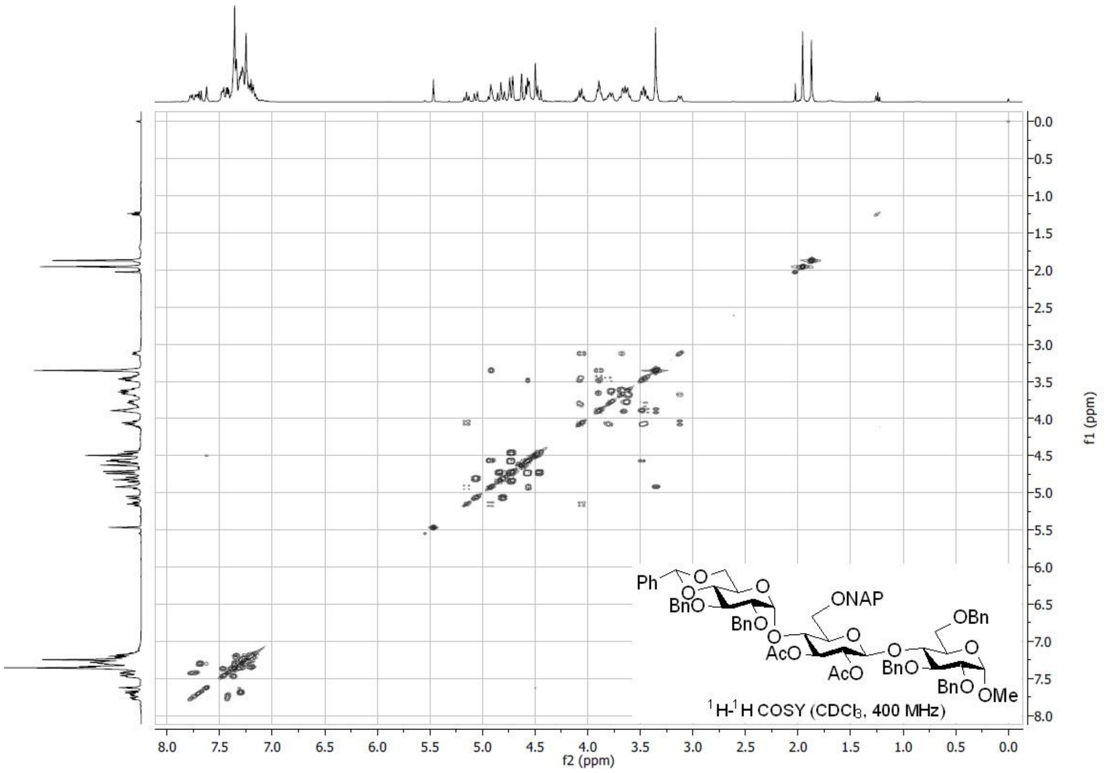

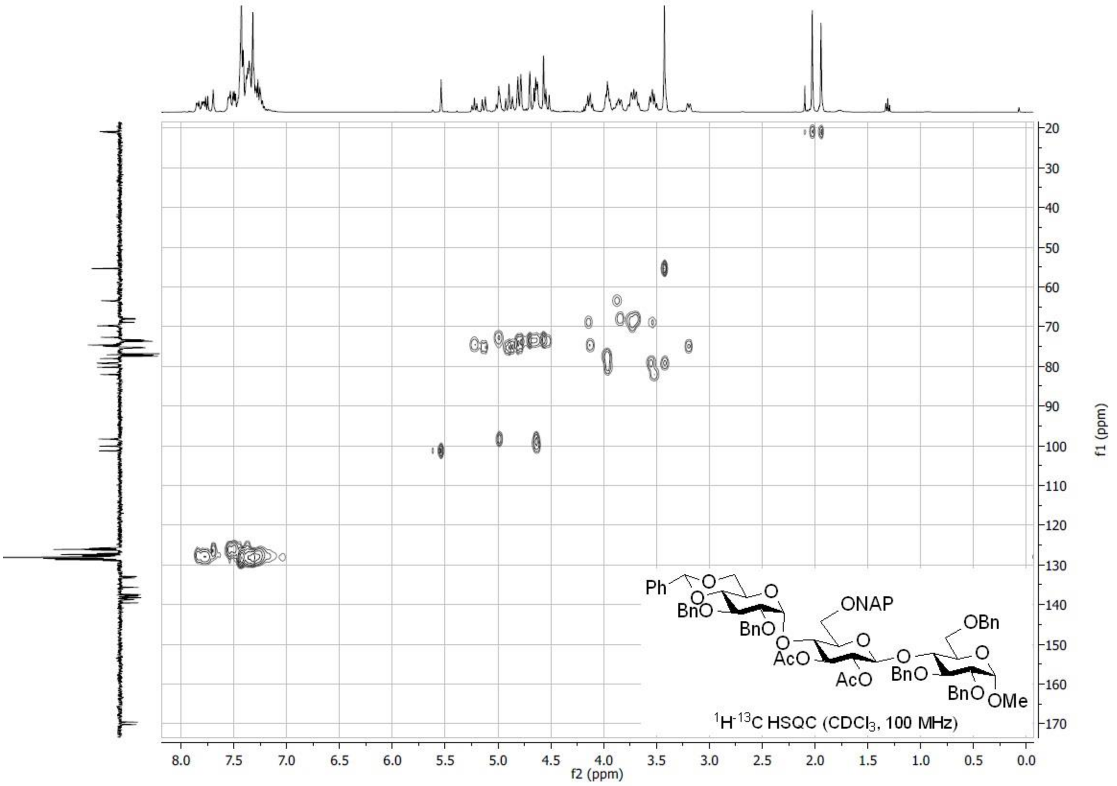

Figure S12. ^{1}H, ^{13}C, COSY and HSQC NMR spectra of compound **25**.

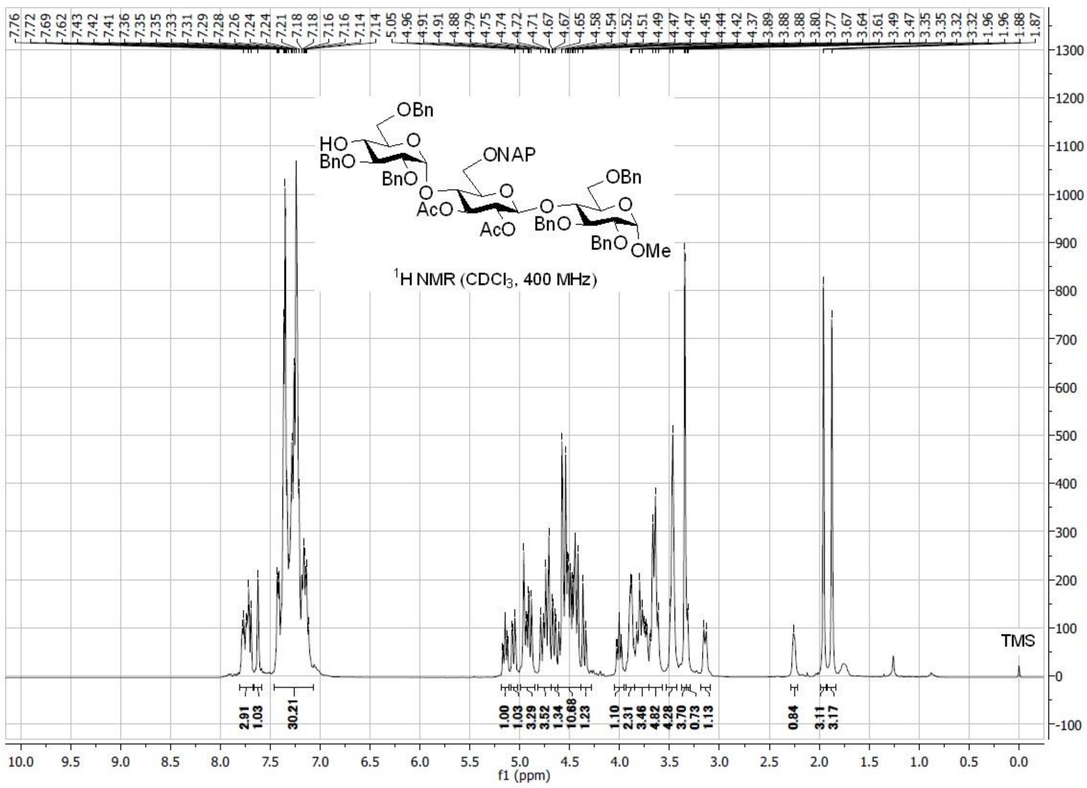

1H NMR (CDCl3, 400 MHz)
TMS
f1 (ppm)

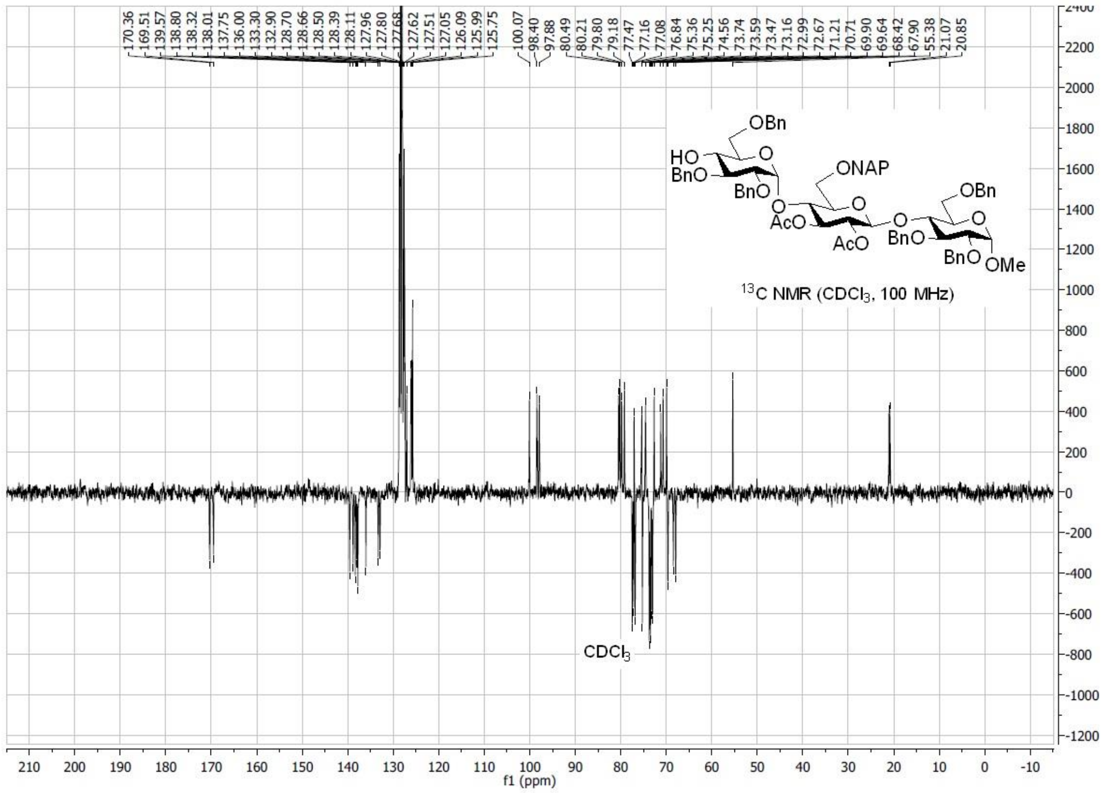

13C NMR (CDCl3, 100 MHz)
CDCl3
f1 (ppm)

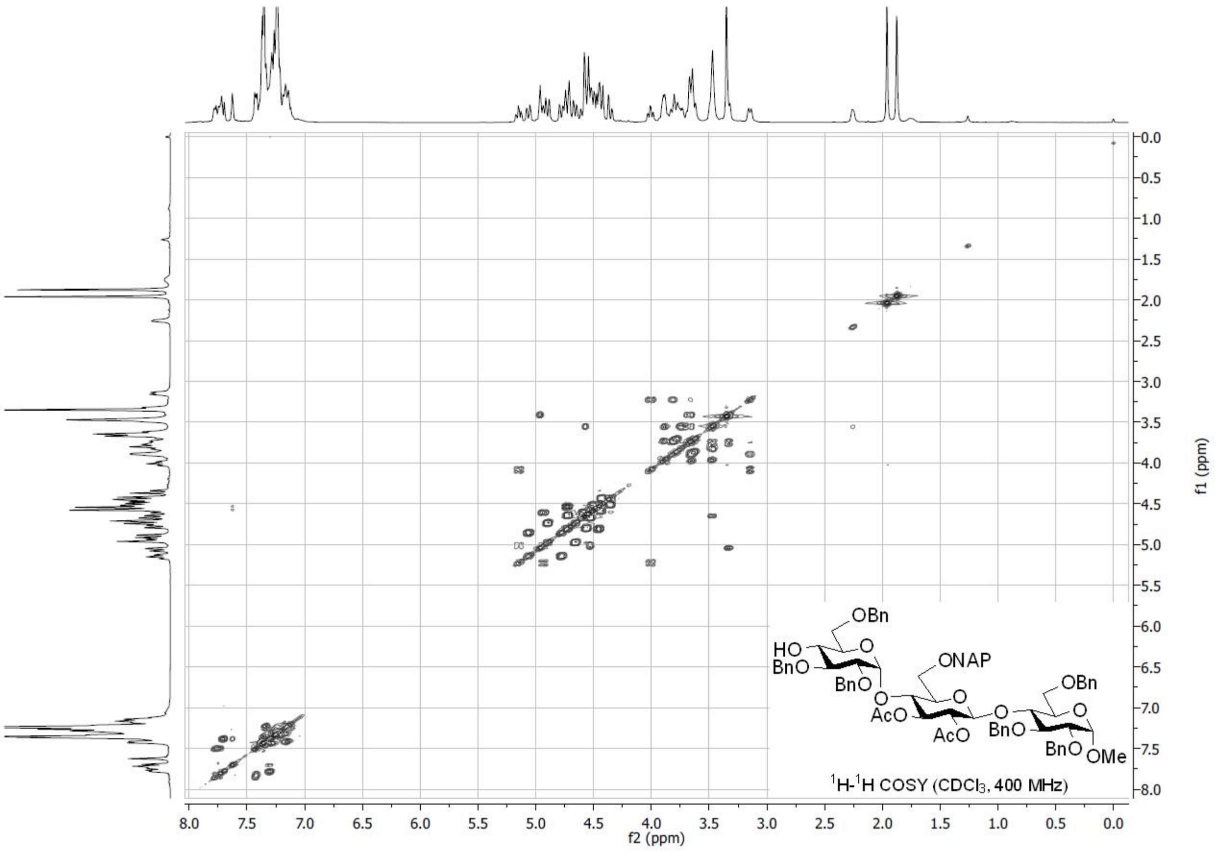

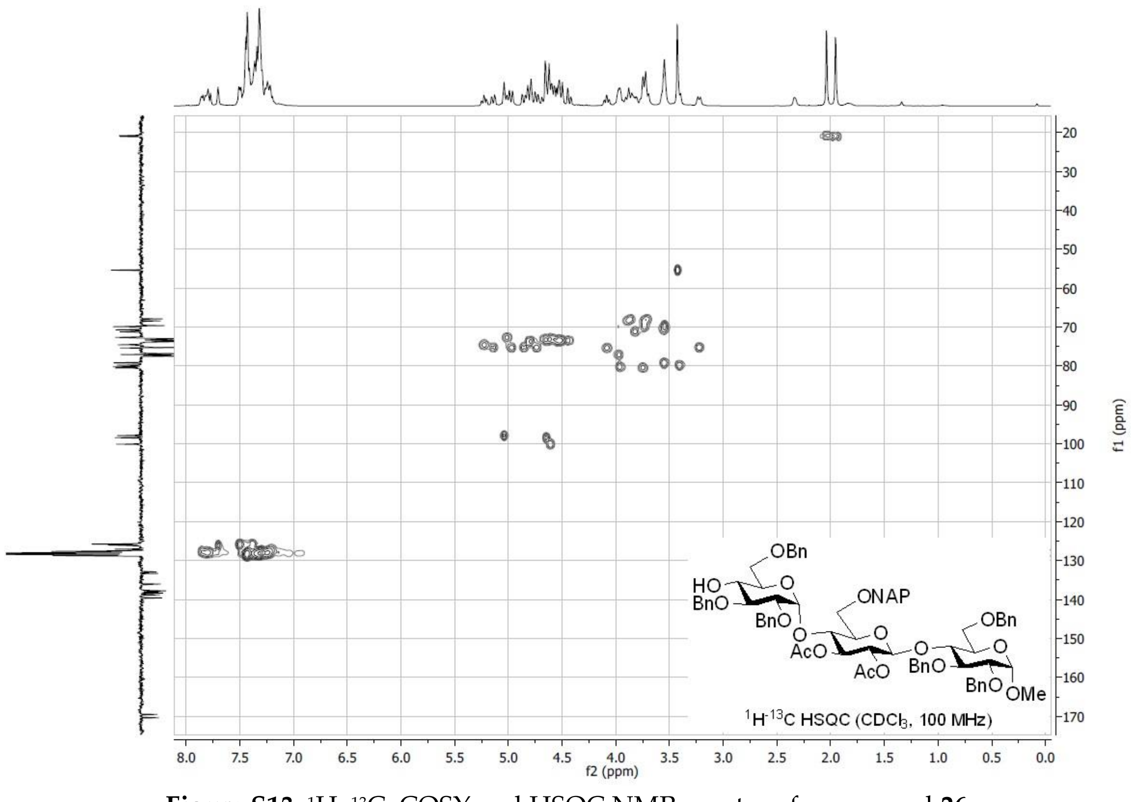

Figure S13. ^{1}H, ^{13}C, COSY and HSQC NMR spectra of compound **26**.

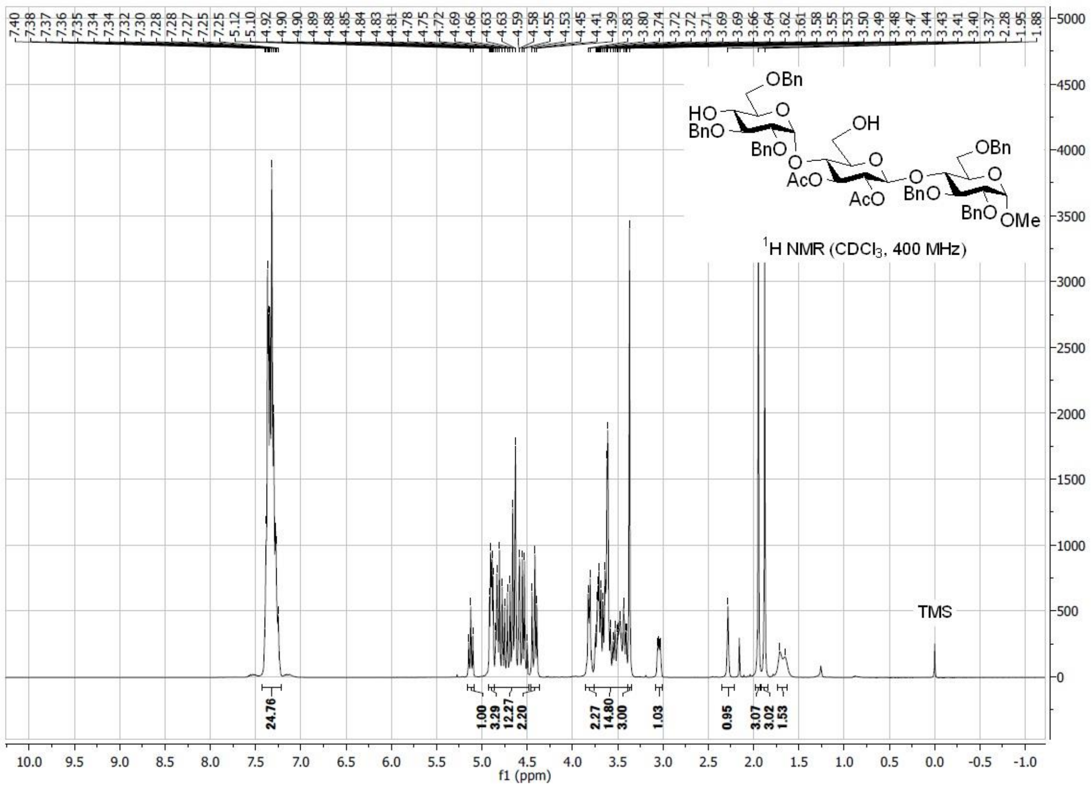

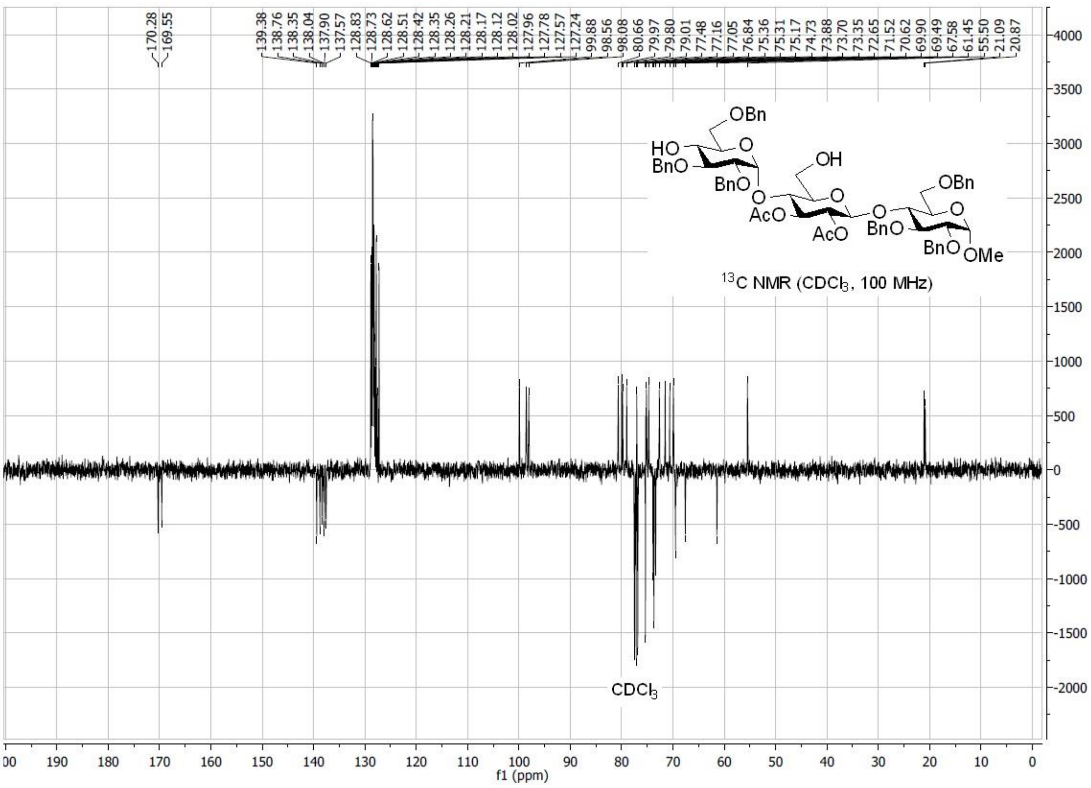

Figure S14. ^{1}H, ^{13}C NMR spectra of compound **27**.

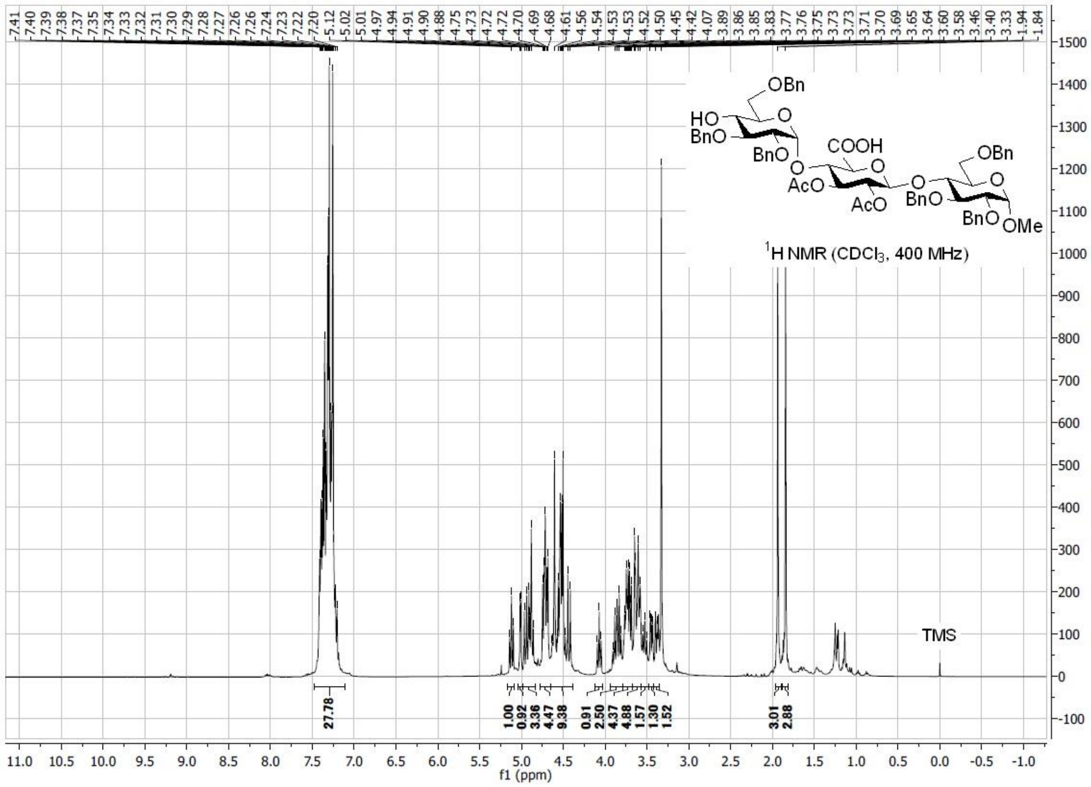

¹H NMR (CDCl3, 400 MHz)
TMS
f1 (ppm)

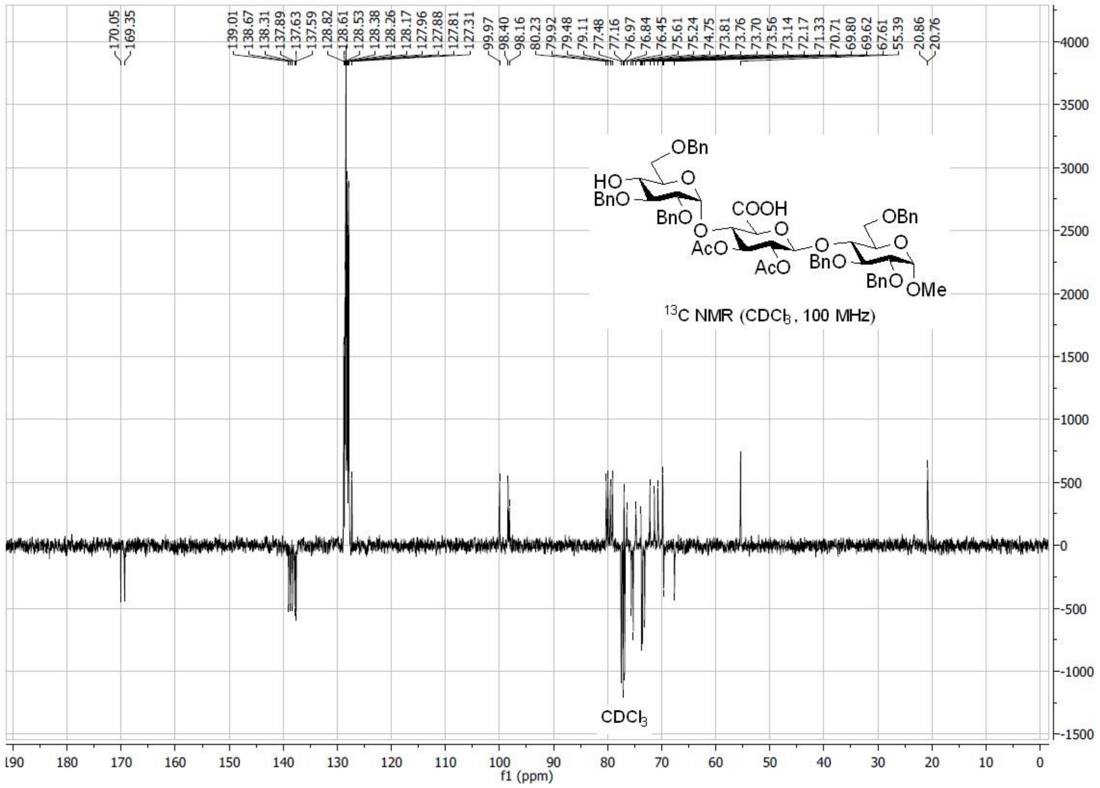

¹³C NMR (CDCl3, 100 MHz)
CDCl3
f1 (ppm)

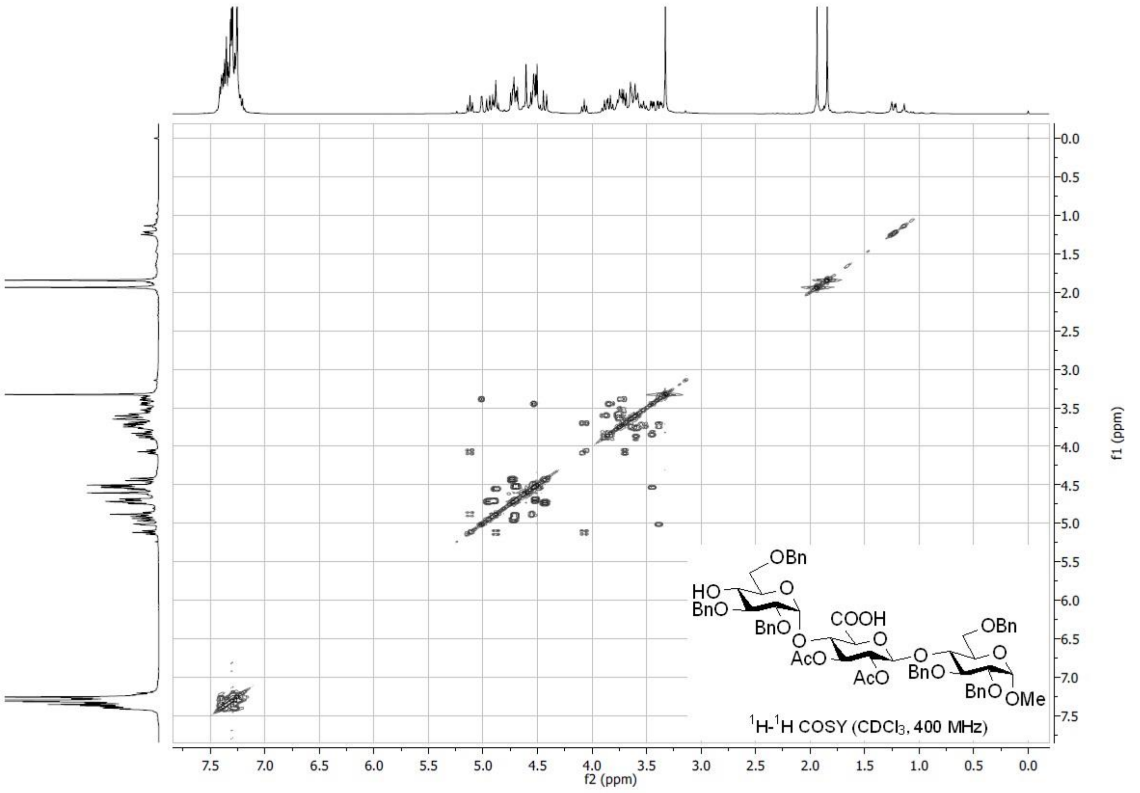

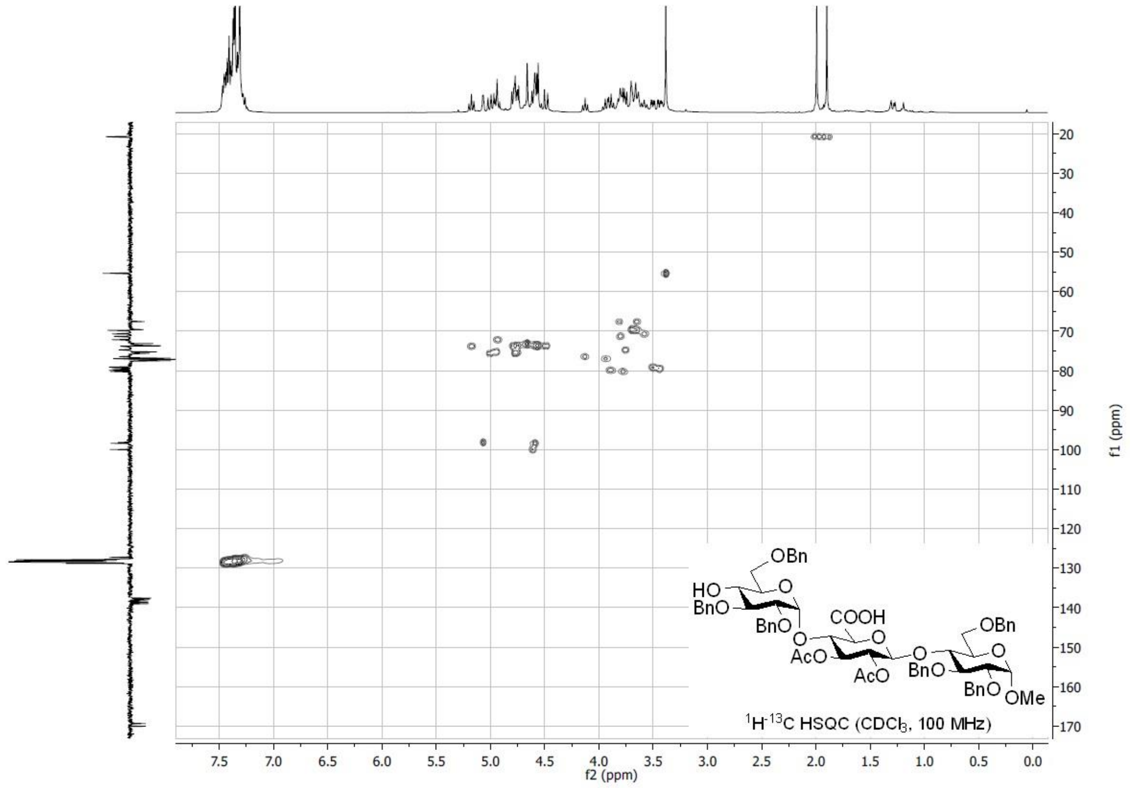

Figure S15. ^{1}H, ^{13}C, COSY and HSQC NMR spectra of compound **28**.

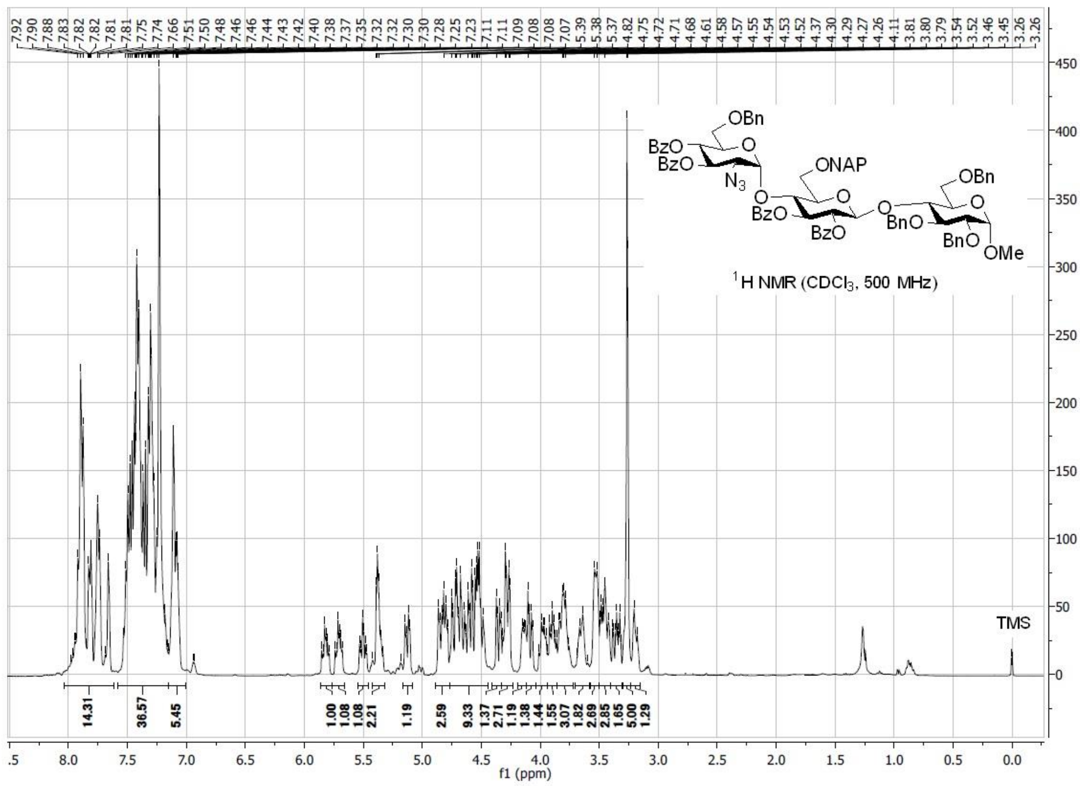

BzO
BzO
OBn
O
N3
ONAP
O
O
BzO
BzO
O
OBn
O
BnO
BnO
OMe
^{1}H NMR (CDCl3, 500 MHz)
TMS
f1 (ppm)

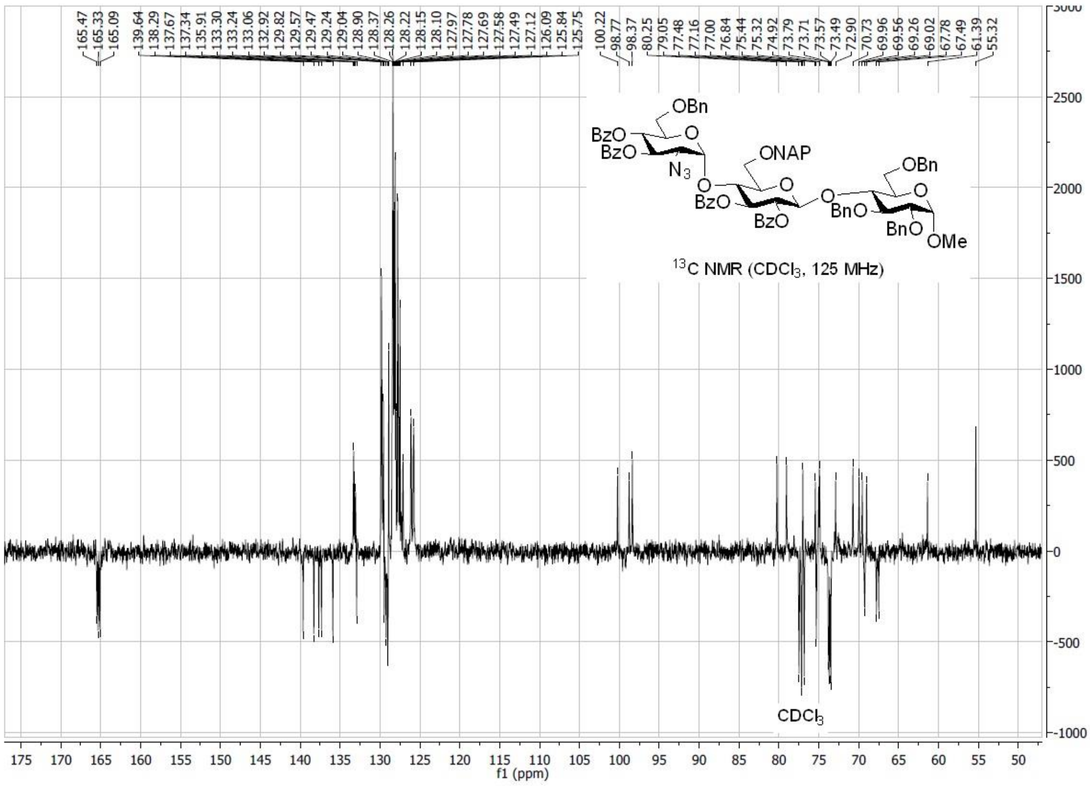

^{13}C NMR (CDCl3, 125 MHz)
CDCl3
f1 (ppm)

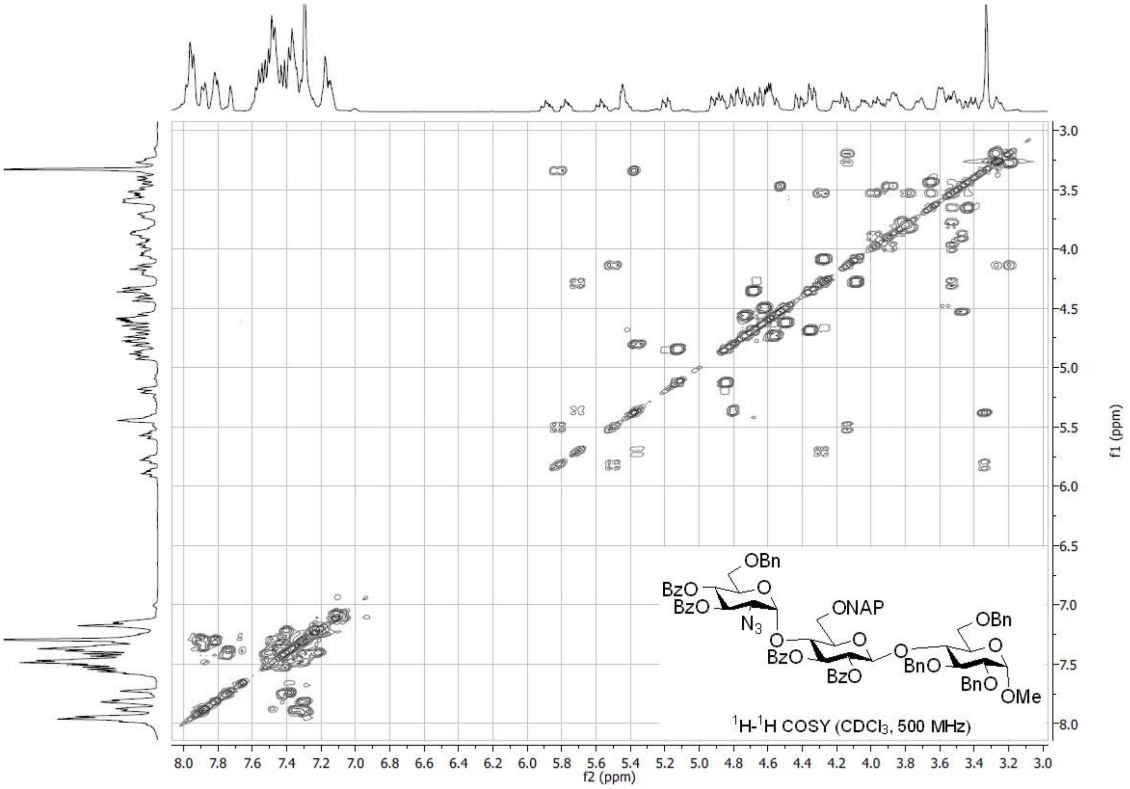

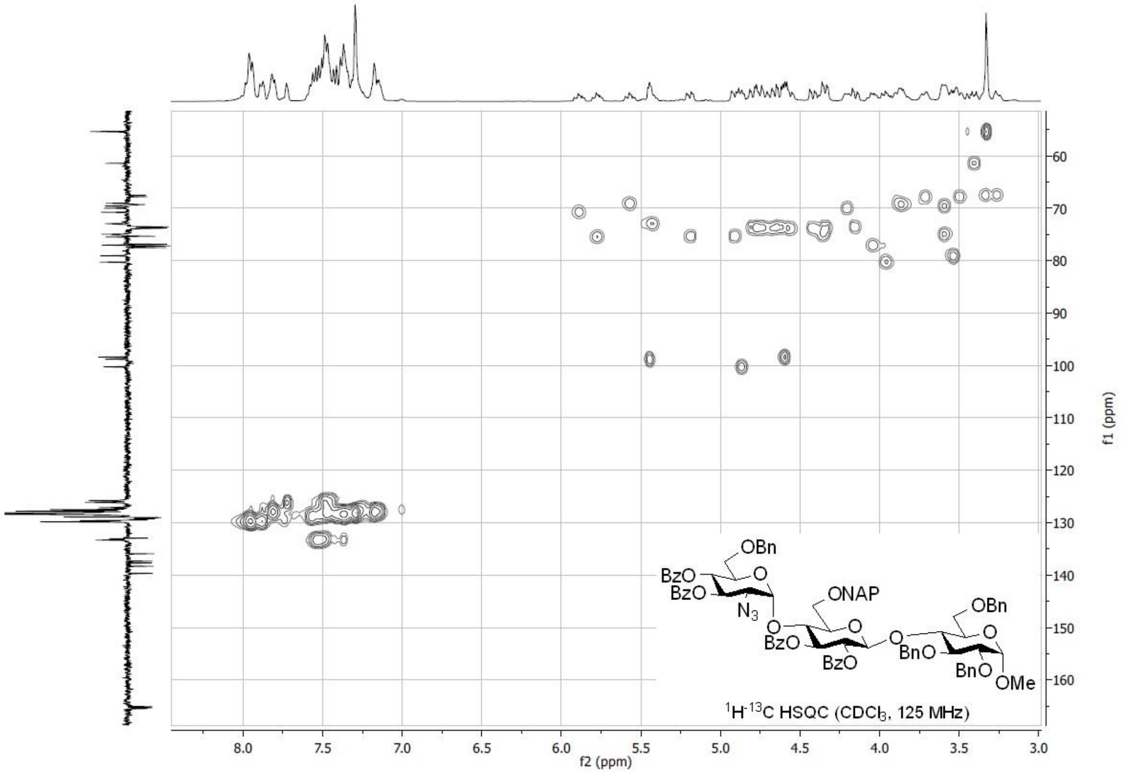

Figure S16. ^{1}H, ^{13}C, COSY and HSQC NMR spectra of compound **30**.

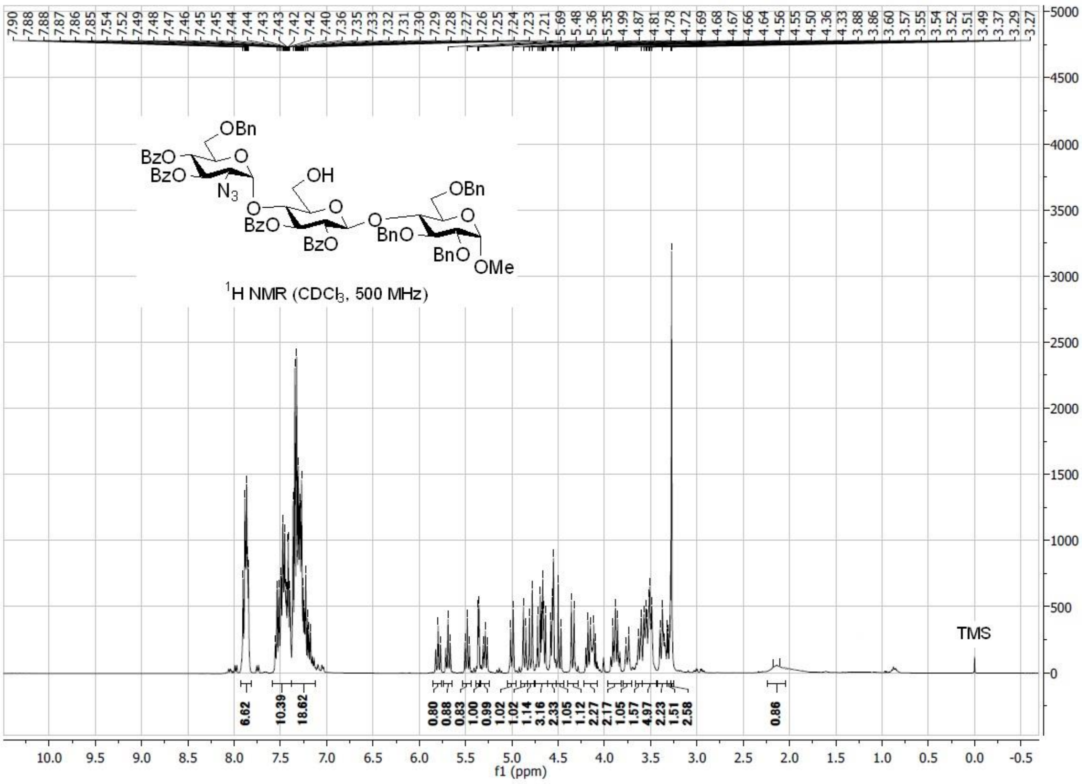
7.90 7.88 7.88 7.87 7.86 7.85 7.54 7.52 7.49 7.48 7.47 7.46 7.45 7.45 7.44 7.44 7.43 7.43 7.42 7.42 7.40 7.36 7.35 7.33 7.32 7.31 7.30 7.29 7.28 7.27 7.26 7.25 7.24 7.23 7.21 5.69 5.48 5.36 5.35 4.99 4.87 4.81 4.78 4.72 4.69 4.68 4.67 4.66 4.64 4.56 4.55 4.50 4.36 4.33 3.88 3.86 3.60 3.57 3.55 3.54 3.52 3.51 3.49 3.37 3.29 3.27
OBn
BzO
BzO
O
N3
OH
O
OBn
O
BzO
BzO
O
BnO
BnO
O
OMe
1H NMR (CDCl3, 500 MHz)
TMS
6.62 10.39 18.82 0.80 0.88 0.83 1.00 0.99 1.02 1.02 1.14 3.16 2.33 1.05 1.12 2.27 2.17 1.05 1.57 4.97 2.23 1.51 2.58 0.86
f1 (ppm)

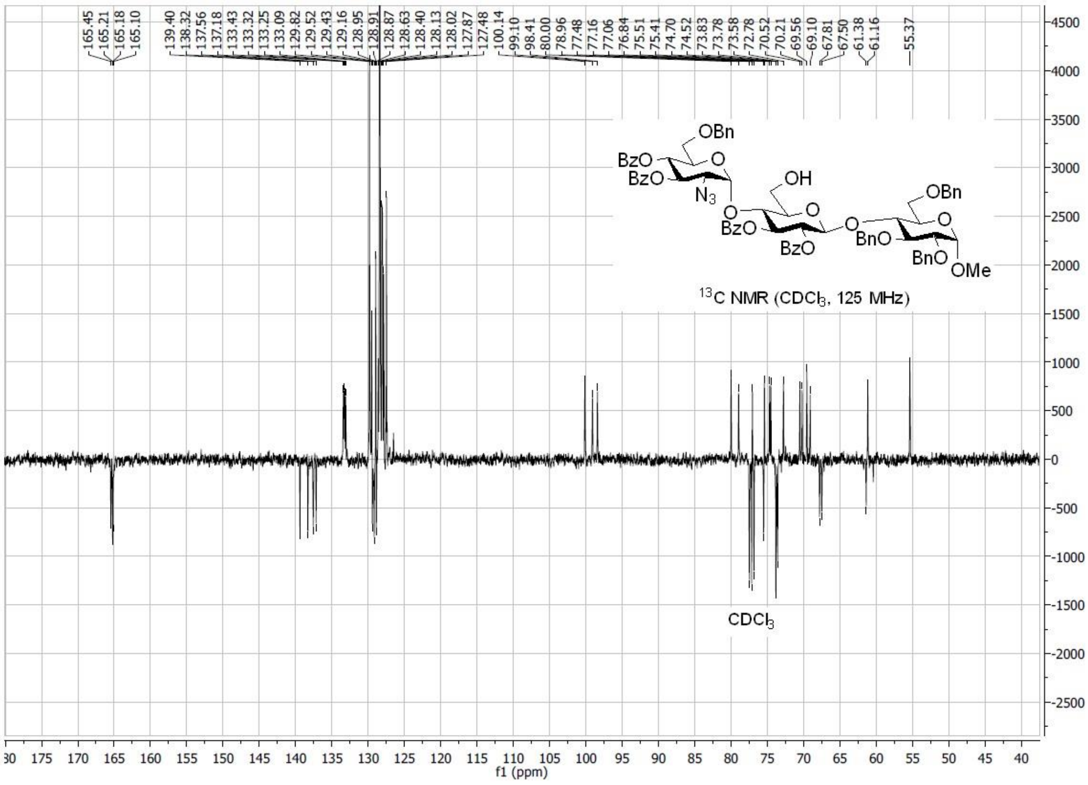
165.45 165.21 165.18 165.10 139.40 138.32 137.56 137.18 133.43 133.32 133.25 133.09 129.82 129.52 129.43 129.16 128.95 128.91 128.87 128.63 128.40 128.13 128.02 127.87 127.48 100.14 99.10 98.41 80.00 78.96 77.48 77.16 77.06 76.84 75.51 75.41 74.70 74.52 73.83 73.78 73.58 72.78 70.52 70.21 69.56 69.10 67.81 67.50 61.38 61.16 55.37
OBn
BzO
BzO
O
N3
OH
O
OBn
O
BzO
BzO
O
BnO
BnO
O
OMe
13C NMR (CDCl3, 125 MHz)
CDCl3
f1 (ppm)

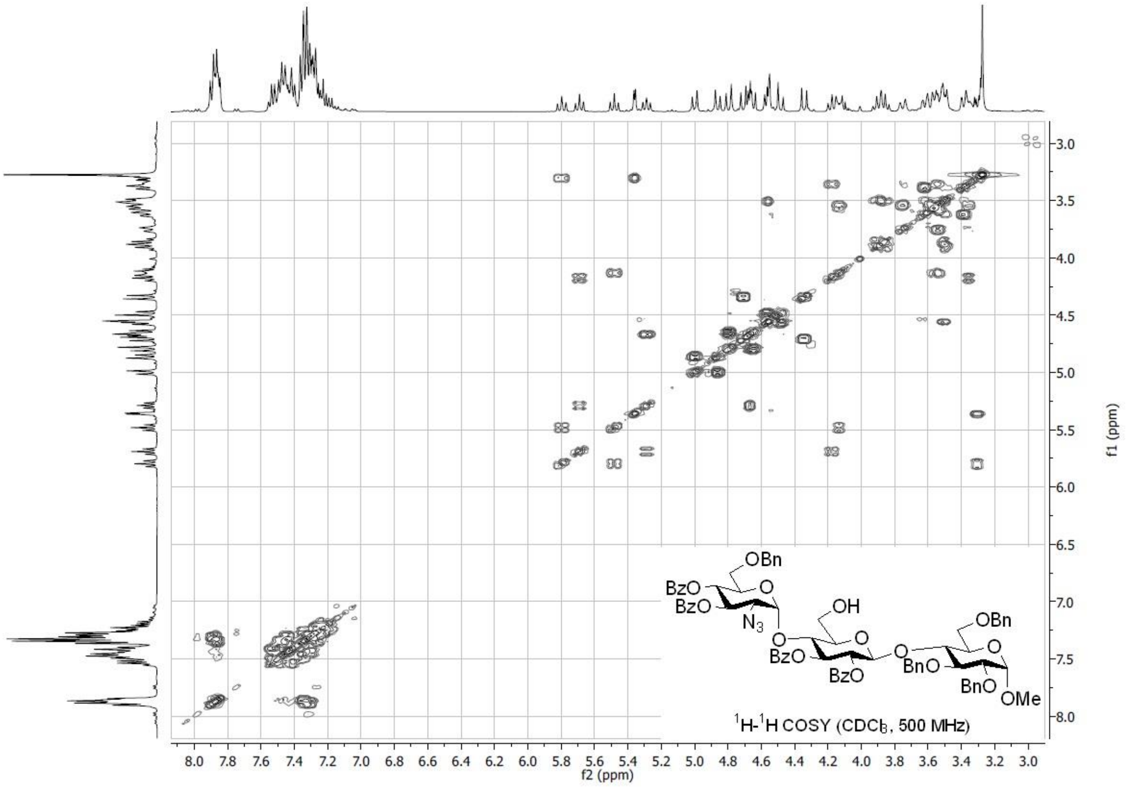

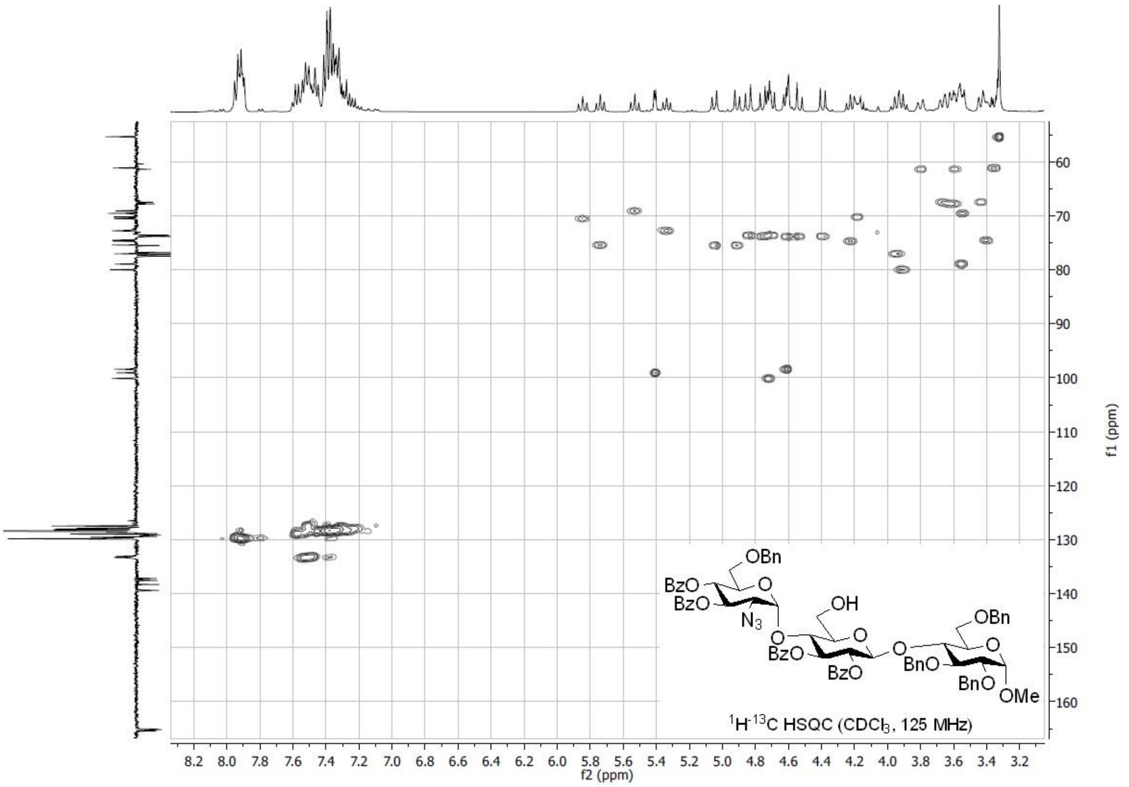

Figure S17. ^{1}H, ^{13}C, COSY and HSQC NMR spectra of compound **31**.

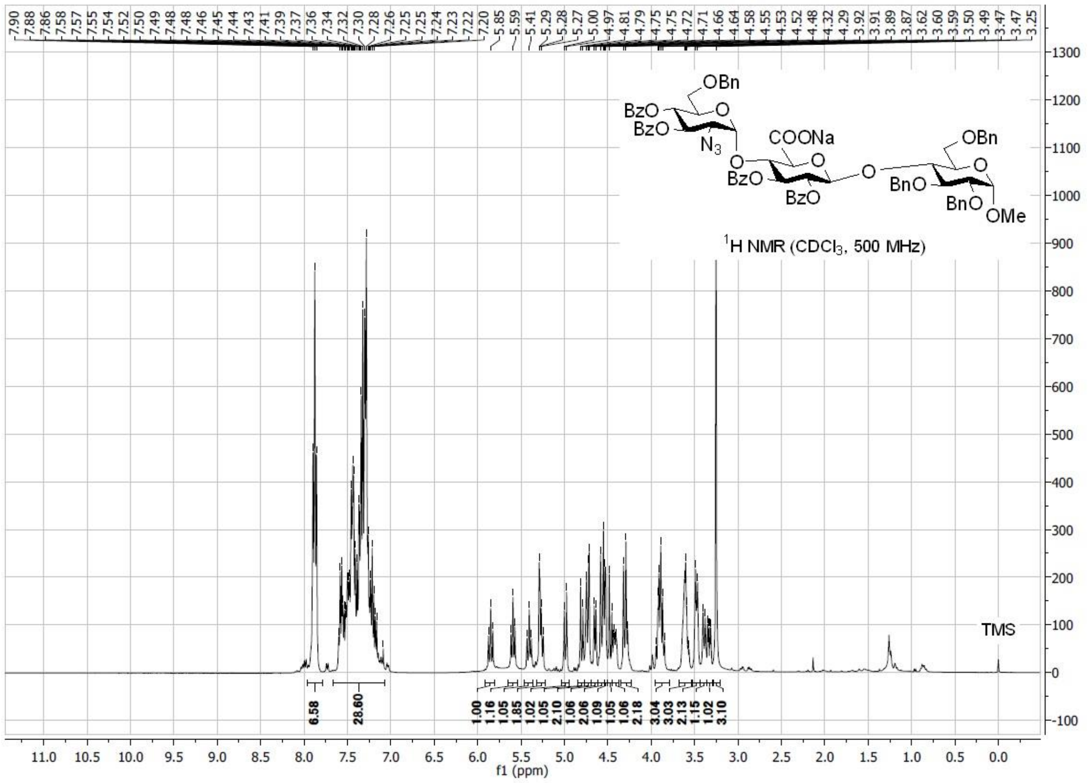

OBn
BzO
BzO
N3
COONa
BzO
BzO
OBn
BnO
BnO
OMe
1H NMR (CDCl3, 500 MHz)
TMS
f1 (ppm)

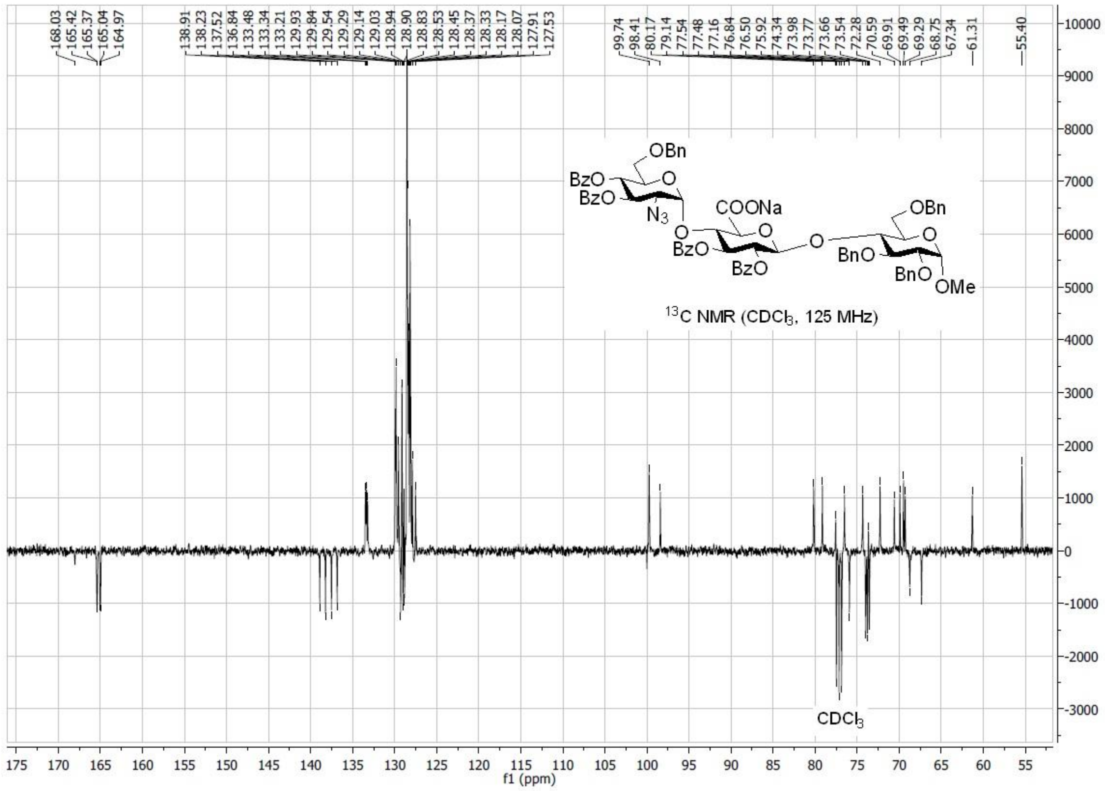

OBn
BzO
BzO
N3
COONa
BzO
BzO
OBn
BnO
BnO
OMe
13C NMR (CDCl3, 125 MHz)
CDCl3
f1 (ppm)

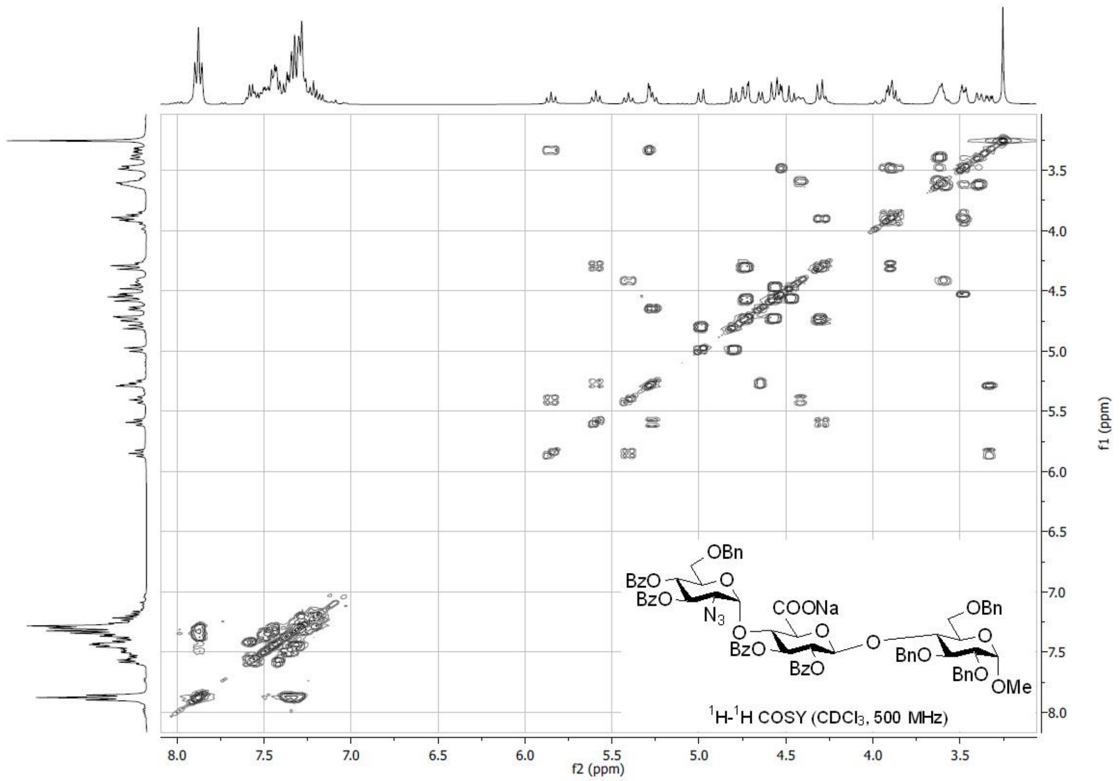

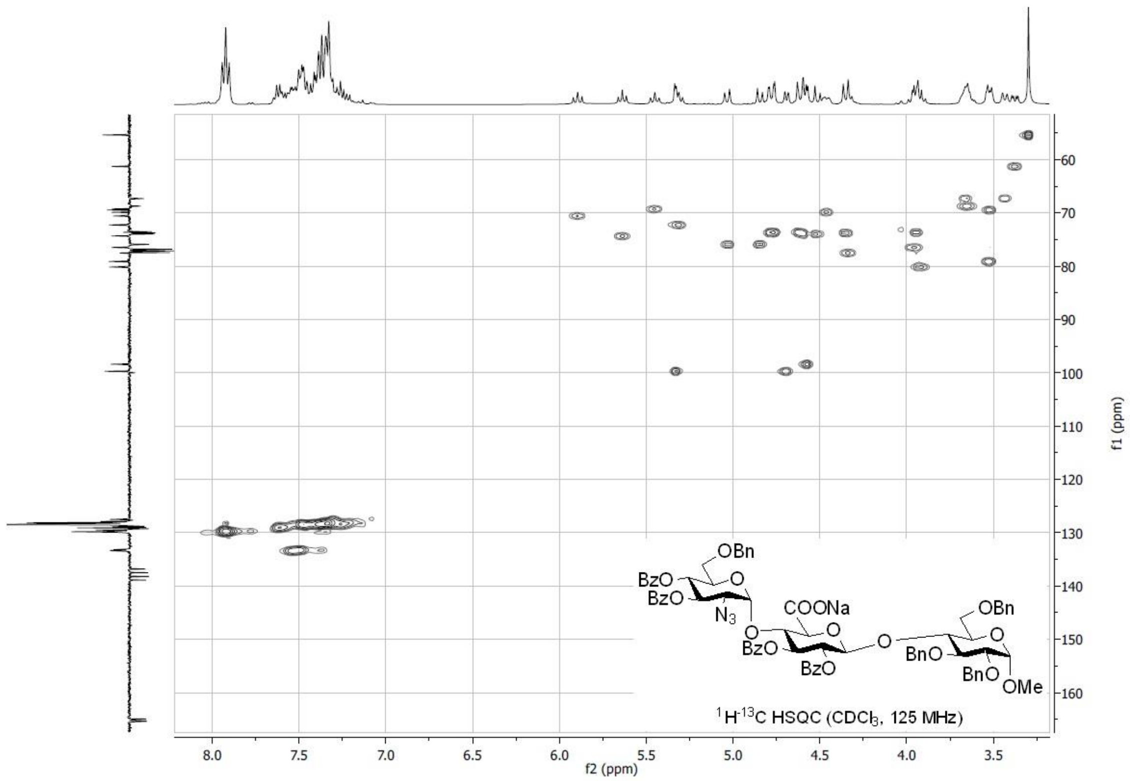

Figure S18. ^{1}H, ^{13}C, COSY and HSQC NMR spectra of compound **32**.

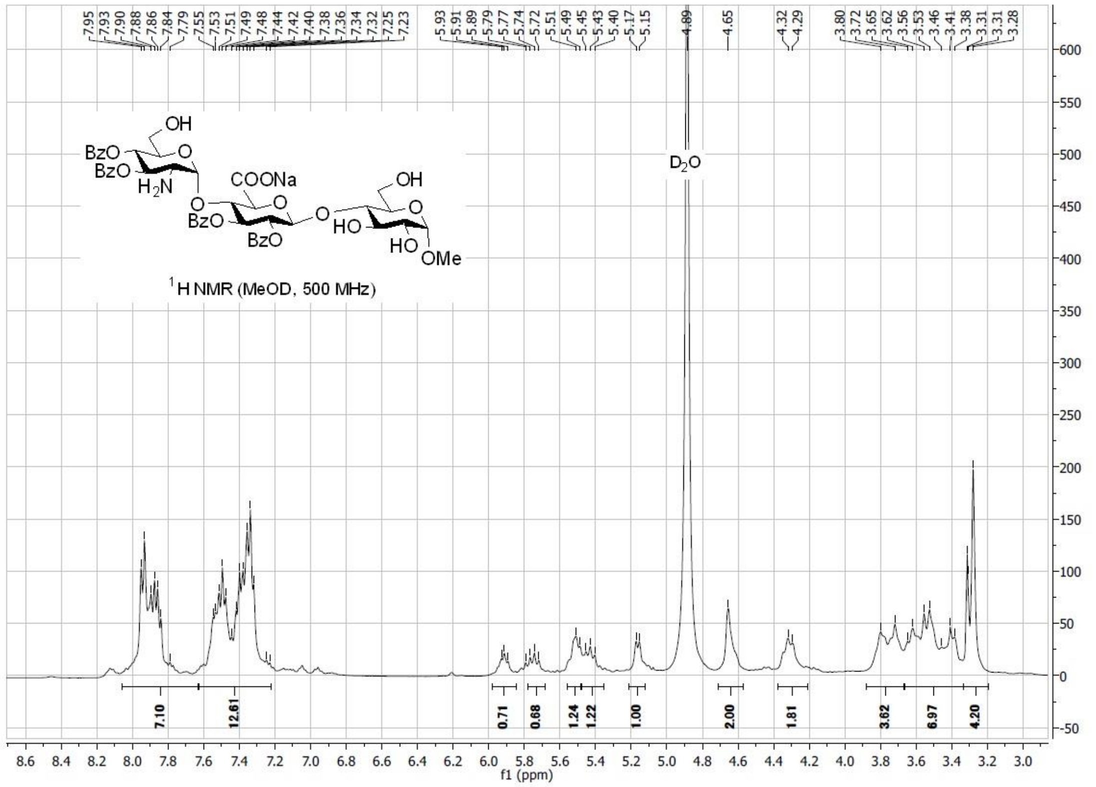
7.95 7.93 7.90 7.88 7.86 7.84 7.79 7.55 7.53 7.51 7.49 7.48 7.44 7.42 7.40 7.38 7.36 7.34 7.32 7.25 7.23
5.93 5.91 5.89 5.79 5.77 5.74 5.72 5.51 5.49 5.45 5.43 5.40 5.17 5.15
4.89 4.65 4.32 4.29
3.80 3.72 3.65 3.62 3.56 3.53 3.46 3.41 3.38 3.31 3.28
BzO
BzO
OH
H2N
COONa
OH
BzO
HO
BzO
HO
OMe
D2O
1H NMR (MeOD, 500 MHz)
7.10 12.61 0.71 0.68 1.24 1.22 1.00 2.00 1.81 3.82 6.97 4.20
f1 (ppm)

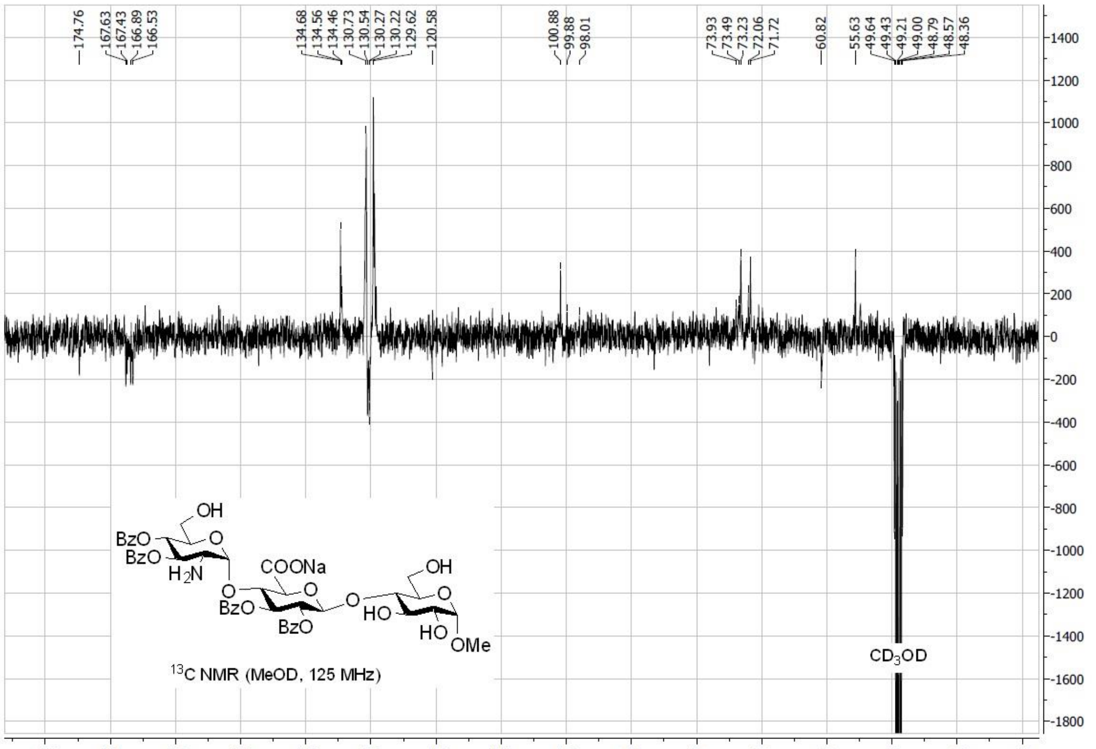
174.76 167.63 167.43 166.89 166.53
134.68 134.56 134.46 130.73 130.54 130.27 130.22 129.62 120.58
100.88 99.88 98.01
73.93 73.49 73.23 72.06 71.72
60.82 55.63 49.64 49.43 49.21 49.00 48.79 48.57 48.36
BzO
BzO
OH
H2N
COONa
OH
BzO
HO
BzO
HO
OMe
13C NMR (MeOD, 125 MHz)
CD3OD
f1 (ppm)

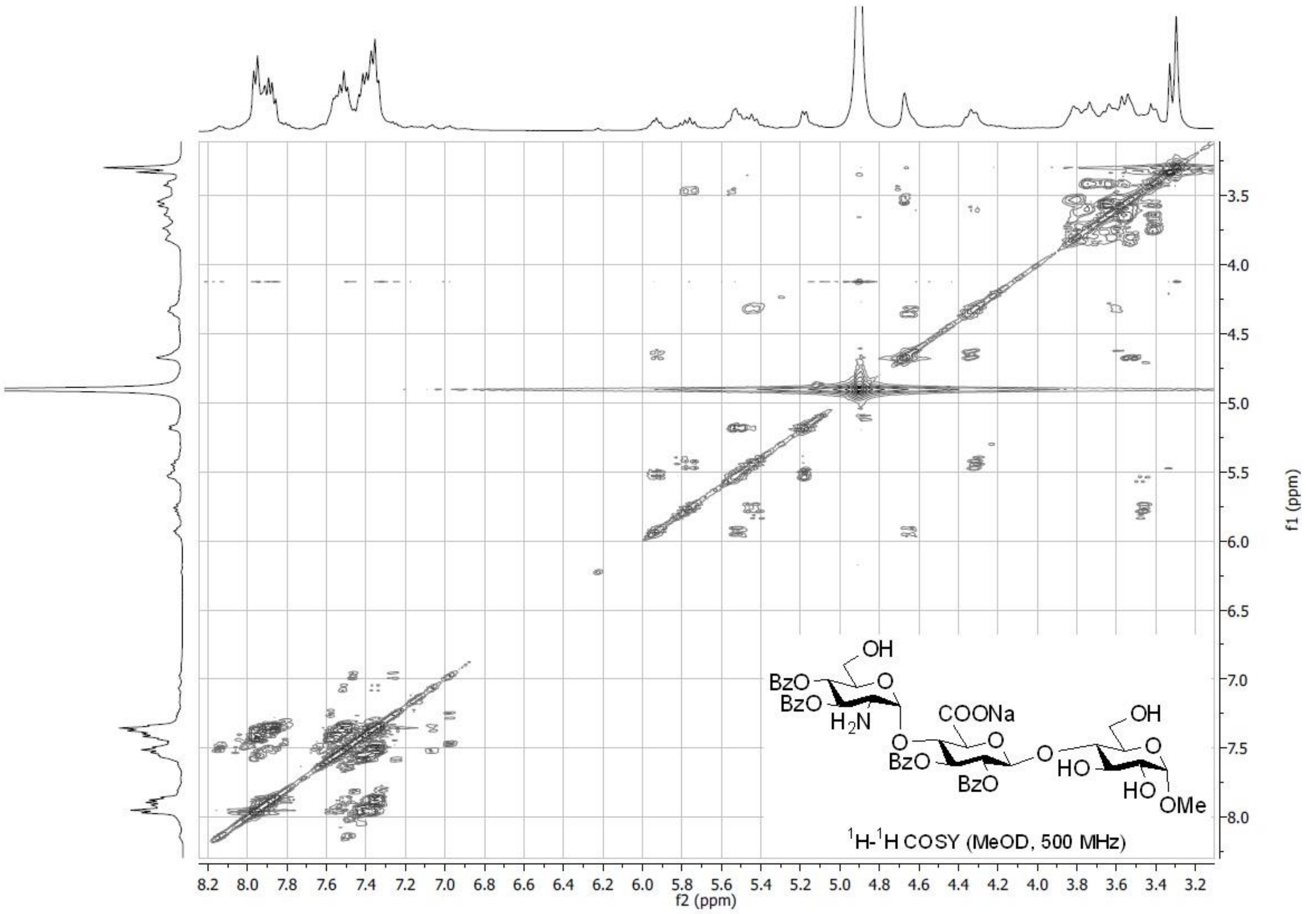

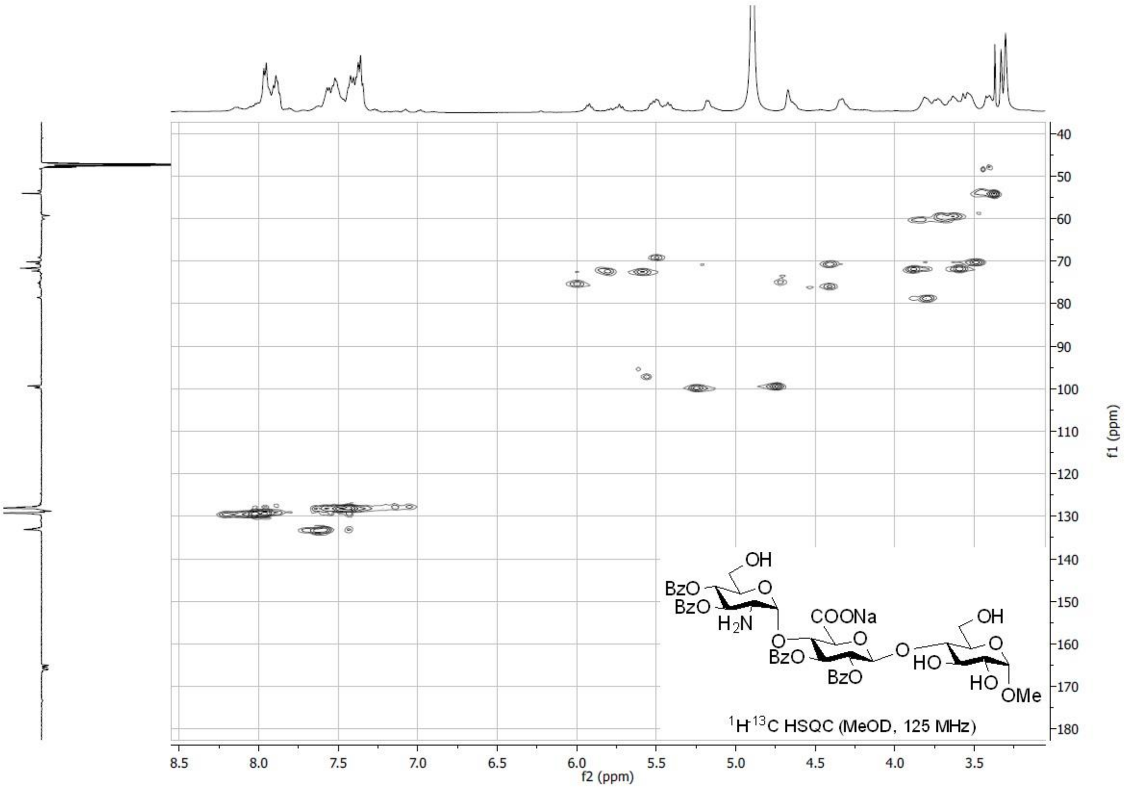

Figure S19. ^{1}H, ^{13}C, COSY and HSQC NMR spectra of compound **33**.
